# PATIENT-REPORTED OUTCOME MEASURES FOR DEPRESSIVE SYMPTOMS IN ADULTS WITH SPINAL CORD INJURY: A COSMIN SYSTEMATIC REVIEW

**DOI:** 10.2340/jrm.v58.46626

**Published:** 2026-10-28

**Authors:** Runlin LU, Yixin DUN, Lingzhi LI, Bing HU, Caihong ZHANG

**Affiliations:** 1 Department of Spine and Orthopedics, Tianyou Hospital Affiliated to Wuhan University of Science and Technology, Wuhan, Hubei; 2 Liyuan Hospital of Tongji Medical College of Huazhong University of Science and Technology, Wuhan, Hubei, China

**Keywords:** depression, patient-reported outcome measures, psychometrics, spinal cord injuries

## Abstract

**Objective:**

To evaluate the measurement properties of patient-reported measures of depressive symptoms in adults with spinal cord injury and inform their purpose-specific selection and interpretation in clinical and research settings.

**Design:**

Systematic review.

**Subjects/Patients:**

Adults with traumatic or non-traumatic spinal cord injury.

**Methods:**

10 databases were searched from inception to 25 June 2026, with reference checking, citation tracking, instrument websites, and targeted searches. Two reviewers independently selected reports, extracted data, assessed risk of bias, rated measurement properties, and graded certainty using current international guidance for outcome measurement instruments.

**Results:**

28 reports evaluated 27 separately scored versions, forms, or depression subscales from 14 instrument families. The Spinal Cord Injury–Quality of Life Depression Item Bank had the strongest condition-specific development and internal-structure evidence, but evidence gaps and insufficient measurement invariance prevented recommendation. The English Patient Health Questionnaire-9 had sufficient case-finding evidence but insufficient structural validity for interpreting its conventional total score as a clearly unidimensional severity measure. The English depression subscale of the Hospital Anxiety and Depression Scale showed sufficient structural validity, internal consistency, and construct validity. Evidence for measurement error and responsiveness was scarce.

**Conclusion:**

No measure had sufficiently complete evidence for routine use across screening, severity assessment, and change measurement.

## LAY ABSTRACT

People with spinal cord injury often experience depression, but physical effects of the injury can resemble depressive symptoms and make questionnaire scores difficult to interpret. We reviewed studies of questionnaires completed by adults with spinal cord injury and examined how well each questionnaire measures depressive symptoms. No questionnaire had enough strong evidence for routine use for every purpose. The Spinal Cord Injury–Quality of Life Depression measure had the strongest development work for assessing symptom severity. The Patient Health Questionnaire may help identify people who need a fuller clinical assessment, but its total score should be interpreted cautiously. The depression part of the Hospital Anxiety and Depression Scale may be useful when clinicians wish to limit questions concerning physical symptoms. More research is especially needed to determine whether score changes represent meaningful improvement or worsening.

Spinal cord injury is a major cause of long-term disability worldwide and continues to impose a substantial global health burden (1–3). Depression is a clinically important secondary condition after spinal cord injury (SCI). Approximately 1 in 5 adults with SCI experience major depressive disorder (4). Depressive symptoms are associated with poorer participation in rehabilitation, daily activities, and social roles, as well as reduced quality of life and adverse health outcomes (5, 6). These aspects are particularly relevant to rehabilitation and community reintegration after SCI. Routine assessment is recommended during inpatient rehabilitation and long-term follow-up (6).

Patient-reported outcome measures (PROMs) capture patients’ perspectives on symptoms, but their scores require evidence of validity for the intended population and context of use (7). Measurement can be challenging after SCI because symptoms such as sleep disturbance, fatigue, appetite change, psychomotor slowing, concentration difficulty, and reduced activity may reflect depression, SCI-related physical effects, secondary medical conditions, or environmental factors (8, 9). Excluding all somatic symptoms, however, may narrow the construct and miss clinically meaningful depressive presentations. A suitable instrument must therefore capture depressive symptom burden without automatically attributing disability-related manifestations to depression (8, 9).

Previous reviews identified substantial heterogeneity in depression measures used after SCI and limited evidence for diagnostic accuracy, responsiveness, feasibility, and interpretability (8–10). They pre-dated several large factor-analytic studies, SCI-specific item-bank development, differential item functioning analyses, responsiveness studies, and the 2024 revision of the COSMIN methodology. They also did not consistently separate study risk of bias, the sufficiency of measurement-property results, and the certainty of summarized evidence.

The updated COSMIN framework provides a transparent sequence for evaluating PROMs: assessment of study risk of bias, rating of individual results against explicit criteria, synthesis by PROM version and measurement property, grading of certainty, and formulation of recommendations for a defined population and purpose (11). PRISMA-COSMIN for OMIs 2024 further supports complete and reproducible reporting (12). We therefore conducted a COSMIN systematic review to evaluate depressive-symptom PROMs in adults with SCI and provide purpose-specific evidence interpretation while keeping diagnostic screening distinct from evaluative measurement and change assessment.

## METHODS

### Protocol and reporting

The review was conducted according to the COSMIN guideline for systematic reviews of PROMs version 2.0 and reported in accordance with PRISMA-COSMIN for OMIs 2024 (11, 12). The review question specified the outcome domain of depressive symptoms, adults with SCI as the target population, PROMs as the type of outcome measurement instrument, and the measurement properties of interest. The completed PRISMA-COSMIN for OMIs 2024 and PRISMA 2020 checklists are submitted as supporting information. The protocol was registered in PROSPERO (CRD420261436775).

### Eligibility criteria

Full-text original studies were eligible if they evaluated PROM development or at least 1 COSMIN measurement property of a self-reported depressive-symptom measure in adults with traumatic or non-traumatic SCI. Studies explicitly described as adult SCI cohorts were retained when the lower age threshold extended marginally below 18 years, provided that they were not designed for paediatric populations; potential age-related indirectness was considered during certainty assessment. Eligible instruments were not prespecified but were identified during study screening according to the eligibility criteria already mentioned; these included generic and SCI-specific PROMs, short forms, item banks, translated versions, and independently scored depression subscales. Mixed-condition studies were included only when SCI-specific results were reported separately or were extractable. For multidimensional instruments, only a distinct depression subscale or explicitly derived depression score was considered. Interpretability and feasibility were extracted when available but were not treated as measurement properties. We excluded paediatric or predominantly adolescent studies without separable adult data; studies that used a PROM only to estimate prevalence, examine associations, or evaluate treatment outcomes without assessing a COSMIN measurement property; studies without separable SCI- or depression-specific data; and clinician-rated, diagnostic-interview, or proxy measures as target instruments. Diagnostic interviews could serve as reference standards.

### Information sources and search strategy

PubMed/MEDLINE, Embase, Web of Science, Scopus, CINAHL, PsycINFO, CNKI, Wanfang Data, VIP, and SinoMed/CBM were searched from database inception to 25 June 2026. Additional sources included reference-list checking, forward citation tracking, the COSMIN database, instrument-owner or instrument-developer websites, and targeted supplementary searches for named PROMs and relevant development or validation reports. No database language filter was applied; however, searches were conducted using English- and Chinese-language terms, and full-text eligibility was restricted to reports available in English or Chinese. Search strategies were adapted to the indexing terms, fields, and interfaces of each database and combined terms for SCI, depressive symptoms, PROMs, and measurement properties. The broader terms “mood disorder” and “dysphori*” were included to maximize search sensitivity and capture potentially relevant records with variable or older indexing; they did not broaden the review question or eligibility criteria. For PubMed/MEDLINE, the validated sensitive search filter for measurement properties developed by Terwee and colleagues was adapted and combined with SCI-, depression-, and PROM-related terms (13). The complete database-specific strategies are provided in Table SI.

### Study selection

After duplicate removal, Runlin Lu and Yixin Dun independently screened titles and abstracts and then assessed potentially eligible full-text reports. Disagreements were resolved through discussion, with unresolved decisions adjudicated by Bing Hu. Lingzhi Li checked the screening records and full-text exclusion coding for consistency. Multiple reports from the same or potentially overlapping cohorts were linked and retained only when they contributed unique measurement-property evidence. Overlapping or repeated samples were not treated as independent precision contributions during synthesis.

### Data extraction

Runlin Lu and Yixin Dun independently extracted report and population characteristics, including publication year, participant age and sex distribution, SCI characteristics, and time since injury or injury phase when reported, as well as PROM version and language, administration mode, recall period, scoring, comparator instruments, methods, numerical results, and information on interpretability and feasibility.

Each language version, subscale, short form, item bank, and administration form was treated as a distinct PROM when its evidence or intended use differed. For PROM-level characteristics, the original development year and version history were extracted where identifiable. *Post hoc* research scores were counted separately when an independently scored result and corresponding measurement-property evidence were reported. Reports were linked at cohort level to identify duplicate or overlapping evidence. Data were managed in a purpose-built workbook based on the COSMIN review-management structure (11) and were reported using the official table templates (14). Current access conditions, including licensing, registration, fees, and translation availability, were additionally verified from official rights-holder, developer, or distributor sources where available; these were treated as implementation information rather than COSMIN measurement properties.

### Risk of bias

Before formal assessment, the reviewers familiarized themselves with the COSMIN manuals and independently piloted the checklist on a sample of studies and resolved discrepancies. They then independently assessed each eligible analysis-result unit using the COSMIN Risk of Bias checklist version 3.0. Relevant standards were rated very good, adequate, doubtful, or inadequate, and the overall rating was determined by the worst-score-counts principle. Disagreements were resolved by discussion and, when necessary, adjudicated by Bing Hu; Lingzhi Li checked the completed assessment records for consistency (11).

### Rating of measurement-property results

All measurement-property ratings, summarized conclusions, and certainty assessments were independently completed by Runlin Lu and Yixin Dun; disagreements were resolved by consensus and, when necessary, adjudicated by Bing Hu. For content validity, the reviewers additionally assessed the relevance, comprehensiveness, and comprehensibility of each PROM version/form using the available instructions, items, response options, recall period, and intended context of use. Consensus reviewer ratings were integrated with eligible evidence from PROM-development and content-validity studies.

Individual results were rated sufficient (+), insufficient (−), or indeterminate (?) using the COSMIN criteria for good measurement properties version 2.0 (11). Structural validity was evaluated separately for classical test theory and item response theory or Rasch analyses. Internal consistency was considered sufficient only when at least low-certainty evidence supported sufficient structural validity and Cronbach’s α or an analogous coefficient was ≥ 0.70. Reliability required an intraclass correlation coefficient (ICC), weighted κ, or another appropriate reliability coefficient of ≥ 0.70. Measurement error was sufficient when the smallest detectable change or limits of agreement were smaller than a minimal important change. Criterion validity required a correlation with a reasonable gold standard or an area under the receiver operating characteristic curve of ≥ 0.70. Construct validity and responsiveness were rated against explicit hypotheses. When study authors had not prespecified hypotheses, the reviewers formulated the expected direction and, where feasible, magnitude before examining the relevant results; disagreements were resolved by consensus. If an *a priori* hypothesis could not be specified, the result was rated indeterminate.

Structured diagnostic interviews were accepted as reference standards for classifying major depressive disorder, but not as gold standards for continuous symptom severity. Because this was not a diagnostic test accuracy review, QUADAS-2 and diagnostic-accuracy certainty methods were not applied; sensitivity, specificity, and cut-off values were treated as supplementary descriptive evidence and did not determine formal COSMIN Step 7 categories. Criterion validity was rated indeterminate when neither an eligible area under the curve nor a correlation with the diagnostic reference standard was available. Comparisons of brief or derived forms with an overlapping parent PROM were not treated as independent criterion-validity evidence.

### Evidence synthesis and certainty assessment

Results were synthesized by PROM version/form and measurement property. For content validity, study-based evidence and consensus reviewer ratings were integrated separately for relevance, comprehensiveness, and comprehensibility; all other properties were synthesized from eligible empirical results. Meta-analysis was not undertaken because of heterogeneity in study designs, populations, scoring models, comparators, and potential cohort overlap. Overlapping samples were linked and were not treated as independent contributions to precision. Consistent findings yielded a summarized rating of sufficient, insufficient, or indeterminate. Unresolved discordance was rated inconsistent (±) after considering methodological quality, sample independence, setting, injury phase, language version of the PROM, and psychometric model.

Certainty of evidence was graded as high, moderate, low, or very low using the COSMIN-modified GRADE approach (11). Downgrading considered risk of bias, inconsistency, imprecision, and indirectness; publication bias was not formally assessed. Indeterminate findings were not graded, and certainty for internal consistency did not exceed that for structural validity.

### Formal recommendation framework and purpose-specific interpretation

Formal COSMIN Step 7 conclusions were assigned by PROM version/form and prespecified application. A PROM was recommended for use only when high-certainty evidence supported sufficient results for all relevant measurement properties. A PROM was advised against when high-certainty evidence indicated an insufficient result for at least 1 measurement property relevant to the intended interpretation. In all other cases, no firm conclusion was drawn. Supplementary diagnostic findings, feasibility, respondent burden, language availability, and interpretability informed purpose-specific considerations but did not alter the formal COSMIN conclusion (11).

## RESULTS

### Study selection and characteristics

The final evidence set comprised 28 reports published between 1988 and 2019 (15–42). The study-selection process is shown in the PRISMA-COSMIN flow diagram (Fig. 1), and full-text exclusions are detailed in Table SII. The 78 records identified through supplementary methods yielded no additional eligible report after deduplication and eligibility assessment. No eligible measurement-property report published after 2019 was identified. Across 14 instrument families, the reports evaluated 27 separately scored PROM versions, administration forms, or depression subscales. Report-level principal SCI samples ranged from 39 to 7,296; property-specific subsamples were as small as 20. Twenty-one reports were conducted in the United States, 2 in Canada, and 1 each in the United Kingdom, Australia, Switzerland, Türkiye (Turkey), and Lebanon. The included studies represented heterogeneous SCI stages, ranging from initial inpatient rehabilitation to long-term community follow-up, with time since injury ranging from days to decades when reported. Most studies enrolled adults across broad age ranges, whereas few specifically targeted older adults. Characteristics of the included PROMs and reports are presented in Tables I and II, respectively.

**Fig. 1:**
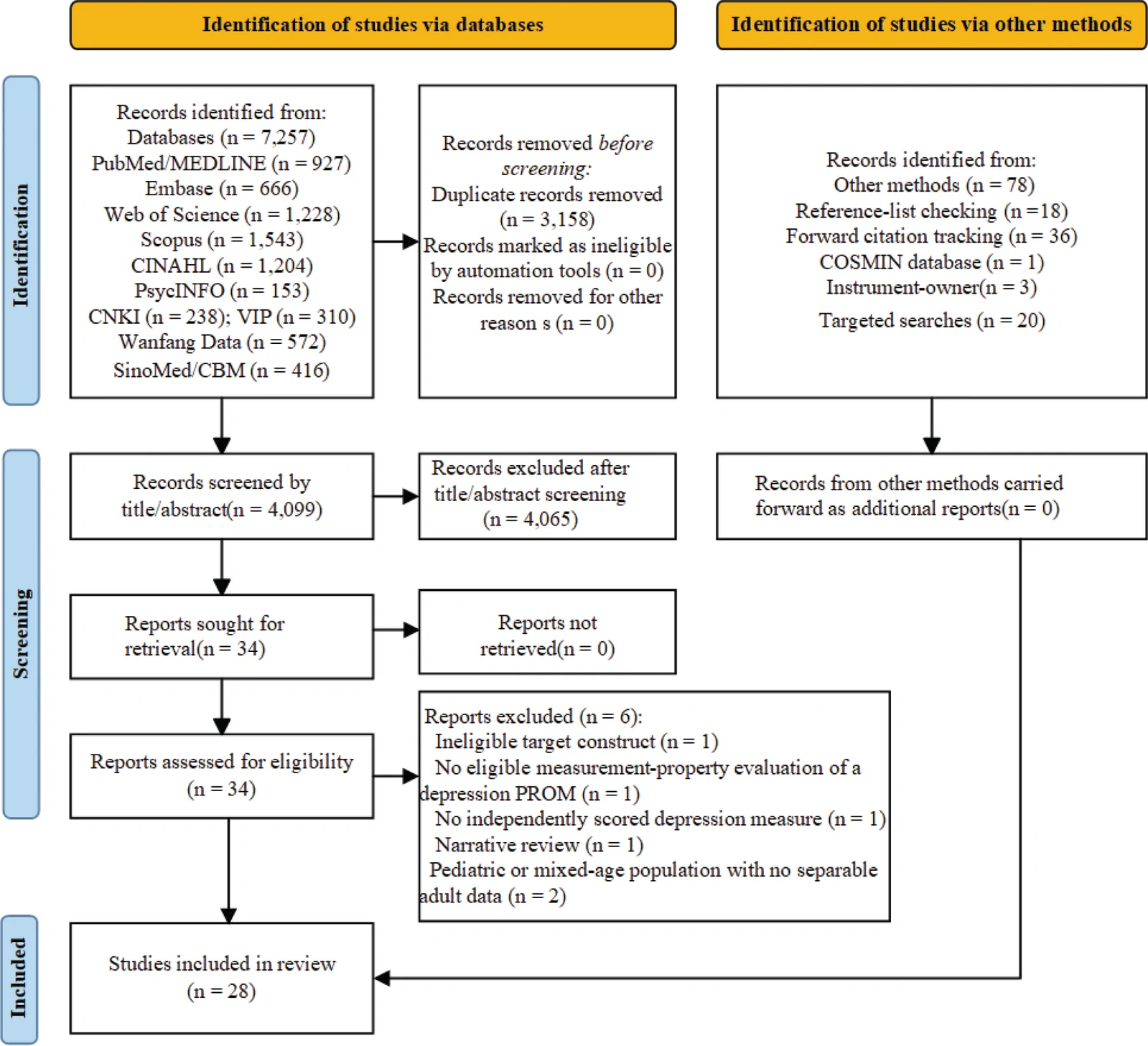
PRISMA-COSMIN flow diagram of report selection.

**Table I:** Characteristics of included patient-reported outcome measures

PROMfamily	PROM version/form (language)	Development year*	Construct measured	Recall period	Items/form	Response/scoring and score range
CES-D	CES-D-20 (English) (15,22,35) CES-D-10 (English) (22)	1977 (CES-D-20); 1994 (CES-D-10)	Depressive symptom frequency (both forms)	Past 7 days (both)	20-item total score 10-item short form	0–3 frequency scale; summed scores 0–60 (20-item) and 0–30 (10-item)
Zung SDS	Zung Self-Rating Depression Scale (English) (16,19)	1965	Frequency and severity of affective and somatic depressive symptoms	Current/recent symptoms; exact interval NR	20-item total score; affective and somatic component scores	1–4 frequency scale; raw sum 20–80; optional index 25–100
BSI	Brief Symptom Inventory – Depression subscale (English) (16,23,41,42) Brief Symptom Inventory SCI-specific Depression Research Factor (7-item English) (42)	1982 (BSI); 1996 (SCI-specific 7-item factor)	Depressive symptoms within psychological distress SCI-specific depressive distress during initial inpatient rehabilitation	Past 7 days (both)	6-item depression subscale (53-item BSI) 7-item SCI-specific depression research factor	0–4 severity scale; mean item score 0–4; BSI-D may also use a norm-based T-score 0–4 severity scale; mean of 7 items, 0–4
BDI	Beck Depression Inventory-I (English) (17)	1961	Depressive symptom severity across cognitive, affective, behavioural, interpersonal, and somatic domains	Past week	21-item total score	Four graded responses (0–3); summed score 0–63
Ilfeld PSI	Ilfeld Psychiatric Symptom Index - Depression subscale (English) (19)	1976 (validated form used here)	Depressive symptoms	NR	10-item depression subscale	Four frequency categories; weighted score 0–100
PHQ	Patient Health Questionnaire-9 (English) (18,20,24–30,34,35,37,38) Patient Health Questionnaire-9, revised 3-category scoring (English) (34) Patient Health Questionnaire-9, Arabic version (Lebanese Arabic) (36) Patient Health Questionnaire-2 (English) (18,37,38) PHQ-9-derived 3-item short form (English) (18)	2001 (PHQ-9); 2016 (revised 3-category scoring); 2016 (Lebanese Arabic translation); 2003 (PHQ-2); 2008 (SCI-derived 3-item form)	DSM-IV depressive symptoms DSM-IV depressive symptoms and severity DSM-IV depressive symptoms Anhedonia and depressed mood Anhedonia, depressed mood, and negative self-evaluation	Past 2 weeks Past 2 weeks Past 2 weeks Past 2 weeks Past 2 weeks	9-item total score; DSM-IV-based probable-MDD algorithm Revised 9-item PHQ-9 score 9-item total score 2-item total score (PHQ-9 items 1 and 2) 3-item score derived from PHQ-9 items 1, 2, and 6	0–3 frequency scale; 9-item sum 0–27; DSM-based algorithm also available Three-category recoding (0–2); 9-item sum 0–18 0–3 frequency scale; 9-item sum 0–27 0–3 frequency scale; 2-item sum 0–6 0–3 frequency scale; 3-item sum 0–9
HADS	Hospital Anxiety and Depression Scale – Depression subscale (English) (21) Hospital Anxiety and Depression Scale – Depression subscale (German) (31) Hospital Anxiety and Depression Scale – Depression subscale (Turkish) (32)	1983 (parent HADS; language-version development years NR)	Depressive symptoms with limited somatic content (all versions)	NR Past week Past week	7-item HADS-D subscale (all versions)	Item-specific 0–3 responses; 7-item sum 0–21 (all versions)
DASS	Depression Anxiety Stress Scales-21 – Depression subscale (English) (23)	1995	Depressive symptoms emphasizing affective/cognitive content and limiting somatic items	Past week	7-item DASS-21 Depression subscale	0–3 frequency scale; 7-item sum multiplied by 2; 0–42
OAHMQ	Older Adult Health and Mood Questionnaire (English) (28)	1995	Depressive symptoms with limited physical/vegetative content	NR	22-item total score	True/false responses; summed score 0–22
SCI-QOL	SCI-QOL Depression Item Bank (English) (33) SCI-QOL Depression CAT (English) (33) SCI-QOL Depression Short Form 10a (English) (33)	2015	SCI-specific depressive symptom severity (all forms)	Past 7 days (all forms)	28-item calibrated item bank 4–12 items under default CAT settings 10-item fixed short form	Five response options; graded-response IRT scoring; theta/PROMIS T-score; raw 28–140 if all items are administered Adaptive IRT T-score; 4–12 items, stopping at *SE* < 0.30 or 12 items Five response options; raw 10–50 converted to linked T-score 38.3–81.9
HSCL	Hopkins Symptom Checklist-20 Depression Scale, Version B (English) (34) Hopkins Symptom Checklist revised 12-item depression scale (English) (34)	1995 (HSCL-20B); 2016 (revised 12-item form)	Depressive symptom distress across affective, cognitive, somatic, and DSM-IV-related content Depressive symptom distress	Past week Past week	20-item HSCL depression score Reported 12-item revised HSCL score	0–4 severity scale; 20-item score; exact sum/mean algorithm NR (0–80 if summed) Responses recoded 0–2; reported 12-item score; retained-item algorithm unclear (theoretical 0–24)
PROMIS	PROMIS Depression Short Form 8b, version 1.0 (English) (35)	2011 (PROMIS Depression item bank; SF 8b v1.0 derived from bank)	Frequency and severity of depressive symptoms	Past 7 days	8-item short form	1–5 frequency scale; raw sum 8–40 converted to PROMIS T-score (observed 36–85)
SCL-90-R	SCL-90-R Cognitive/Affective Depression Research Subscale (English) (39) SCL-90-R Somatic Depression Research Subscale (English) (39)	1986 (research subscales)	Cognitive/affective depressive distress Somatic depressive distress	NR (both)	13-item cognitive-affective score 7-item somatic score	0–4 severity scale; mean of 13 items, 0–4 0–4 severity scale; mean of seven items, 0–4
IDD	Inventory to Diagnose Depression total severity score (English) (40) Inventory to Diagnose Depression Dysphoria factor score (English) (40)	1986 (IDD); 1992 (dysphoria factor)	DSM-III/DSM-III-R depressive symptoms and severity Core cognitive/affective dysphoria	NR (both)	22-item IDD total severity score 9-item dysphoria factor score	Severity score and DSM-based classification; detailed scoring and range NR Nine-item factor score; complete scoring algorithm and range NR

All fixed forms were self-report patient-reported outcome measures (PROMs); oral administration or physical assistance was permitted in some spinal cord injury (SCI) studies. The SCI-QOL Depression CAT used computer-adaptive administration. Unless stated otherwise, higher scores indicate greater depressive symptoms or distress.

* Development year denotes the year of the original instrument publication/manual or the first published derivation of the specific form evaluated, as identifiable from the included reports and their cited source material; translation-specific years are shown only when explicitly identifiable, and the parent-instrument year is otherwise reported. CAT: computer-adaptive test; IRT: item response theory; MDD: major depressive disorder; NR: not reported.

**Table II:** Characteristics of included reports

Report	Country and setting	Sample, *n*	Age (years); sex	SCI characteristics	Time since injury	Target PROM(s) and measurement properties
McColl & Skinner, 1992 (15)	Canada; 5 Ontario SCI centres; post-discharge home/telephone follow-up	T1 *n* = 138; T2 *n* = 128; complete T3 *n* = 120	Age groups: < 25 34%; 26–34 29%; 35–49 17%; 50–69 15%; ≥ 70 5%; 76.7% male	Traumatic 91.7%, progressive 8.3%; cervical 40.8%, thoracic 45.0%, lumbar 14.2%; complete 42.0%, incomplete 58.0%	Assessed 1, 4, and 12 months after rehabilitation discharge; exact time since injury NR	CES-D-20: SV, IC, HT
Tate et al., 1993 (16)	USA; 2 rehabilitation-centre outpatient databases; oral BSI/Zung administration	Main *n* = 162; classification subsample *n* = 30	Main: mean 33.6 (SD 9.9), 79.0% male; subsample: mean 31.3 (*SD* 12.4), 93.3% male	New traumatic SCI. Main outpatient sample: paraplegia 45%, quadriplegia 55% Inpatient validation sample: paraplegia 30%, quadriplegia 70%	Main outpatient sample: 6 months–5 years after injury Inpatient validation sample: initial rehabilitation; exact duration NR	Zung SDS and BSI-D: SV, IC, CRV, HT
Radnitz et al., 1997 (17)	USA; 3 VA SCI centres; male inpatients/outpatients	Recruited *n* = 138; analysed *n* = 124	Mean 48.8 (SD 13.7), 19–79; 100% male	Traumatic SCI; paraplegia 41.8%, quadriplegia 57.3%	Mean 18.9 years since injury	BDI-I: IC, CRV
Graves & Bombardier, 2008 (18)	USA; 16 Model SCI System centres; NSCISC records (Oct 2000–Apr 2003)	*n* = 3652	Mean 41.4 (SD 13.44), 18–90; 78.4% male	Traumatic SCI; injury level and completeness NR	Interviews 1–30 years after injury; mean 9.6 (SD 8.19) years. Analyses compared ≤ 2 vs > 2 years	PHQ-9: SV, MI PHQ-2: SV 3-item form: SV, MI, CRV
Campagnolo et al., 2002 (19)	USA; chronic SCI outpatients and able-bodied controls	SCI *n* = 39; controls *n* = 20	Mean age: tetraplegia 35 (SD 14), paraplegia 35 (12), controls 31 (10); sex NR	Complete or incomplete SCI; tetraplegia *n* = 20, paraplegia *n* = 19	Chronic SCI ≥ 3 months; mean 66 (SD 95.8) months for tetraplegia and 60 (SD 82.7) months for paraplegia	Zung SDS and Ilfeld PSI-D: HT
Bombardier et al., 2004 (20)	USA; 16 Model SCI System centres; standardized 1-year follow-up	*n* = 849	Mean at injury 36.9 (SD 15.0), 17–88; 76.0% male	Traumatic SCI; 45.5% paraplegia; 47.6% ASIA A (complete)	Standardised assessment at 1 year after injury	PHQ-9: IC, HT
Woolrich et al., 2006 (21)	UK; community postal survey through 3 spinal centres	Participants *n* = 985; HADS complete *n* = 963	Mean 48.1 (SD 12.7), 20–97; 81% male	Community SCI; paraplegia 65.2%, tetraplegia 34.8%; completeness NR	Mean 19.5 (SD 12.26) years since injury	HADS-D: SV, IC, CCV, HT
Miller et al., 2008 (22)	Canada; community sample; baseline and approximately 2-week retest	Recruited *n* = 55; retested *n* = 47	Mean 40.6 (SD 12.6); 64% male	Traumatic SCI; ASIA A 62%, B 38%	> 1 year since injury; mean 15.2 (SD 11.7) years, range 1–41 years	CES-D-20 and CES-D-10: IC, Rel, ME, HT
Mitchell et al., 2008 (23)	Australia; South Australian SCI Service (33 inpatients, 7 outpatients)	Approached *n* = 51; analysed *n* = 40	Mean 49.1 (*SD* 16.7), 19–82; 75% male	Paraplegia *n* = 26, tetraplegia *n* = 14; complete *n* = 20, incomplete *n* = 9, status unknown *n* = 11	Mean 113.9 (SD 150.3) months since injury; median 14, range 1–520 months Mixed inpatient and outpatient phases	BSI-D and DASS-21-D: CRV, HT
Richardson & Richards, 2008 (24)	USA; National SCI Database, 16 Model SCI Systems; 1-, 5-, 15-, and 25-year strata	*n* = 2,570 (682/517/653/718)	Stratum means 38.66–49.49; 77.7%–79.3% male	Traumatic SCI; paraplegia and tetraplegia, complete and incomplete injuries represented PI 14.9–19.6%, PC 26.1–34.2%, TI 22.1–31.5%, TC 22.7–27.3%	Post-injury strata: 1, 5, 15, and 25 years	PHQ-9: SV, IC, CCV, HT
Krause et al., 2008 (25)	USA; consecutive initial inpatient admissions at a southeastern Model SCI System	Consented *n* = 707; materials *n* = 584; analysed *n* = 568	Mean at SCI onset 32.4; 78% male	Traumatic SCI; cervical 51.1% Neurologic groups: no movement or sensation 29.3%, sensation only 28.2%, non-functional motor recovery 30.3%, functional motor recovery or ambulatory 12.0%	Initial inpatient rehabilitation; mean 50.2 (SD 27.1) days since injury, median 43 days	PHQ-9: SV, IC
Kalpakjian et al., 2009 (26)	USA; sex-matched NSCISC/Model SCI System follow-up sample	*n* = 1168 (584 women/584 men)	Mean: women 41.1 (SD 13.1), men 41.6 (13.2)	Traumatic SCI; matched by sex for injury level and completeness: paraplegia incomplete 18.5%, paraplegia complete 30.8%, tetraplegia incomplete 31.3%, tetraplegia complete 19.5%	Follow-up years: 1 (30.9%), 5 (22.1%), 10 (14.2%), 15 (11.8%), 20 (10.3%), and 25 (10.8%)	PHQ-9: SV, IC, CCV
Williams et al., 2009 (27)	USA; screening cohort for the multicentre venlafaxine XR trial (later PRISMS)	Total *n* = 202; EFA 91; CFA 111; Rasch 202	Mean 42.6 (SD 13.9), 18–80; 77% male	Injury mechanisms: gunshot 27%, road traffic 26%, falls 12%, other 35%; injury level and completeness NR	SCI ≥ 1 year; range 1–44 years, median 7 years	PHQ-9: SV, IC
Krause et al., 2009 (28)	USA; community follow-up from a southeastern specialty-hospital SCI cohort	Respondents *n* = 727; PHQ-9 690; OAHMQ 682	Mean 47.9; 70.2% male	Cervical injury 53.3% Neurologic status: no movement or sensation 41.8%, sensation only 20.6%, non-functional motor recovery 8.7%, ambulatory 21.2%	Community follow-up; mean 18.2 years since injury	PHQ-9 and OAHMQ: IC, HT
Krause et al., 2010 (29)	USA; longitudinal inpatient-rehabilitation cohort with 2 community follow-ups	Baseline *n* = 584; factor analyses 554/211/302	Survey means 32.4/36.9/35.2; 79.0%/74.5%/72.8% male	Traumatic SCI Cervical injury 51.0%, 47.4%, 48.0%; thoracic 42.2%, 44.0%, 43.9%; lumbar 6.7%, 8.1%, 7.8%; sacral 0.2%, 0.5%, 0.3% at baseline, first, and second follow-up, respectively	Mean 49.8 (SD 26.6), 498.4 (92.9), and 873.6 (152.3) days since injury at baseline, first, and second follow-up	PHQ-9: SV, CCV
Krause et al., 2011 (30)	USA; NSCISC/Model SCI System community follow-up records	Any follow-up *n* = 10978; complete PHQ-9 *n* = 7296	Mean 42.0 (SD 13.7); 79% male.	Cervical injury 52.2%; ASIA A 52.1%, B 12.9%, C 12.9%, D 22.1%	Community follow-up ≥ 1 year after SCI: year 1, 33.2%; 2–5 years, 16.3%; 6–10 years, 13.1%; > 10 years, 37.4%	PHQ-9: SV, CCV
Müller et al., 2012 (31)	Switzerland; community postal survey through Zurich, Basel, and Nottwil SCI centres	Responders *n* = 102; Rasch *n* = 100	Mean 56.5 (SD 16.7); 74.5% male	Mixed traumatic and non-traumatic SCI; paraplegia 61.8%, tetraplegia 38.2%; complete 26.5%, incomplete 73.5%	Mean 43.6 (SD 13.5) months since onset and 38.4 (SD 13.9) months since first rehabilitation discharge	German HADS-D: SV, IC, CCV
Paker et al., 2013 (32)	Türkiye; outpatient physical medicine and rehabilitation hospital	*n* = 175	Mean 35 (SD 13), 18–77; 81.7% male	Traumatic SCI Paraplegia 74.9%, tetraplegia 25.1%; AIS A 46.5%, B 16.0%, C 25.0%, D 12.5%	13 (SD 29) months in the abstract; 17 (SD 33) months, range 1–240, in Table 2	Turkish HADS-D: IC
Tulsky et al., 2015 (33)	USA; 5 SCI Model System centres and 1 VA centre; SCI-specific development programme	Item bank *n* = 716; crosswalk *n* = 465; CAT retest *n* = 245	Calibration sample: mean 43.0 (SD 15.3); 78% male	Traumatic SCI Calibration sample: paraplegia complete 25%, paraplegia incomplete 20%, tetraplegia complete 22%, tetraplegia incomplete 32%	Mean 7.1 (SD 10.0) years since injury; < 1 year 27%, 1–3 years 26%, > 3 years 47%. DIF compared < 1 vs ≥ 1 year	Item Bank: Dev, CV, SV, IC, MI, HT CAT: Rel SF 10a: derived; no independent evaluation
Williams et al., 2016 (34)	USA; multicentre PRISMS randomised placebo-controlled venlafaxine XR trial	Participants *n* = 133; PHQ-9 observations 884; HSCL observations 885	Mean 40 (SD 11); 74% male	Traumatic SCI with MDD; 53% complete injuries Aetiology: vehicular 36%, violent 32%, falls 15%, other 17%, unknown < 1%; injury level NR	Mean 11 (SD 11) years since injury	Original PHQ-9: SV, IC Revised PHQ-9: SV, IC, CRV, HT, Resp HSCL-20B: SV, IC Revised HSCL-12: SV, IC, CRV, HT, Resp
Cook et al., 2017 (35)	USA; secondary DIF analyses of SCI/reference pairs for CES-D, PHQ-9, and PROMIS-D 8b	SCI/reference: CES-D 238/726; PHQ-9 3694/3000; PROMIS-D 292/761	SCI: mean 40.4–49.2; 61.3%–76.8% male Reference: mean 46.0–51.4; 33.7%–48.5% male	Secondary analysis of 3 SCI cohorts; study-specific injury level, completeness, and aetiology NR	Study-specific time since injury NR	CES-D-20, PHQ-9, PROMIS-D 8b: CCV/MI
Summaka et al., 2019 (36)	Lebanon; 3 rehabilitation centres; standardized specialist interviews with assistance as needed	Main *n* = 51; pilot 15; retest 20	Mean 37.2 (SD 12.6), 20–60; 100% male	Traumatic or non-traumatic SCI; paraplegia 72.5%, tetraplegia 27.5%. Cause: war or explosions 52.9%, motor-vehicle accidents 13.7%, falls 13.7%, disease 13.7%, other 5.9%	Date of injury was collected; summary time since injury NR	Arabic PHQ-9: CV (comprehensibility only), SV, IC, Rel, CRV, HT
Bombardier et al., 2012 (37)	USA; 4 inpatient units at 3 Model SCI System sites; blinded SCID comparison	Enrolled *n* = 205; SCID 173; paired ≤ 7 days 142	Paired sample: mean 42.2 (SD 16.6), 18–88; 78.2% male	Acute traumatic SCI; cervical 66.9%, thoracic 22.5%, lumbar 7.7%, sacral 2.8%	Initial inpatient rehabilitation; PHQ-9 administered mean 54.0 (SD 40.4) days after SCI, median 44.5, range 4–279 days	PHQ-9: CRV, HT PHQ-2: CRV
Poritz et al., 2018 (38)	USA; VA annual SCI/D evaluation; PHQ-2 and PHQ-9 administered independently	Assigned *n* = 144; completed both *n* = 116	Mean 56.0 (SD 12.4); 96.6% male	Community-residing spinal cord injury or dysfunction; traumatic 81.0%, non-traumatic 19.0%; tetraplegia AIS A–C 24.2%, paraplegia AIS A–C 31.7%, AIS D 38.8%	Mean 18.1 (SD 13.3) years since spinal cord injury or dysfunction	PHQ-9: IC PHQ-2: IC, CRV
Buckelew et al., 1988 (39)	USA; University of Missouri SCI rehabilitation; SCL-90-R at initial evaluation	SCI *n* = 52; chronic pain 78; controls 145	SCI mean 30; 44 men/8 women	SCI rehabilitation sample; injury level, completeness, and aetiology NR	Mean injury duration 1.5 years	Both SCL-90-R research subscales: IC, HT
Frank et al., 1992 (40)	USA; medical-school and associated VA SCI rehabilitation facilities	SCI *n* = 134; age *n* = 132; 4-group ANOVA implied *n* = 456 (*df* = 3452)	SCI mean 39.8 (*SD* 13.2), 18–66; 131 men/3 women	SCI rehabilitation sample; injury level, completeness, and aetiology NR	NR	IDD total and Dysphoria factor: SV, MI, HT
Tate et al., 1990 (41)	USA; University of Michigan SCI rehabilitation (45 inpatients, 34 outpatients)	BSI *n* = 79; paired subset *n* = 20	Mean 33, 18–70; 73.4% male	Traumatic SCI; 45 inpatients and 34 outpatients; injury level and completeness NR	Mean 7 months since injury, range 1–12 months	BSI-D: HT
Heinrich & Tate, 1996 (42)	USA; initial inpatient rehabilitation; oral BSI by psychologist/social worker	Factor analysis *n* = 215; 1-year follow-up *n* = 204	Mean 35.2 (*SD* 14.3), 18–70; 79% male	Traumatic SCI; quadriplegia 52%; other injury level and completeness detail NR	Initial inpatient rehabilitation; exact time since injury NR One-year post-discharge outcome follow-up was available	BSI-D: SV, HT 7-item SCI factor: SV, IC, HT

CV: content validity; SV: structural validity; IC: internal consistency; CCV: cross-cultural validity; MI: measurement invariance; Rel: reliability; ME: measurement error; CRV: criterion validity; HT: hypotheses testing for construct validity; Resp: responsiveness; Dev: PROM development; VA: Veterans Affairs. Where several PROMs share 1 property list, it applies to each unless otherwise specified. Sample sizes are property-specific; nested, overlapping, or repeated samples were not treated as independent precision contributions. SCI characteristics and time since injury are reported as presented in the source studies; NR indicates information not reported or not extractable at study level.

The PHQ family had the largest evidence base and was evaluated as a target instrument in 14 reports. Thirteen reports evaluated the English PHQ-9 and 1 evaluated the Lebanese Arabic PHQ-9; 3 also evaluated the PHQ-2, 1 derived a 3-item PHQ-9 form, and 1 evaluated a *post hoc* 3-category PHQ-9 scoring form. These categories overlap because several reports examined more than 1 PHQ form (18, 20, 24–30, 34–38). HADS-D and CES-D were each evaluated in 3 reports (15, 21, 22, 31, 32, 35). Within the SCI-QOL programme, the 28-item Depression Item Bank and CAT were psychometrically evaluated, whereas Short Form 10a was derived and scored but not independently validated (33). Other reports evaluated BSI depression scores, Zung SDS, BDI-I, Ilfeld PSI Depression, DASS-21 Depression, OAHMQ, PROMIS Depression Short Form 8b, HSCL forms, IDD scores, and experimental SCL-90-R depression subscales.

Internal consistency was evaluated in 19 reports, hypotheses testing for construct validity in 17, structural validity in 16, cross-cultural validity or measurement invariance in 10, criterion validity in 8, and reliability in 3. Measurement error and responsiveness were each evaluated in only 1 report. The distribution of evidence across instrument families and measurement properties is shown in Fig. 2. Formal SCI-specific PROM-development evidence was identified only for SCI-QOL Depression; study-based content-validity evidence for other PROMs was limited to pilot comprehensibility testing of the Lebanese Arabic PHQ-9 (33, 36). PROM-level interpretability, feasibility, and accessibility are summarized in Table SIII.

**Fig. 2:**
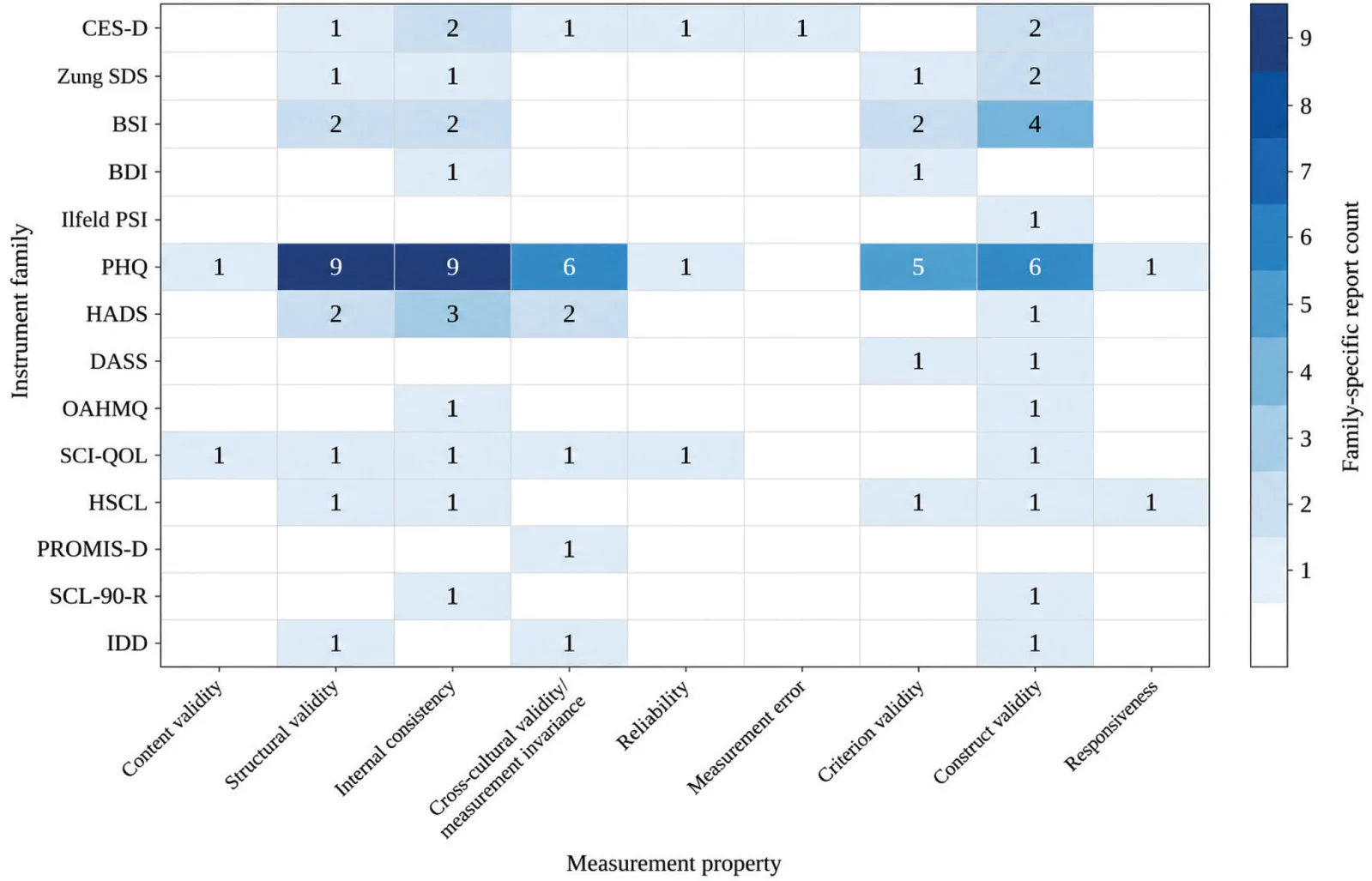
Evidence coverage across instrument families.

### Risk of bias

Across 130 evaluable individual analysis-result units, risk of bias was rated very good for 29, adequate for 18, doubtful for 40, and inadequate for 43; 3 additional checklist components were rated not applicable and were not included in the denominator of 130 evaluable analysis-result units. Structural-validity studies were commonly limited by exploratory analyses without confirmatory testing, incomplete reporting of model assumptions or fit, and inadequate effective sample sizes for the number of items. Internal-consistency coefficients were generally calculated appropriately, but they could not be interpreted when sufficient unidimensionality had not been established.

Diagnostic validation studies using blinded structured interviews generally had the strongest methods, although the number of participants with major depressive disorder was often small. Reliability studies were limited by uncertain clinical stability, incompletely specified intraclass correlation models, or small retest samples. Common limitations in hypotheses testing included unmatched comparison groups, comparators with incompletely established properties, unreported analytic denominators, and multiple testing without explicit adjustment. Consensus ratings are reported in Table SIV, and the corresponding measurement-property results and COSMIN ratings are reported in Table SV.

### Content validity and PROM development

The SCI-QOL Depression Item Bank was the only PROM supported by SCI-specific development evidence (33). Relevance and comprehensiveness were sufficient with low certainty. Although study-based comprehensibility was indeterminate, integration with the consensus reviewer assessment yielded a sufficient rating with very-low certainty.

The Lebanese Arabic PHQ-9 was pilot-tested in 15 adults with SCI, with no reported comprehension problems and a completion time of approximately 4–6 min (36). Integrated ratings were sufficient for relevance and comprehensiveness with very-low certainty and for comprehensibility with low certainty.

No eligible form-specific study-based content-validity evidence was identified for the remaining PROMs; evidence for the SCI-QOL Depression CAT and Short Form 10a was indirect from item-bank development. Most integrated conclusions therefore relied on consensus reviewer assessment and were of very-low certainty. Brief affective or cognitive forms were generally relevant and comprehensible but often insufficiently comprehensive; ratings were indeterminate when authoritative item sets or language-specific wording were unavailable. Detailed ratings are presented in Table SVII.

### Structural validity and internal consistency

The English PHQ-9 had the largest structural-validity evidence base. Across 10 principal analysis units, 8 were insufficient under current COSMIN criteria. Several analyses suggested 1- or 2-factor representations, but most did not meet prespecified fit thresholds, and Rasch analyses identified disordered response categories or item misfit. The few apparently supportive analyses did not establish a consistently acceptable basis for interpreting the conventional 9-item total as a unidimensional score. Total-score structural validity was therefore summarized as insufficient rather than inconsistent, with high-certainty evidence. Although reported Cronbach’s alpha values were generally at least 0.80, internal consistency remained indeterminate because sufficient unidimensionality had not been established (18, 24–30, 34).

The 28-item SCI-QOL Depression Item Bank showed sufficient structural validity with moderate certainty: confirmatory analysis yielded CFI = 0.968 and RMSEA = 0.066, and item-response analyses supported calibration of the bank. Cronbach’s alpha = 0.964 and marginal reliability = 0.950 supported sufficient internal consistency with moderate certainty (33). Short Form 10a was derived from the calibrated bank and accompanied by a raw-score-to-*t*-score conversion table, but it was not independently evaluated for structural validity, internal consistency, reliability, or responsiveness.

For the English HADS-D, a large UK study supported the intended depression factor and reported alpha = 0.79; structural validity and internal consistency were both sufficient with moderate certainty (21). The German HADS-D Rasch analysis remained indeterminate despite generally acceptable residual dimensionality, and the Turkish version contributed only an internal-consistency estimate without structural validation (31, 32).

Structural validity was insufficient for the CES-D-20, Zung Self-Rating Depression Scale, standard BSI Depression subscale, IDD total score, and both evaluated HSCL forms. The Lebanese Arabic PHQ-9, IDD dysphoria score, and SCI-specific 7-item BSI Depression factor remained indeterminate. Several measures reported apparently acceptable internal-consistency coefficients, but these results remained indeterminate because sufficient structural validity had not been demonstrated (15, 16, 22, 34, 36, 39, 40, 42).

### Cross-cultural validity and measurement invariance

Ten reports addressed cross-group or cross-language functioning, but most results were indeterminate because recognized multi-group confirmatory factor analysis or formal item-level differential item functioning criteria were not used or were incompletely reported. For the SCI-QOL Depression Item Bank, 10 items showed possible differential item functioning with McFadden pseudo-*R*-squared values above 0.02; measurement invariance was therefore insufficient with low certainty (33).

Cook and colleagues found no items exceeding their prespecified standard differential-item-functioning thresholds for the CES-D, PHQ-9, or PROMIS Depression Short Form 8b when comparing SCI and non-SCI samples, but exact item-level effect sizes required to verify the COSMIN threshold were not reported; the results remained indeterminate (35). Subgroup information-function comparisons for the PHQ-9 and its 3-item form suggested limited differences by sex, race, education, or time since injury, but did not constitute formal invariance testing (18). Evidence for the German HADS-D, Lebanese Arabic PHQ-9, and IDD forms was also incomplete or methodologically indirect (31, 36, 40).

### Reliability and measurement error

Reliability was evaluated for only a small subset of PROMs. The CES-D-20 and CES-D-10 showed sufficient 2-week test–retest reliability, with ICC(1,1) = 0.87 (95% CI 0.79–0.93) and 0.85 (95% CI 0.75–0.92), respectively, although certainty was very low (22). The SCI-QOL Depression CAT showed sufficient 1- to 2-week reliability in an independent sample of 245 participants (ICC(2,1) = 0.80, 95% CI 0.75–0.84; low certainty) (33). The Lebanese Arabic PHQ-9 had an average-measure ICC of 0.886 (95% CI 0.711–0.955) in 20 participants, but incomplete reporting of stability and the ICC specification reduced certainty to low (36).

Measurement error was evaluated in only 1 report and remained indeterminate because numerical limits of agreement, smallest detectable change, and an anchor-based minimal important change were not established (22). No PROM had sufficient evidence for an SCI-specific minimal important change. Consequently, an observed repeated-score difference cannot yet be translated confidently into a clinically important individual change.

### Criterion validity and construct validity

The strongest supplementary diagnostic evidence concerned the English PHQ-9. Against a blinded Structured Clinical Interview for DSM-IV, the area under the curve was 0.921. A cut-off of at least 11 yielded sensitivity of 1.00 and specificity of 0.84, whereas a cut-off of at least 10 yielded sensitivity of 1.00 and specificity of 0.80. Criterion validity was sufficient with moderate certainty, although only 14 participants had interview-defined major depressive disorder (37). These estimates were treated as descriptive case-finding evidence rather than as a stand-alone basis for recommending a screening threshold.

The PHQ-2 did not achieve acceptable sensitivity and specificity simultaneously against the same interview. At a cut-off of at least 1, sensitivity was 1.00 and specificity was 0.48. Because an eligible area under the curve or correlation with the diagnostic criterion was not reported, criterion validity was rated indeterminate and was not graded. The result may justify further study of the PHQ-2 as a first-stage pre-screen, but it does not support a formal recommendation (37). Findings for BDI-I, Zung, DASS-21 Depression, and the Lebanese Arabic PHQ-9 were indeterminate because eligible area-under-the-curve or criterion-correlation evidence was absent, accuracy was inadequate, or the comparator was not an accepted diagnostic gold standard (16, 17, 23, 36).

Construct validity was most consistently supported for the English PHQ-9 across associations with depressive-symptom comparators, general health, life satisfaction, quality of life, and functional difficulty; the summarized result was sufficient with low certainty (20, 24, 28, 29, 37). English HADS-D showed sufficient construct validity with moderate certainty, including a correlation of *r* = -0.660 with life satisfaction (21). SCI-QOL Depression showed sufficient construct validity with very-low certainty because the number and quality of available hypotheses were limited (33).

Construct-validity evidence was sufficient but of very-low certainty for CES-D-20 (15, 22), BSI Depression (16, 23, 41, 42), DASS-21 Depression (23), Ilfeld PSI Depression (19), OAHMQ (28), the revised 3-category PHQ-9 and revised HSCL form (34), the SCL-90-R cognitive/affective subscale (39), the IDD dysphoria score (40), and the SCI-specific BSI Depression factor (42). Results were insufficient for the SCL-90-R somatic depression subscale and the IDD total severity score. Several older known-groups studies were downgraded because comparison groups were unmatched or clinically dissimilar; pooled correlations that could not be separated for SCI were excluded from the SCI-specific synthesis.

### Responsiveness

Responsiveness was examined in only 1 trial of 133 adults with traumatic SCI who met diagnostic criteria for major depressive disorder. A revised 3-category PHQ-9 score and a revised HSCL form met the prespecified responsiveness criterion, but certainty was very low because both scoring forms were developed and evaluated in the same dataset and the revised HSCL item composition and scoring algorithm could not be fully reconstructed (34). No responsiveness evidence was available for SCI-QOL Depression, the conventional PHQ-9 score, HADS-D, CES-D, or most other commonly used PROMs. Summarized ratings and certainty levels are presented in Table III; complete downgrading decisions for graded results are provided in Table SVIA.

**Table III:** Summary of measurement-property ratings and certainty of evidence

PROM/version	CV	SV	IC	CCV/MI	Rel	ME	CRV	HT	Resp
CES-D-20 (English)	R ± (VL) C + (VL) U + (VL)	- (*M*)	?	?	+ (VL)	?	—	+ (VL)	—
Zung SDS (English)	R - (VL) C + (VL) U + (VL)	- (L)	?	—	—	—	?	+ (L)	—
BSI-D (English)	R + (VL) C - (VL) U + (VL)	- (VL)	?	—	—	—	?	+ (VL)	—
BDI-I (English)	R ± (VL) C + (VL) U + (VL)	—	?	—	—	—	?	—	—
Ilfeld PSI-D (English)	R ? C ?U ?	—	—	—	—	—	—	+ (VL)	—
PHQ-9 (English)	R ± (VL) C + (VL) U + (VL)	- (H)	?	?	—	—	+ (*M*)	+ (L)	—
HADS-D (English)	R + (VL) C - (VL) U + (VL)	+ (*M*)	+ (M)	?	—	—	—	+ (*M*)	—
CES-D-10 (English)	R ± (VL) C - (VL) U + (VL)	—	?	—	+ (VL)	?	—	?	—
DASS-21-D (English)	R + (VL) C - (VL) U + (VL)	—	—	—	—	—	?	+ (VL)	—
OAHMQ (English)	R ? C ?U ?	—	?	—	—	—	—	+ (VL)	—
HADS-D (German)	R + (VL) C - (VL) U ?	?	?	?	—	—	—	—	—
HADS-D (Turkish)	R + (VL) C - (VL) U ?	—	?	—	—	—	—	—	—
SCI-QOL Depression Item Bank (English)	R + (L) C + (L) U + (VL)	+ (*M*)	+ (M)	- (L)	—	—	—	+ (VL)	—
SCI-QOL Depression CAT (English)	R + (VL) C ? U + (VL)	—	—	—	+ (L)	—	—	—	—
SCI-QOL Depression SF 10a (English)	R + (VL) C ? U + (VL)	—	—	—	—	—	—	—	—
PHQ-9, revised 3-category scoring (English)	R ± (VL) C + (VL) U + (VL)	?	?	—	—	—	+ (L)	+ (VL)	+ (VL)
HSCL-20 Depression, Version B (English)	R ± (VL) C + (VL) U + (VL)	- (VL)	?	—	—	—	—	—	—
HSCL revised 12-item depression scale (English)	R ? C ? U ?	- (VL)	?	—	—	—	+ (VL)	+ (VL)	+ (VL)
PROMIS Depression SF 8b v1.0 (English)	R + (VL) C - (VL) U + (VL)	—	—	?	—	—	—	—	—
PHQ-9 (Lebanese Arabic)	R + (VL) C + (VL) U + (L)	?	?	?	+ (L)	—	?	+ (L)	—
PHQ-2 (English)	R + (VL) C - (VL) U + (VL)	?	?	—	—	—	?	—	—
PHQ-9-derived 3-item SF (English)	R + (VL) C - (VL) U + (VL)	?	—	?	—	—	?	—	—
SCL-90-R Cognitive/Affective Depression subscale (English)	R + (VL) C - (VL) U + (VL)	—	?	—	—	—	—	+ (VL)	—
SCL-90-R Somatic Depression subscale (English)	R - (VL) C - (VL) U + (VL)	—	?	—	—	—	—	- (VL)	—
IDD total severity score (English)	R ± (VL) C + (VL) U ?	- (*M*)	—	?	—	—	—	- (VL)	—
IDD Dysphoria factor score (English)	R ± (VL) C - (VL) U ?	?	—	?	—	—	—	+ (VL)	—
BSI SCI-specific Depression Research Factor, 7-item (English)	R + (VL) C - (VL) U + (VL)	?	?	—	—	—	—	+ (VL)	—

Ratings: +, sufficient; −, insufficient; ±, inconsistent or mixed; ?, indeterminate; —, no eligible empirical study result was identified for that PROM version/form and measurement property. Certainty: H, high; M, moderate; L, low; VL, very low; indeterminate results were not graded. Internal consistency was indeterminate without sufficient structural validity. Abbreviations are defined in Tables I and II; within CV, R denotes relevance, C comprehensiveness, and U comprehensibility. Content-validity ratings integrate eligible study-based evidence and the final consensus assessments of 2 independent reviewers. Conclusions based solely on reviewer assessment were assigned very-low certainty. Detailed criterion-level ratings, integrated evidence bases, and rationales are presented in Table SVII; complete certainty assessments for graded measurement-property results and documentation of indeterminate or unavailable results are presented in Tables SVIA and SVIB, respectively.

### Interpretability, feasibility, and accessibility

Interpretability, feasibility, and accessibility varied across PROMs. The PHQ-9 and PHQ-2 contain 9 and 2 items, respectively, and oral administration was used when physical limitations prevented written completion. In the available SCI diagnostic study, PHQ-9 cut-offs of 10 and 11 and a PHQ-2 cut-off of 1 were evaluated; these thresholds are reported descriptively and are not recommendations (37). The Lebanese Arabic PHQ-9 required approximately 4–6 min in pilot testing (36). SCI-QOL Depression supported calibrated *T*-score reporting and flexible administration through an item bank, CAT, and fixed short form; however, the CAT required computerized administration and calibrated scoring infrastructure, and Short Form 10a lacked independent validation (33). Administration time, missing-item rules, floor and ceiling effects, reference values, and change-score interpretation were incompletely reported across PROMs, and no credible SCI-specific minimal important change was identified. Current access conditions also varied: some PROMs were available for specified clinical or research use without licensing fees, whereas others required licensing, purchase, registration, or platform-specific access; for several legacy or research-derived forms, current terms could not be established from authoritative sources. Details are provided in Table SIII.

### Purpose-specific evidence interpretation

No PROM met the evidence requirements for a formal recommendation for routine use across the intended purposes (Table IV). The SCI-QOL Depression Item Bank had the strongest SCI-specific development and evidence on internal structure; however, comprehensibility was supported only by very-low-certainty reviewer assessment, measurement invariance was insufficient, and longitudinal evidence was absent. The CAT had sufficient test–retest reliability but lacked independent evaluation of most CAT-specific properties, and Short Form 10a had no independent measurement-property evaluation. These forms are therefore candidates for further validation rather than recommended instruments.

**Table IV:** Formal categories and purpose-specific evidence interpretation

Intended use	PROM or form	Formal COSMIN category	Purpose-specific evidence interpretation and caveat
SCI-specific cross-sectional severity assessment	SCI-QOL Depression Item Bank	No recommendation	Strongest SCI-specific development and internal-structure evidence; relevance and comprehensiveness were sufficient with low certainty, and comprehensibility with very-low certainty. Measurement invariance was insufficient, while measurement error, MIC, and responsiveness were not established
Computer-adaptive severity assessment	SCI-QOL Depression CAT	No recommendation	Test–retest reliability was sufficient in one sample. Computerized calibrated scoring is required, and most CAT-specific measurement properties were not independently evaluated
Fixed short-form assessment	SCI-QOL Depression Short Form 10a	No recommendation	Derived from the calibrated item bank, but no independent measurement-property evaluation was available
Case-finding for probable MDD	PHQ-9 (English)	No recommendation; diagnostic evidence supplementary	AUC=0.921; cut-off ≥ 11 yielded sensitivity 1.00 and specificity 0.84 in 1 study with 14 SCID-defined MDD cases. Thresholds are descriptive; positive results require clinical assessment
Unidimensional continuous severity interpretation	PHQ-9 (English)	Recommended not to use for unidimensional total-score interpretation	High-certainty evidence showed insufficient structural validity for interpreting the conventional total as a clearly unidimensional severity measure. This does not preclude item-level review or use as 1 component of clinical assessment
Very brief first-stage pre-screen	PHQ-2 (English)	No recommendation; diagnostic evidence supplementary	At cut-off ≥ 1, sensitivity was 1.00 and specificity 0.48; no eligible AUC or criterion correlation was reported. Evidence is insufficient for recommendation
Assessment with limited somatic content	HADS-D (English)	No recommendation	Structural validity, internal consistency, and construct validity were sufficient with moderate certainty in 1 large UK study Reviewer appraisal indicated sufficient relevance and comprehensibility but insufficient comprehensiveness, all with very-low certainty; no eligible SCI-specific content-validity study was identified. Diagnostic accuracy and longitudinal measurement properties remain unestablished
Arabic-language assessment	PHQ-9 (Lebanese Arabic)	No recommendation	Comprehensibility, reliability, and construct validity had low-certainty support; structural and criterion validity were indeterminate. Evidence came from 51 men
General depressive-symptom assessment	CES-D-20 (English)	No recommendation	Reliability and construct validity were sufficient with very-low certainty, but structural validity was insufficient with moderate certainty. Internal consistency and measurement error were indeterminate; MIC and responsiveness were unavailable
Monitoring change during treatment	Revised 3-category PHQ-9; revised HSCL form	No recommendation; research use only	Criterion validity, construct validity, and responsiveness were sufficient with low or very-low certainty. Both forms were developed and evaluated post hoc in the same trial, and the revised HSCL scoring could not be fully reproduced
Other generic, translated, or research-derived PROMs	CES-D-10, Zung SDS, BSI-D, BDI-I, Ilfeld PSI-D, DASS-21-D, OAHMQ, German/Turkish HADS-D, original HSCL-20 Version B, PHQ-9-derived 3-item form, PROMIS-D SF8b, SCL-90-R research subscales, IDD forms, and the SCI-specific BSI factor	No recommendation	Only isolated properties were sufficient; remaining evidence was absent, indeterminate, insufficient, or of low/very-low certainty (Table III)

Formal COSMIN categories are “recommended for use,” “recommended not to use,” or “no recommendation.” Diagnostic screening is outside the primary evaluative-PROM focus; diagnostic indices are supplementary and do not replace clinical assessment. The “recommended not to use” category for the English PHQ-9 is restricted to interpreting the conventional total score as a clearly unidimensional continuous severity measure and does not prohibit item-level review or use within a broader clinical assessment.

Diagnostic screening evidence was interpreted separately from evaluative measurement. The English PHQ-9 had the clearest available case-finding evidence, but it came from 1 study with only 14 interview-defined major depressive disorder cases and did not justify a formal diagnostic-screening recommendation. High-certainty evidence of insufficient structural validity supported recommending against interpreting the conventional PHQ-9 total as a clearly unidimensional continuous severity measure. The PHQ-2, English HADS-D, Lebanese Arabic PHQ-9, CES-D-20, revised PHQ-9, revised HSCL, and the remaining PROMs all remained in the no-recommendation category; purpose-specific caveats are summarized in Table IV.

## DISCUSSION

This review identified a broad but fragmented evidence base: 28 reports evaluated 27 separately scored PROM versions, administration forms, or depression subscales, yet no PROM had a sufficiently complete evidence profile across the properties relevant to screening, continuous severity assessment, and measurement of change. The principal finding is therefore not the identification of a universally preferred instrument, but the need for purpose-specific interpretation. The SCI-QOL Depression Item Bank had the strongest SCI-specific development and internal-structure evidence, whereas the English PHQ-9 had the clearest supplementary case-finding evidence. Neither evidence profile supported an unconditional recommendation.

The SCI-QOL Depression Item Bank was the only PROM supported by an SCI-specific development programme and had integrated sufficient ratings for relevance and comprehensiveness with low certainty and for comprehensibility with very-low certainty. The distinction between study-based and integrated comprehensibility is important: qualitative testing was insufficiently reported in the development study, whereas the review-team assessment supported the clarity of the available English items and response options. Nevertheless, measurement invariance was insufficient, and measurement error, minimal important change, and responsiveness were not established. Moreover, item-bank evidence cannot be assumed to apply fully to the CAT or Short Form 10a, for which form-level comprehensiveness remained indeterminate. These limitations preclude confident interpretation of individual change and support further validation rather than routine endorsement.

The English PHQ-9 serves a different purpose. Reviewer-based content appraisal indicated sufficient comprehensiveness and comprehensibility but mixed relevance, reflecting uncertainty in attributing sleep, energy, appetite, concentration, and psychomotor symptoms specifically to depression in adults with SCI; these conclusions were of very-low certainty. One diagnostic study suggested possible value for pragmatic case finding, provided that positive results lead to clinical assessment. However, the small number of interview-defined cases and the absence of a dedicated diagnostic test accuracy review preclude formal endorsement of a specific threshold. High-certainty evidence of insufficient structural validity does not negate potential case-finding performance, but it limits interpretation of the conventional total score as a clearly unidimensional continuous severity measure. The coexistence of 1- and 2-factor solutions, together with Rasch evidence of disordered response categories or item misfit, further weakens the basis for assuming that the conventional PHQ-9 total score reflects a single underlying severity dimension in SCI. Clinicians should therefore consider item-level responses, suicidal ideation, symptom timing, medical context, and symptom trajectories rather than relying exclusively on the total score.

English HADS-D may be considered when minimizing somatic-content overlap as a priority because structural validity, internal consistency, and construct validity were sufficient with moderate certainty. Its reviewer-based content appraisal indicated sufficient relevance and comprehensibility but insufficient comprehensiveness, all with very-low certainty; no eligible SCI-specific content-validity study was identified. Diagnostic accuracy, reliability, measurement error, minimal important change, and responsiveness also remain unestablished. HADS-D should therefore be viewed as a candidate for further validation rather than a formally recommended alternative.

The review also illustrates why Cronbach’s alpha should not be treated as a stand-alone validation result. Several PROMs reported alpha values above 0.80 but lacked sufficient evidence that their total score or subscale was unidimensional. Under COSMIN, internal consistency is interpretable only after sufficient structural validity has been established. The distinction between diagnostic accuracy and evaluative measurement was equally important: sensitivity and specificity alone did not satisfy the review’s criterion-validity rule when an eligible area under the curve or correlation with a diagnostic reference standard was unavailable.

### Comparison with previous reviews

Earlier SCI reviews identified PHQ-9, HADS-D, CES-D, and selected brief measures as useful or promising, largely on the basis of internal consistency, correlations, and screening performance (8–10). The present review extends that work by applying the current COSMIN Risk of Bias checklist, criteria for good measurement properties, and modified GRADE approach; separating language versions, short forms, item banks, CATs, and independently scored research forms; distinguishing supplementary diagnostic screening evidence from continuous severity measurement and detection of change; and accounting for possible cohort overlap. This approach produces more conservative conclusions and separates formal COSMIN categories from pragmatic considerations.

### Implications for practice and research

For depression case-finding, the available English PHQ-9 evidence may help identify adults who warrant further assessment, but it is based on 1 study and should not be interpreted as a definitive threshold recommendation. Reported cut-offs of 10 and 11 are descriptive. A positive score is not a diagnosis, and any positive or clinically concerning response, particularly suicidal ideation, requires timely clinical review. The PHQ-2 may reduce initial burden, but low specificity at a cut-off of 1 and incomplete diagnostic evidence prevent a recommendation for routine pre-screening.

For cross-sectional severity assessment and research, SCI-QOL Depression is the most developed SCI-specific candidate, particularly when calibrated administration is available. The fixed Short Form 10a remains a practical candidate rather than a validated substitute for the item bank or CAT. No PROM can yet be recommended confidently for detecting clinically important change across rehabilitation, treatment, and long-term follow-up. Repeated scores should therefore be interpreted alongside clinical interviews, treatment context, functional recovery, and patient-reported global change. Practical PROM selection should also consider access conditions, as licensing, registration, fees, and platform requirements differ across instruments and may affect routine implementation (see Table SIII).

Future research should begin with content-validity studies in diverse SCI populations across different post-injury stages, including women, older adults, people with non-traumatic SCI, individuals with different neurological levels and completeness, and non-English-speaking or underrepresented cultural groups. Cognitive interviews with people with SCI should examine whether affective, cognitive, somatic, and suicidal-ideation items are understood as intended and whether relevant SCI-specific experiences are missing. Instructions, response options, recall periods, and administration adaptations for upper-limb impairment should also be evaluated explicitly.

Structural studies require adequately powered confirmatory or item-response analyses with complete reporting of model assumptions, local dependence, monotonicity, item fit, dimensionality, and prespecified fit criteria. New short forms or rescoring algorithms should be derived and validated in independent samples. Cross-cultural studies should use multi-group confirmatory factor analysis or formal differential-item-functioning methods and report item-level effect sizes that can be judged against COSMIN thresholds.

Longitudinal validation is the most urgent gap. Reliability studies should define a clinically stable interval and report an appropriate single-measure ICC with its model and confidence interval. Measurement-error studies should estimate the standard error of measurement, smallest detectable change, and limits of agreement. Responsiveness should be tested against prespecified hypotheses and credible anchors, followed by anchor-based estimation of minimal important change. Reports should identify cohort overlap and provide property-specific analytic denominators to prevent inadvertent double counting.

### Strengths and limitations

Strengths of this review include comprehensive searches across international and Chinese databases using English and Chinese search terms, application of the current COSMIN methodology, independent duplicate assessment, version-specific synthesis, explicit management of overlapping cohorts, and separation of diagnostic screening from severity and change measurement. However, restriction to English- and Chinese-language reports may have introduced language bias; the evidence base was dominated by US and predominantly male samples. The included evidence also covered a wide range of post-injury stages, from early rehabilitation to chronic community living; however, few studies were specifically designed to evaluate whether measurement properties differed across SCI stages. Similarly, although age ranges were often broad, evidence specifically supporting use of depressive-symptom PROMs in older adults with SCI remained limited; many reports were old or incompletely reported, and methodological heterogeneity precluded meta-analysis. The available diagnostic evidence was limited by the small number of interview-defined major depressive disorder cases (*n* = 14) in the single PHQ-9 diagnostic study. Although reviewer-generated hypotheses were specified before examination of the relevant results, this process necessarily involved judgement and may have introduced some review-level subjectivity. Eligible SCI-specific study-based content-validity evidence was limited to the SCI-QOL Depression Item Bank and the Lebanese Arabic PHQ-9; most integrated conclusions therefore relied on reviewer assessment and were of very-low certainty. Some legacy, research-derived, or translated forms remained partly indeterminate because authoritative materials or language-specific assessments were unavailable. Diagnostic indices were synthesized within a COSMIN framework rather than a dedicated diagnostic-accuracy review, and publication bias was not formally assessed.

### Conclusion

No depressive-symptom PROM currently has a sufficiently complete evidence profile to support routine use across the intended applications in adults with SCI. The SCI-QOL Depression Item Bank is the most developed SCI-specific candidate for cross-sectional severity assessment. Its integrated content-validity ratings were sufficient, but comprehensibility was supported only by very-low-certainty reviewer assessment, measurement invariance was insufficient, and evidence for measurement error, minimal important change, and responsiveness was absent. The English PHQ-9 may contribute to case finding only as supplementary evidence followed by clinical assessment; its conventional total score should not be interpreted as a clearly unidimensional continuous severity measure, and item-level responses and clinical context should inform interpretation. English HADS-D may be considered when limiting somatic content is important, but its content coverage and longitudinal properties require further study. Future research should prioritize patient-informed content-validity studies and rigorous longitudinal validation.

## Supplementary Material


