# Supplementary material for: PATIENT-REPORTED OUTCOME MEASURES FOR DEPRESSIVE SYMPTOMS IN ADULTS WITH SPINAL CORD INJURY: A COSMIN SYSTEMATIC REVIEW
Source: J Rehabil Med. 2026 Oct 28;58:46626. doi: 10.2340/jrm.v58.46626 (PMC13722993; doi:10.2340/jrm.v58.46626)
Supplement: Supplementary file 1 [file JRM-58-46626-s1.pdf]

Table SI. Complete database-specific search strategies

Patient-reported outcome measures for depressive symptoms in adults with spinal cord injury: a COSMIN systematic review

Search cutoff: from database inception to 25 June 2026.

Total records identified from the databases listed below: n = 7,257.

Reporting note: Search strategies for Chinese-language databases are reported below in English translation for journal submission; the searches were executed using the corresponding Chinese-language terms in each database interface.
Scope note: The terms “mood disorder*” and “dysphori*” were used as sensitivity terms to capture older or variably indexed depression-related literature. They did not define eligibility; studies were included only when they evaluated a depressive-symptom PROM, an independently scored depression subscale, or an explicitly derived depression score in adults with SCI.

Summary of Database Searches

No.	Database	Platform/interface	Records identified
1	**PubMed/MEDLINE**	PubMed	927
2	**Web of Science Core Collection**	Web of Science	1,228
3	**CINAHL**	EBSCOhost	1,204
4	**Scopus**	Scopus	1,543
5	**APA PsycINFO**	Ovid	153
6	**Embase**	Embase.com	666
7	**CNKI**	CNKI advanced search	238
8	**Wanfang Data**	Wanfang advanced search	572
9	**VIP**	VIP advanced search	310
10	**SinoMed**	SinoMed advanced search	416

Detailed Search Strategies

PubMed/MEDLINE

Item	Details
**Database/platform**	PubMed/MEDLINE; PubMed
**Search cutoff**	From database inception to 25 June 2026
**Records identified**	n = 927
**Search notes**	Date limit embedded in the search string.
**Search strategy**	(("Spinal Cord Injuries"[Mesh] OR "spinal cord injur*"[tiab] OR "spinal cord lesion*"[tiab] OR "spinal cord trauma*"[tiab] OR "spinal cord disorder*"[tiab] OR "spinal cord disease*"[tiab] OR paraplegi*[tiab] OR tetraplegi*[tiab] OR quadriplegi*[tiab] OR "SCI"[tiab] OR "SCI/D"[tiab]) AND ("Depression"[Mesh] OR "Depressive Disorder"[Mesh] OR "Depressive Disorder, Major"[Mesh] OR "Dysthymic Disorder"[Mesh] OR depress*[tiab] OR "depressive symptom*"[tiab] OR dysphori*[tiab]

Item	Details
	OR "mood disorder*"[tiab] OR "Beck Depression Inventory"[tiab] OR BDI[tiab] OR "BDI-II"[tiab] OR "Center for Epidemiologic Studies Depression"[tiab] OR "Centre for Epidemiologic Studies Depression"[tiab] OR "CES-D"[tiab] OR CESD[tiab] OR "Patient Health Questionnaire"[tiab] OR PHQ[tiab] OR "PHQ-9"[tiab] OR "PHQ-2"[tiab] OR "Hospital Anxiety and Depression Scale"[tiab] OR HADS[tiab] OR "HADS-D"[tiab] OR "Zung Self-Rating Depression Scale"[tiab] OR "Self-Rating Depression Scale"[tiab] OR "Depression Anxiety Stress Scale"[tiab] OR DASS[tiab] OR "Brief Symptom Inventory"[tiab] OR BSI[tiab] OR "Symptom Checklist-90"[tiab] OR "SCL-90"[tiab] OR "Ilfeld Psychiatric Symptom Index"[tiab] OR "Older Adult Health and Mood Questionnaire"[tiab] OR OAHMQ[tiab] OR "PROMIS Depression"[tiab] OR "SCI-QOL Depression"[tiab]) AND ("Patient Reported Outcome Measures"[Mesh] OR "Surveys and Questionnaires"[Mesh] OR "Psychometrics"[Mesh] OR "Reproducibility of Results"[Mesh] OR "Validation Study"[Publication Type] OR questionnaire*[tiab] OR scale*[tiab] OR instrument*[tiab] OR inventory[tiab] OR inventories[tiab] OR measure*[tiab] OR assessment*[tiab] OR "self report"[tiab] OR "self reported"[tiab] OR "self-report"[tiab] OR "self-reported"[tiab] OR PROM[tiab] OR PROMs[tiab] OR "patient reported outcome*"[tiab] OR "patient-reported outcome*"[tiab] OR "screening tool*"[tiab] OR "screening instrument*"[tiab]) AND (psychometr*[tiab] OR clinimetr*[tiab] OR clinometr*[tiab] OR "outcome assessment"[tiab] OR "outcome measure*"[tiab] OR reproducib*[tiab] OR reliab*[tiab] OR valid*[tiab] OR "internal consistency"[tiab] OR Cronbach*[tiab] OR "test retest"[tiab] OR "test-retest"[tiab] OR ICC[tiab] OR kappa[tiab] OR "measurement error"[tiab] OR SEM[tiab] OR responsiveness[tiab] OR "factor analysis"[tiab] OR "factor structure"[tiab] OR "structural validity"[tiab] OR Rasch[tiab] OR "item response theor*"[tiab] OR IRT[tiab] OR DIF[tiab] OR "measurement invariance"[tiab] OR "construct validity"[tiab] OR "content validity"[tiab] OR "criterion validity"[tiab] OR "convergent validity"[tiab] OR "discriminant validity"[tiab] OR interpretability[tiab] OR feasibility[tiab])) AND ("1800/01/01"[dp] : "2026/06/25"[dp])

Web of Science Core Collection

Item	Details
**Database/platform**	Web of Science Core Collection; Advanced Search
**Search cutoff**	From database inception to 25 June 2026
**Records identified**	n = 1,228
**Search notes**	Topic field was used. The date cutoff was applied in the database interface/export.
**Search strategy**	TS=("spinal cord injur*" OR "spinal cord lesion*" OR "spinal cord trauma*" OR "spinal cord disorder*" OR "spinal cord disease*" OR paraplegi* OR tetraplegi* OR quadriplegi* OR "SCI" OR "SCI/D") AND TS=(depress* OR "depressive symptom*" OR dysphori* OR "mood disorder*" OR "Beck Depression Inventory" OR BDI OR "BDI-II" OR "Center for Epidemiologic Studies Depression" OR "Centre for Epidemiologic Studies Depression" OR "CES-D" OR CESD OR "Patient Health Questionnaire" OR PHQ OR "PHQ-9" OR "PHQ-2" OR "Hospital Anxiety and Depression Scale" OR HADS OR "HADS-D" OR DASS OR BSI OR "SCL-90" OR OAHMQ OR "PROMIS Depression" OR "SCI-QOL Depression") AND TS=("Patient Reported Outcome Measures" OR "patient reported outcome*" OR "patient-reported outcome*" OR questionnaire* OR scale* OR instrument* OR inventory OR inventories OR measure* OR assessment* OR "self report" OR "self reported" OR "self-

Item	Details
	report" OR "self-reported" OR PROM OR PROMs OR "screening tool*" OR "screening instrument*") AND TS=(psychometr* OR clinimetr* OR clinometr* OR valid* OR reliab* OR reproducib* OR responsiveness OR "internal consistency" OR Cronbach* OR "test retest" OR "test-retest" OR ICC OR kappa OR "measurement error" OR SEM OR "factor analysis" OR "factor structure" OR "structural validity" OR dimensionality OR Rasch OR "item response theor*" OR IRT OR DIF OR "measurement invariance" OR "construct validity" OR "content validity" OR "criterion validity" OR interpretability OR feasibility)

CINAHL

Item	Details
**Database/platform**	CINAHL; EBSCOhost
**Search cutoff**	From database inception to 25 June 2026
**Records identified**	n = 1,204
**Search notes**	Date limit was not embedded because this caused retrieval errors; records were limited/screened to the cutoff date.
**Search strategy**	S1: (MH "Spinal Cord Injuries+") OR TI ("spinal cord injur*" OR "spinal cord lesion*" OR "spinal cord trauma*" OR "spinal cord disorder*" OR "spinal cord disease*" OR paraplegi* OR tetraplegi* OR quadriplegi* OR SCI) OR AB ("spinal cord injur*" OR "spinal cord lesion*" OR "spinal cord trauma*" OR "spinal cord disorder*" OR "spinal cord disease*" OR paraplegi* OR tetraplegi* OR quadriplegi* OR SCI)<br>S2: (MH "Depression+") OR (MH "Depressive Disorder+") OR TI (depress* OR "depressive symptom*" OR dysphori* OR "mood disorder*" OR "Beck Depression Inventory" OR BDI OR "BDI-II" OR "CES-D" OR CESD OR "Patient Health Questionnaire" OR PHQ OR "PHQ-9" OR "PHQ-2" OR "Hospital Anxiety and Depression Scale" OR HADS OR "HADS-D" OR DASS OR BSI OR "SCL-90" OR OAHMQ OR "PROMIS Depression" OR "SCI-QOL Depression") OR AB (depress* OR "depressive symptom*" OR dysphori* OR "mood disorder*" OR "Beck Depression Inventory" OR BDI OR "BDI-II" OR "CES-D" OR CESD OR "Patient Health Questionnaire" OR PHQ OR "PHQ-9" OR "PHQ-2" OR "Hospital Anxiety and Depression Scale" OR HADS OR "HADS-D" OR DASS OR BSI OR "SCL-90" OR OAHMQ OR "PROMIS Depression" OR "SCI-QOL Depression")<br>S3: (MH "Patient Reported Outcome Measures+") OR (MH "Questionnaires+") OR TI ("patient reported outcome*" OR "patient-reported outcome*" OR questionnaire* OR scale* OR instrument* OR inventory OR inventories OR measure* OR assessment* OR "self report" OR "self reported" OR "self-report" OR "self-reported" OR PROM OR PROMs OR "screening tool*" OR "screening instrument*") OR AB ("patient reported outcome*" OR "patient-reported outcome*" OR questionnaire* OR scale* OR instrument* OR inventory OR inventories OR measure* OR assessment* OR "self report" OR "self reported" OR "self-report" OR "self-reported" OR PROM OR PROMs OR "screening tool*" OR "screening instrument*")<br>S4: (MH "Psychometrics+") OR (MH "Reproducibility of Results+") OR TI (psychometr* OR clinimetr* OR clinometr* OR valid* OR reliab* OR reproducib* OR responsiveness OR "internal consistency" OR Cronbach* OR "test retest" OR "test-retest" OR ICC OR kappa OR "measurement error" OR SEM OR "factor analysis" OR "factor structure" OR "structural validity" OR Rasch OR "item response theor*" OR IRT OR DIF OR "measurement invariance" OR "construct validity" OR "content validity" OR "criterion validity") OR AB (psychometr* OR clinimetr* OR clinometr* OR

Item	Details
	valid* OR reliab* OR reproducib* OR responsiveness OR "internal consistency" OR Cronbach* OR "test retest" OR "test-retest" OR ICC OR kappa OR "measurement error" OR SEM OR "factor analysis" OR "factor structure" OR "structural validity" OR Rasch OR "item response theor*" OR IRT OR DIF OR "measurement invariance" OR "construct validity" OR "content validity" OR "criterion validity")<br>Final: S1 AND S2 AND S3 AND S4

Scopus

Item	Details
**Database/platform**	Scopus
**Search cutoff**	From database inception to 25 June 2026
**Records identified**	n = 1,543
**Search notes**	TITLE-ABS-KEY field was used. Date limit was not embedded because this caused retrieval errors; records were limited/screened to the cutoff date.
**Search strategy**	TITLE-ABS-KEY("spinal cord injur*" OR "spinal cord lesion*" OR "spinal cord trauma*" OR "spinal cord disorder*" OR "spinal cord disease*" OR paraplegi* OR tetraplegi* OR quadriplegi* OR "SCI" OR "SCI/D") AND TITLE-ABS-KEY(depress* OR "depressive symptom*" OR dysphori* OR "mood disorder*" OR "Beck Depression Inventory" OR BDI OR "BDI-II" OR "CES-D" OR CESD OR "Patient Health Questionnaire" OR PHQ OR "PHQ-9" OR "PHQ-2" OR "Hospital Anxiety and Depression Scale" OR HADS OR "HADS-D" OR DASS OR BSI OR "SCL-90" OR OAHMQ OR "PROMIS Depression" OR "SCI-QOL Depression") AND TITLE-ABS-KEY("Patient Reported Outcome Measures" OR "patient reported outcome*" OR "patient-reported outcome*" OR questionnaire* OR scale* OR instrument* OR inventory OR inventories OR measure* OR assessment* OR "self report" OR "self reported" OR "self-report" OR "self-reported" OR PROM OR PROMs OR "screening tool*" OR "screening instrument*") AND TITLE-ABS-KEY(psychometr* OR clinimetr* OR clinometr* OR valid* OR reliab* OR reproducib* OR responsiveness OR "internal consistency" OR Cronbach* OR "test retest" OR "test-retest" OR ICC OR kappa OR "measurement error" OR SEM OR "factor analysis" OR "factor structure" OR "structural validity" OR dimensionality OR Rasch OR "item response theor*" OR IRT OR DIF OR "measurement invariance" OR "construct validity" OR "content validity" OR "criterion validity" OR interpretability OR feasibility)

APA PsycINFO

Item	Details
**Database/platform**	APA PsycINFO; Ovid
**Search cutoff**	From database inception to 25 June 2026
**Records identified**	n = 153
**Search notes**	Searched using Ovid APA PsycINFO title/abstract fields.
**Search strategy**	Ovid APA PsycINFO<br>1. ("spinal cord injur*" OR "spinal cord lesion*" OR "spinal cord trauma*" OR "spinal cord disorder*" OR "spinal cord disease*" OR paraplegi* OR tetraplegi* OR quadriplegi* OR SCI).ti,ab.<br>2. (depress* OR "depressive symptom*" OR dysphori* OR "mood disorder*" OR "Beck Depression Inventory" OR BDI OR "BDI-II" OR "CES-D" OR CESD OR "Patient Health Questionnaire" OR PHQ OR "PHQ-9" OR "PHQ-2" OR "Hospital Anxiety and Depression Scale" OR HADS OR "HADS-D" OR DASS OR BSI OR "SCL-90" OR OAHMQ OR "PROMIS Depression" OR "SCI-QOL Depression").ti,ab.<br>3. ("Patient Reported Outcome Measures" OR "patient reported outcome*" OR "patient-reported outcome*" OR questionnaire* OR scale* OR instrument* OR inventory OR inventories OR measure* OR assessment* OR "self report" OR "self reported" OR "self-report" OR "self-reported" OR PROM OR PROMs OR "screening tool*" OR "screening instrument*").ti,ab.<br>4. (psychometr* OR clinimetr* OR clinometr* OR valid* OR reliab* OR reproducib* OR "internal consistency" OR Cronbach* OR "test retest" OR "test-retest" OR ICC OR kappa OR "measurement error" OR SEM OR responsiveness OR "factor analysis" OR "factor structure" OR "structural validity" OR dimensionality OR Rasch OR "item response theor*" OR IRT OR DIF OR "measurement invariance" OR "construct validity" OR "content validity" OR "criterion validity" OR interpretability OR feasibility).ti,ab.<br>5. 1 AND 2 AND 3 AND 4

Embase

Item	Details
**Database/platform**	Embase; Embase.com
**Search cutoff**	From database inception to 25 June 2026
**Records identified**	n = 666
**Search notes**	Searched using Embase.com. Emtree explosion terms and title/abstract/keyword fields were used.
**Search strategy**	('spinal cord injury'/exp OR 'spinal cord injury' OR 'spinal cord injur*':ti,ab,kw OR 'spinal cord lesion*':ti,ab,kw OR 'spinal cord trauma*':ti,ab,kw OR 'spinal cord disorder*':ti,ab,kw OR 'spinal cord disease*':ti,ab,kw OR paraplegi*:ti,ab,kw OR tetraplegi*:ti,ab,kw OR quadriplegi*:ti,ab,kw OR sci:ti,ab,kw OR 'SCI/D':ti,ab,kw) AND ('depression'/exp OR 'major

Item	Details
	depression'/exp OR 'dysthymia'/exp OR depress*:ti,ab,kw OR 'depressive symptom*':ti,ab,kw OR dysphori*:ti,ab,kw OR 'mood disorder*':ti,ab,kw OR 'Beck Depression Inventory':ti,ab,kw OR BDI:ti,ab,kw OR 'BDI-II':ti,ab,kw OR 'CES-D':ti,ab,kw OR CESD:ti,ab,kw OR 'Patient Health Questionnaire':ti,ab,kw OR PHQ:ti,ab,kw OR 'PHQ-9':ti,ab,kw OR 'PHQ-2':ti,ab,kw OR 'Hospital Anxiety and Depression Scale':ti,ab,kw OR HADS:ti,ab,kw OR 'HADS-D':ti,ab,kw OR DASS:ti,ab,kw OR BSI:ti,ab,kw OR 'SCL-90':ti,ab,kw OR OAHMQ:ti,ab,kw OR 'PROMIS Depression':ti,ab,kw OR 'SCI-QOL Depression':ti,ab,kw) AND ('patient reported outcome*':ti,ab,kw OR 'patient-reported outcome*':ti,ab,kw OR questionnaire*:ti,ab,kw OR scale*:ti,ab,kw OR instrument*:ti,ab,kw OR inventory:ti,ab,kw OR inventories:ti,ab,kw OR measure*:ti,ab,kw OR assessment*:ti,ab,kw OR 'self report':ti,ab,kw OR 'self reported':ti,ab,kw OR 'self-report':ti,ab,kw OR 'self-reported':ti,ab,kw OR PROM:ti,ab,kw OR PROMs:ti,ab,kw OR 'screening tool*':ti,ab,kw OR 'screening instrument*':ti,ab,kw) AND (psychometr*:ti,ab,kw OR clinimetr*:ti,ab,kw OR clinometr*:ti,ab,kw OR valid*:ti,ab,kw OR reliab*:ti,ab,kw OR reproducib*:ti,ab,kw OR responsiveness:ti,ab,kw OR 'internal consistency':ti,ab,kw OR Cronbach*:ti,ab,kw OR 'test retest':ti,ab,kw OR 'test-retest':ti,ab,kw OR ICC:ti,ab,kw OR kappa:ti,ab,kw OR 'measurement error':ti,ab,kw OR SEM:ti,ab,kw OR 'minimal important difference':ti,ab,kw OR MID:ti,ab,kw OR MCID:ti,ab,kw OR MIC:ti,ab,kw OR 'minimal detectable change':ti,ab,kw OR MDC:ti,ab,kw OR SDC:ti,ab,kw OR 'factor analysis':ti,ab,kw OR 'factor structure':ti,ab,kw OR 'structural validity':ti,ab,kw OR dimensionality:ti,ab,kw OR Rasch:ti,ab,kw OR 'item response theor*':ti,ab,kw OR IRT:ti,ab,kw OR DIF:ti,ab,kw OR 'measurement invariance':ti,ab,kw OR 'construct validity':ti,ab,kw OR 'content validity':ti,ab,kw OR 'criterion validity':ti,ab,kw OR interpretability:ti,ab,kw OR feasibility:ti,ab,kw)

CNKI

Item	Details
**Database/platform**	CNKI advanced search
**Search cutoff**	From database inception to 25 June 2026
**Records identified**	n = 238
**Search notes**	English translation of the Chinese search strategy used in CNKI.
**Search strategy**	Advanced search; field: Topic; source type: all journals; Chinese-English extension enabled.<br>(spinal cord injury OR paraplegia) AND (depression OR depressive symptoms) AND (scale OR questionnaire)<br>Note: The search was executed in CNKI using the corresponding Chinese-language terms.

Wanfang Data

Item	Details
**Database/platfor m**	Wanfang advanced search
**Search cutoff**	From database inception to 25 June 2026
**Records identified**	n = 572
**Search notes**	English translation of the Chinese search strategy used in Wanfang.
**Search strategy**	Advanced search; fields: Topic/Keyword; source type: all journals.<br>S1: spinal cord injury OR spinal cord lesion OR spinal cord damage OR spinal cord trauma OR paraplegia OR tetraplegia OR quadriplegia OR SCI<br>S2: depression OR depressive symptoms OR depressive disorder OR mood disorder OR Beck Depression Inventory OR BDI OR CES-D OR PHQ OR HADS<br>S3: patient-reported outcome OR patient-reported outcome measure OR patient-reported outcome measure tool OR self-report OR questionnaire OR scale OR screening OR assessment OR measurement<br>S4: psychometrics OR measurement science OR measurement property OR reliability OR validity OR reproducibility OR internal consistency OR Cronbach OR test-retest OR factor analysis OR Rasch OR item response theory OR measurement invariance OR responsiveness OR interpretability OR feasibility<br>Final: S1 AND S2 AND S3 AND S4<br>Note: The search was executed in Wanfang using the corresponding Chinese-language terms.

VIP

Item	Details
**Database/platfor m**	VIP advanced search
**Search cutoff**	From database inception to 25 June 2026
**Records identified**	n = 310
**Search notes**	English translation of the Chinese search strategy used in VIP.
**Search strategy**	Advanced search; field: Any field; journal scope: all journals; matching mode: fuzzy.<br>(spinal cord injury OR paraplegia) AND (depression OR depressive symptoms) AND (scale OR questionnaire OR self-report OR assessment OR screening OR measurement OR reliability OR validity)<br>Note: The search was executed in VIP using the corresponding Chinese-language terms.

SinoMed

Item	Details
**Database/platform**	SinoMed advanced search
**Search cutoff**	From database inception to 25 June 2026
**Records identified**	n = 416
**Search notes**	English translation of the Chinese search strategy used in SinoMed.
**Search strategy**	Advanced search; field: common fields/smart search.<br>(spinal cord injury OR paraplegia) AND (depression OR depressive symptoms) AND (scale OR questionnaire OR self-report OR assessment OR screening OR measurement OR reliability OR validity)<br>Note: The search was executed in SinoMed using the corresponding Chinese-language terms.

Table SII. Full-text reports excluded and primary reasons for exclusion

Notes: One primary exclusion reason was assigned to each report. The grouped reasons correspond exactly to the PRISMA flow diagram: ineligible target construct (*n*=1); no eligible measurement-property evaluation of a depression PROM (*n*=1); no independently scored depression measure (*n*=1); narrative review (*n*=1); and pediatric or mixed-age population with no separable adult data (*n*=2). Reports are ordered by screening ID.

Abbreviations: BSI, Brief Symptom Inventory; CES-D, Center for Epidemiologic Studies Depression Scale; CIS, Clinical Interview Schedule; DSM, Diagnostic and Statistical Manual of Mental Disorders; GHQ-28, 28-item General Health Questionnaire; IDD, Inventory to Diagnose Depression; OMI, outcome measurement instrument; PROM, patient-reported outcome measure; SCI, spinal cord injury; TDI, Thai Depression Inventory.

Screening ID	Full-text report	Why the report appeared potentially eligible	Primary reason for exclusion	Detailed rationale
4	Kuptniratsaikul V, Chulakadabba S, Ratanavijitrasil S. An instrument for assessment of depression among spinal cord injury patients: comparison between the CES-D and TDI. J Med Assoc Thai. 2002;85(9):978–983.	Compared the Thai CES-D and Thai Depression Inventory with psychiatrist-assigned DSM-IV depression diagnoses in 83 people with SCI; receiver operating characteristic and screening-accuracy results were reported.	Pediatric or mixed-age population with no separable adult data	The sample age range was 10–68 years. Pediatric and adult participants were analysed together, and adult-specific results were not reported or extractable.
23	Clay DL, Hagglund KJ, Frank RG, Elliott TR, Chaney JM. Enhancing the accuracy of depression diagnosis in patients with spinal cord injury using Bayesian analysis. Rehabil Psychol. 1995;40(3):171–180.	Used the self-reported Inventory to Diagnose Depression in 133 people with SCI (80 with paraplegia and 53 with quadriplegia) and examined depression-related diagnostic symptoms.	No eligible measurement-property evaluation of a depression PROM	The analysis concerned the positive and negative predictive power and diagnostic efficiency of individual DSM-III-R symptoms. It did not evaluate a COSMIN measurement property of the IDD total score, an IDD subscale, or another independently scored depressive-symptom PROM.
29	Griffiths TC, Myers DH, Talbot AW. A study of the validity of the scaled version of the General Health Questionnaire in paralysed spinally injured out-patients. Psychol Med. 1993;23:497–504.	Evaluated the GHQ-28 against the Clinical Interview Schedule in 60 spinally injured outpatients and reported latent validity, reliability, receiver operating characteristic results, sensitivity, and specificity.	No independently scored depression measure	The GHQ-28 assessed general non-psychotic psychiatric morbidity and overall psychological distress. It did not provide a distinct, independently scored depressive-symptom measure or depression subscale.
30	Overholser JC, Schubert DSP. Depression in patients with spinal cord injuries: a synthesis of cognitive and somatic processes. Curr Psychol. 1993;12(2):172–183.	Addressed the assessment and interpretation of cognitive and somatic depressive symptoms after SCI and discussed implications for clinical identification of depression.	Narrative review	This was a conceptual narrative synthesis. It did not report an original participant sample, PROM development study, or evaluation of a COSMIN measurement property.
32	Heinrich RK, Tate DG, Buckelew SP. Brief Symptom Inventory norms for spinal cord injury. Rehabil Psychol. 1994;39(1):49–56.	Reported SCI-specific BSI normative scores and screening guidance in 225 people with traumatic SCI, including descriptive results for the BSI Depression subscale.	Pediatric or mixed-age population with no separable adult data	Eligibility permitted participants aged 17–68 years. Results for adults aged ≥18 years were not reported separately or extractable from the mixed-age sample.
33	Tate DG, Heinrich RK, Maynard F, Buckelew SP. Moderator-variable effect on the Brief Symptom Inventory test-item endorsements of spinal cord injury patients. Paraplegia. 1994;32:473–479.	Compared BSI responses in 288 adults with traumatic SCI with a 719-person non-patient normative sample and reported descriptive means for all BSI subscales, including Depression.	Ineligible target construct	The target construct was overall psychological distress and SCI-related moderator effects on responses to the complete 53-item BSI and its global indices. The Depression subscale was reported descriptively but was not evaluated as an independent target measure of depressive symptoms.

Table SIII. Interpretability, feasibility, and accessibility of depressive-symptom PROMs evaluated in adults with spinal cord injury

Notes: NR, not reported; CAT, computer adaptive test; MIC, minimal important change; MID, minimal important difference; SCI, spinal cord injury. Cut-offs are reproduced as reported and should not be interpreted as universally valid diagnostic thresholds. Apparent article-level numerical or reporting inconsistencies were reproduced as reported and explicitly flagged. No credible SCI-specific MIC/MID was identified for any PROM. The SCI-QOL Depression Short Form 10a and post hoc PHQ/HSCL forms were not independently validated as separately administered instruments in the included reports. Current-access information was checked on 29 September 2026 using official rights-holder, instrument-developer, or distributor sources where available, supplemented by established implementation repositories and published sources when no current official licensing source could be identified (NLM/NIH CDE and NCBI for CES-D; Pfizer/PHQ Screeners for PHQ; Pearson Assessments and Beck Institute for BSI/Beck/SCL-90-R; GL Assessment/Mapi Research Trust for HADS; UNSW DASS; HealthMeasures/PROMIS; and SCI-QOL developer publications and the RehabMeasures Database). Where no current authoritative rights-holder or licensing source was identified, access status was reported as not established rather than inferred from publication availability. Public availability does not necessarily imply unrestricted reuse. Licensing, registration, platform, and fee conditions may change and should be reconfirmed before implementation. Current-access information is implementation information and is not a COSMIN measurement property.

PROM and version/form	Items, *n*	Administration burden and mode	Accessibility, licensing, registration, cost, and translations	Floor/ceiling effects	Missing data/completeness	Reference scores and cut-offs	MIC/ MID
CES-D-20 (English) Original 20-item version	20	Exact completion time NR. Self-report; McColl and Skinner used trained interviewers in person or by telephone; Miller et al. used laboratory self-report, with four follow-ups returned by telephone/e-mail; Cook et al. primarily used mailed paper questionnaires.	**Included reports:** Item content was reproduced in the included Miller report. The authors described the measure as quick, easy, and low cost; exact licensing terms and costs were NR. Translations were NR in the included reports. **Current access (Sep 2026):** The English CES-D is publicly accessible through U.S. government repositories and is widely described as freely available. No current licence-fee, permission-request, or registration pathway was identified for routine paper use. Because copyright metadata are not fully concordant across repositories, requirements should be reconfirmed before redistribution or electronic/commercial implementation. (NLM/NIH CDE; NCBI)	Miller et al. stated that <15% scored at either scale extreme, but exact minimum- and maximum-score percentages were NR. Cook et al. reported score bands, not exact floor or ceiling frequencies.	Miller et al.: 55 recruited; five lacked session-2 data, two had major interval changes, and one exceeded the retest interval, leaving *n*=47. McColl and Skinner reported no more than six missing responses for any item. Cook et al.: *n*=238 complete SCI records; exclusions for missing data NR.	McColl and Skinner: 29.5%, 31.0%, and 30.5% scored >=16 at 1, 4, and 12 months. Miller et al.: mean 15.2, range 0-42; 39% scored >15 and 30% >19. Cut-offs were >=16, >15, and >19 as reported; none was established as an SCI-specific diagnostic gold-standard threshold.	NR
Zung Self-Rating Depression Scale (English) Original 20-item version	20	<10 min. Self-report; orally administered in Tate et al. and self-administered in Campagnolo et al.	**Included reports:** Full-copy availability, licensing, and cost were NR. Multiple translations were noted, but specific versions were not reported. **Current access (Sep 2026):** No current authoritative instrument-owner or licensing portal was identified. Published copies do not by themselves establish unrestricted reuse; free routine clinical use, registration requirements, and commercial/electronic reuse terms should therefore be confirmed before implementation.	Floor and ceiling effects were NR.	Item-level missingness and exclusions were NR.	Tate et al.: outpatient mean 45.7 (*SD* 11.9), with 21.6% scoring >55. Campagnolo et al.: 3/39 (8%) scored >=50; reported means were 34.1 (*SD* 8.3) for all SCI, 35.2 (6.4) for tetraplegia, and 38.2 (9.3) for paraplegia. The pooled SCI mean is internally inconsistent with the subgroup means. Cut-offs: >55 and >=50, respectively.	NR
Brief Symptom Inventory – Depression subscale (English) Six-item Depression subscale of the 53-item BSI	6	The full 53-item BSI takes <10 min. Administration was oral in Tate et al. (1993) and Heinrich and Tate, self-report in Mitchell et al., and not fully specified in Tate et al. (1990).	**Included reports:** Full-copy availability, licensing, and cost were NR in the included reports. Multiple translations were noted but not specified. **Current access (Sep 2026):** Pearson distributes the BSI as a proprietary Qualification Level B assessment. Test materials and report usages are purchased through Pearson, with qualification/account requirements; it is not free by default for routine clinical use. The SCI-specific research factor remains subject to the parent BSI item rights. (Pearson Assessments)	Floor and ceiling effects were NR.	Tate et al. (1990): *n*=79, with a paired BSI/SCL-90-R subsample of *n*=20. Heinrich and Tate: structural sample *n*=215; follow-up *n*=204, with 11 missing follow-up. Item-level missingness was NR.	Tate et al. (1993): outpatient mean T score 56.6 (*SD* 11.7); 25.6% had T>=65. Mitchell et al.: mean T score 55.95 (*SD* 12.49), range 42-80; 25% exceeded the traditional cutoff and 5% an SCI-specific elevated cutoff. T>=65 was reported by Tate et al.; Mitchell et al. did not report the numerical cutoff values.	NR

PROM and version/form	Items, *n*	Administration burden and mode	Accessibility, licensing, registration, cost, and translations	Floor/ceiling effects	Missing data/completeness	Reference scores and cut-offs	MIC/ MID
Beck Depression Inventory-I (English) Original 21-item version (BDI-I)	21	Completion time NR. Self-administered when participants could write; research assistants provided help for physical writing limitations.	**Included reports:** Full-copy availability, licensing, cost, and translations were NR. **Current access (Sep 2026):** The study evaluated the legacy BDI-I. Beck Institute directs Beck scales to Pearson, which currently markets the BDI-II as a proprietary Qualification Level B product; no current BDI-I-specific distribution route was identified. Free reuse of BDI-I should not be assumed; first-edition permissions should be confirmed with Pearson or the relevant rights holder. (Beck Institute; Pearson Assessments)	Floor and ceiling effects were NR.	Final analytic *n*=124; one recruited participant did not complete the assessment before the analytic sample was defined. Item-level missingness was NR.	Mean BDI score 8.98 (*SD* 10.16); 12/124 met SCID-*P* criteria for a current major depressive episode. Conventional cut-offs 13 and 21 were discussed. SCI candidate cut-offs 16-30 were examined; 18 emphasized sensitivity and 27 specificity/overall classification.	NR
Ilfeld Psychiatric Symptom Index - Depression subscale (English) Depression subscale of the 29-item Ilfeld PSI	10 (inferred from the reproduce d depression -item subset)	Described as not time consuming; exact time NR. Self-report.	**Included reports:** The complete 29-item PSI was reproduced with permission; the depression-subscale boundaries were not explicitly labelled. The authors described administration as low cost; exact cost and licensing terms were NR. Translations were NR. **Current access (Sep 2026):** No current authoritative distributor or instrument-specific licensing/registration terms were identified. Because the included report reproduced the full PSI with permission, unrestricted reuse should not be assumed; permission should be confirmed with the relevant rights holder or publisher before reproducing items for clinical or research use.	Floor and ceiling effects were NR.	Item-level missingness and exclusions were NR.	The depression subset comprised 10 contiguous items in the reproduced 29-item PSI. Reported means were 17.7 (*SD* 14.5) for all SCI, 16.2 (12.1) for tetraplegia, 23.9 (18.1) for paraplegia, and 13.3 (11.2) for controls; the pooled SCI mean is internally inconsistent with subgroup means. No depression-subscale cutoff was reported; >=10 applied to the total PSI only.	NR
Patient Health Questionnaire-9 (English) Original 9-item version	9	Usually brief; Williams et al. reported 1-3 min in most cases. Modes included telephone, mail, in-person self-report, and standardized interview; mode varied across studies.	**Included reports:** All nine items were reproduced in included reports. Exact licensing terms, costs, and translations were NR in the included studies. **Current access (Sep 2026):** Pfizer states that the PHQ-9 can be downloaded from PHQ Screeners after acceptance of the terms of use, without a formal permission request; approved translations are also available. No separate licence fee or account-registration requirement is stated for this route. (Pfizer/PHQ Screeners)	Reported floor frequencies at score 0 included 23.4% (Bombardier 2004), 9.5% (Krause 2008), 10.2% (Tulsky 2015 crosswalk), 5.1% (Williams 2016), and 21.4% (Cook 2016). Exact maximum-score frequencies were usually NR; Tulsky reported 0.2%.	Most analyses used complete PHQ-9 records; item-level missingness was generally NR. Examples: 690/727 had severity classification in Krause 2009; 142 paired PHQ-9/SCID assessments were available in Bombardier 2012; 116/144 completed both screeners in Poritz et al.	Bombardier 2004: mean 5.48 (*95% CI* 5.07-5.88); 11.4% met the PHQ algorithm. A total score >=10 was widely used. Against blinded SCID in Bombardier 2012, *AUC*=0.921; cutoff >=11 had sensitivity 1.00 and specificity 0.84, while >=10 had sensitivity 1.00 and specificity 0.80. Severity bands were 0, 1-4, 5-9, 10-14, 15-19, and 20-27.	NR
Hospital Anxiety and Depression Scale - Depression subscale (English) Depression subscale of the 14-item HADS	7	Brief; exact time NR. Postal self-report completed at home.	**Included reports:** All HADS items were reproduced in the included report. Licensing, cost, and translations were NR. **Current access (Sep 2026):** HADS is copyright-protected in all languages. Permission/licensing is required, and current clinical-outcome guidance reports a user fee whose amount depends on context. UK/Republic of Ireland/Channel Islands requests are handled by GL Assessment; other-country licensing and translations are handled through Mapi/ePROVIDE. It is not free by default for routine clinical use. (GL Assessment; Mapi Research Trust)	Floor and ceiling effects were NR.	Of 985 participants, 963 completed the HADS; 22 did not.	Mean HADS-D 5.5 (*SD* 3.7); 20.8% scored >8. The report used >8 for likely clinically significant depression, while also citing severity bands of 8-10 mild, 11-15 moderate, and >=16 severe; this threshold wording is internally inconsistent and is retained as reported.	NR

PROM and version/form	Items, *n*	Administration burden and mode	Accessibility, licensing, registration, cost, and translations	Floor/ceiling effects	Missing data/completeness	Reference scores and cut-offs	MIC/ MID
CES-D-10 (English) 10-item short form	10	Exact completion time NR. Self-report; initial laboratory assessment and approximately 2-week follow-up, with four follow-ups returned by telephone/e-mail.	**Included reports:** The 10 retained items were identified in the included report. The authors described the form as quick, easy, and low cost; exact licensing terms and costs were NR. Translations were NR. **Current access (Sep 2026):** CES-D-10 is a derived short form of the publicly accessible CES-D item set. No separate licensing or registration pathway was identified for the version evaluated here; the retained-item composition and scoring should be cited to the source study. Requirements for redistribution or electronic/commercial implementation should be reconfirmed. (NLM/NIH CDE; NCBI)	The authors stated that <15% scored at either extreme; exact floor and ceiling percentages were NR.	Of 55 recruited participants, five lacked session-2 data, two reported major interval changes, and one exceeded the retest interval; final analytic *n*=47. Item-level missingness was NR.	Mean 9.7, range 0-25; 38.3% scored >10. The study used >10 as the cutoff.	NR
Depression Anxiety Stress Scales-21 - Depression subscale (English) Depression subscale of the 21-item DASS-21	7	<10 min for the full DASS-21; subscale-specific time NR. Self-report administered by the first author.	**Included reports:** Full-copy availability, licensing, cost, and translations were NR. **Current access (Sep 2026):** The official DASS site states that the DASS is public domain: permission is not required, and the questionnaire and scoring key may be downloaded and copied without restriction. No registration is stated. Paper use and administration to a restricted patient/research group are permitted; public-facing website/app administration is not. The manual is sold separately. (UNSW DASS)	Floor and ceiling effects were NR.	Item-level missingness and exclusions were NR.	*n*=40: mean 7.8 (*SD* 9.33), range 0-40; 30% exceeded the clinical threshold. MINI identified seven depression diagnoses (17.5%). The report referred to the standard DASS-21 clinical threshold but did not provide its numerical value.	NR
Older Adult Health and Mood Questionnaire (English) Original 22-item version	22	Completion time NR. Postal self-report with repeat mailings and telephone follow-up of nonrespondents.	**Included reports:** Only example items were reproduced; full-copy availability, licensing, cost, and translations were NR. **Current access (Sep 2026):** No authoritative current instrument-owner/distributor or licensing/registration terms were identified. Although the full item set appears in later open-access publications, article-level open access does not establish unrestricted instrument reuse. Free routine clinical use should therefore not be assumed.	Floor and ceiling effects were NR.	727 participants responded; 682 had complete OAHMQ severity classification, leaving 45 without complete classification. Item-level missingness was NR.	Mean 6.0 (*SD* 5.0). Among *n*=682, 55.6% scored 0-5, 24.8% scored 6-10, and 19.7% scored >=11. Original categories were 0-5 non-depressed, 6-10 clinically significant symptoms, and >=11 probable major depression. The Methods stated 11-22 for the highest range, whereas the table labelled 11-20.	NR
Hospital Anxiety and Depression Scale - Depression subscale (German) German HADS-D, 7-item depression subscale	7	Completion time NR. Postal self-report.	**Included reports:** English item labels were reported, but the German wording was not reproduced. Licensing, cost, and other translations were NR. **Current access (Sep 2026):** HADS is copyright-protected in all languages. Permission/licensing is required and fees depend on context; international licensing and translations are handled through GL Assessment/Mapi Research Trust. The German version is not free by default for routine clinical use, and language-specific distribution arrangements should be confirmed before use. (GL Assessment; Mapi Research Trust)	Four percent were below the lowest Rasch threshold, indicating a small floor effect; no ceiling effect was reported.	394 people were invited. The flow diagram and participant table reported 102 responders; the text stated 100 responded and also reported deletion of two incomplete questionnaires. Rasch analyses used *n*=100.	Mean 5.87 (*SD* 3.62), *n*=100; 31% scored >=8 and 21% >=11. Severity bands: 8-10 mild, 11-15 moderate, and >=16 severe.	NR

PROM and version/form	Items, *n*	Administration burden and mode	Accessibility, licensing, registration, cost, and translations	Floor/ceiling effects	Missing data/completeness	Reference scores and cut-offs	MIC/ MID
Hospital Anxiety and Depression Scale - Depression subscale (Turkish) Turkish HADS-D, 7-item depression subscale	7	2-5 min for the complete 14-item HADS. Self-report completed in the outpatient rehabilitation clinic.	**Included reports:** Item wording was not reproduced. Licensing, cost, and translations were NR. **Current access (Sep 2026):** HADS is copyright-protected in all languages. Permission/licensing is required and fees depend on context; international licensing and translations are handled through GL Assessment/Mapi Research Trust. The Turkish version is not free by default for routine clinical use, and language-specific distribution arrangements should be confirmed before use. (GL Assessment; Mapi Research Trust)	Floor and ceiling effects were NR.	All 175 included participants completed the HADS; item-level missingness was NR.	Mean 5.8 (*SD* 3.4), range 0-16; 49/175 (28.0%) scored >7 (>=8). Scores 0-7 were considered normal and >7 indicated probable anxiety or depression. The age correlation was reported as *r*=0.17 in the abstract/text and *r*=0.10 in the study table (both *P*=0.02).	NR
SCI-QOL Depression Item Bank (English) Calibrated 28-item item bank	28	Administration time NR. Structured interview in person or by telephone; electronic administration is supported through Assessment Center.	**Included reports:** All 28 retained item stems were reproduced. Items and parameters are copyrighted; the official instrument should be accessed through Assessment Center and not modified. No administration fee was reported. Translations were NR. **Current access (Sep 2026):** SCI-QOL items are copyrighted and should not be modified without permission. Developer publications describe access through the corresponding author/Assessment Center, and the Rehabilitation Measures Database currently lists SCI-QOL Depression as free, with short-form PDFs available by request and electronic options including REDCap. Because the access page was last updated in 2015, current platform-specific conditions should be reconfirmed. (SCI-QOL developers; RehabMeasures Database)	Full 28-item bank: floor 0.1%, ceiling 3.1%.	Item-level missingness was NR. No response category contained fewer than five observations.	Calibration *n*=716: T-score mean 50.0 (*SD* 9.6), range 32.28-83.86; mean *SE* 2.1. T-scores reference the PROMIS general-population mean of 50 (*SD* 10). No diagnostic cutoff was established.	NR
SCI-QOL Depression CAT (English) Variable-length CAT; default minimum 4, maximum 12, *SE* <0.30	4-12 under default settings	Administration time NR. Computer adaptive administration; independent reliability-sample administrations averaged 5.93 items (*SD* 3.1). Default settings: minimum 4, maximum 12, *SE*<0.30.	**Included reports:** Implemented through Assessment Center under the item-bank copyright and integrity restrictions. No administration fee was reported. Translations were NR. **Current access (Sep 2026):** The CAT uses the same copyrighted SCI-QOL item bank. The Rehabilitation Measures Database currently lists SCI-QOL Depression as free and describes CAT access through REDCap or legacy Assessment Center routes; Assessment Center administration requires a login. Because these platform details were last updated in 2015, current access, registration, and platform requirements should be reconfirmed. (SCI-QOL developers; RehabMeasures Database)	Default variable-length CAT: floor 3.5%, ceiling 0.1%.	Item-level missingness was NR. In the independent reliability sample, 75% completed within six items and 17.7% received the maximum 12 items.	Calibration analysis: mean 6.41 items (*SD* 3.3), range 4-12; T-score mean 50.7 (*SD* 9.5), range 32.84-82.56; mean *SE* 3.0. No diagnostic cutoff was established.	NR

PROM and version/form	Items, *n*	Administration burden and mode	Accessibility, licensing, registration, cost, and translations	Floor/ceiling effects	Missing data/completeness	Reference scores and cut-offs	MIC/MID
SCI-QOL Depression Short Form 10a (English) Fixed 10-item Short Form 10a	10	Fixed 10-item short form derived from the calibrated item bank; administration time and mode were not independently reported.	**Included reports:** The 10 selected item stems and a raw-score-to-T-score lookup table were provided. Official access is through Assessment Center under the item-bank copyright restrictions; no administration fee was reported. Translations were NR. **Current access (Sep 2026):** The fixed short form is covered by the SCI-QOL item-bank copyright and should not be modified without permission. The Rehabilitation Measures Database currently lists SCI-QOL Depression as free and states that short-form PDFs can be requested from the developers; no per-use fee is stated. Current distribution arrangements should be reconfirmed because the access information was last updated in 2015. (SCI-QOL developers; RehabMeasures Database)	Short-form floor and ceiling effects were not separately reported.	Item-level missingness was NR.	Raw scores 10-50 corresponded to T scores 38.3-81.9. No diagnostic cutoff was established. The short form was selected from the calibration sample and was not independently validated in the included report.	NR
Patient Health Questionnaire-9, revised 3-category scoring (English) Post hoc 9-item PHQ-9 with three response categories	9	Post hoc score derived from interview-administered PHQ-9 responses; not independently administered. Completion time NR.	**Included reports:** All nine items and the three-category rescoring rule were reproduced. Original PHQ licensing terms apply; exact terms, cost, and translations were NR. **Current access (Sep 2026):** Access to the underlying PHQ-9 items follows Pfizer/PHQ Screeners terms: download after accepting the terms, with no formal permission request stated. This three-category score is a post hoc research rescoring rather than a separately distributed official PHQ instrument; it should not be presented as a separately licensed PHQ version.	Floor 5.1%; ceiling percentage NR.	884 repeated observations from 133 participants; item-level missingness and exact week-12 *n* were NR.	Score range 0-18. Response was defined as >=50% reduction and remission as PHQ-9<=4, but these thresholds were not independently validated for the revised score.	NR
Hopkins Symptom Checklist-20 Depression Scale, Version B (English) HSCL-20 Depression Scale, Version B; original 20-item form	20	Interview-administered self-report; completion time NR.	**Included reports:** All 20 item stems and the original response structure were reproduced. Licensing, cost, and translations were NR. **Current access (Sep 2026):** No current authoritative central rights-holder/distributor or form-specific licensing/registration route was identified for this HSCL version. Reproduction of item stems in the included study does not establish unrestricted reuse; free routine clinical use should not be assumed without rights confirmation.	Floor 2.9%; ceiling percentage NR.	885 repeated observations from 133 participants; item-level missingness and exact week-12 *n* were NR.	The study defined response as >=50% reduction in raw score; no remission or diagnostic cutoff was available. The original scale had 20 items and five response categories.	NR
Hopkins Symptom Checklist revised 12-item depression scale (English) Post hoc revised form reported as 12 items with three response categories	12 as reported; exact retained-item set not reconstructable	Post hoc score derived from interview responses to the HSCL-20; not independently administered. Completion time NR.	**Included reports:** The report reproduced the 20 source items and identified six misfitting items, but did not provide a reproducible final 12-item set. Licensing, cost, and translations were NR. **Current access (Sep 2026):** No current authoritative licensing route was identified. This 12-item score is research-derived and the exact retained-item set was not reconstructable from the included report; it should not be treated as a separately distributed free instrument. Underlying HSCL item rights should be confirmed before reuse.	Floor 9.7%; ceiling percentage NR.	885 repeated observations from 133 participants; item-level missingness and exact week-12 *n* were NR.	The study table reported 12 items with three response categories. The text called seven items misfitting but listed six; deletion of six from 20 would leave 14, so the final 12-item composition cannot be reconstructed. Response was >=50% reduction; no remission or diagnostic cutoff was available.	NR

PROM and version/form	Items, *n*	Administration burden and mode	Accessibility, licensing, registration, cost, and translations	Floor/ceiling effects	Missing data/completeness	Reference scores and cut-offs	MIC/ MID
PROMIS Depression Short Form 8b, version 1.0 (English)<br>PROMIS Depression Short Form 8b, version 1.0	8	Completion time NR. SCI data were collected primarily by mailed paper-and-pencil survey.	**Included reports:** The form was identified and access through Assessment Center was referenced, but all items were not reproduced. Exact licensing terms, cost, and translations were NR.<br>**Current access (Sep 2026):** HealthMeasures permits English PROMIS paper forms without licensing or royalty fees for individual clinical/research use. PDF download requires acceptance of Terms of Use and provision of name/e-mail. For digital use, a non-commercial single research study may be exempt from HEAP, whereas routine clinical care, registries/quality measurement, and commercial use require platform-specific permission/licensing and may incur fees. Translations require permission and may incur fees. (HealthMeasures/PROMIS)	Floor and ceiling effects were NR.	The DIF analysis included 292 SCI participants with complete PROMIS-D 8b data; the number excluded for missing data was NR.	No SCI-specific mean or diagnostic cutoff was reported. Corrected and uncorrected DIF-adjusted scores were highly correlated (Spearman *r*=0.998); this is not a reference norm or cutoff.	NR
Patient Health Questionnaire-9, Arabic version (Lebanese Arabic)<br>Lebanese Arabic PHQ-9; original 9-item content and 0-3 scoring	9	Pilot completion averaged 4-6 min. Face-to-face standardized administration by two specialists; nurse assistance was provided for writing limitations.	**Included reports:** The complete Arabic form was not reproduced. The investigators obtained the previously validated Lebanese Arabic version with permission; current licensing terms and cost were NR. This was the only Arabic version evaluated.<br>**Current access (Sep 2026):** Pfizer states that approved PHQ translations can be downloaded and used after acceptance of PHQ Screeners terms, without a formal permission request. The included investigators obtained the Lebanese Arabic version with permission; current availability of this exact Lebanese adaptation should be checked on PHQ Screeners. No separate licence fee is stated by Pfizer.	Floor and ceiling effects were NR.	Of 100 eligible/approached participants, 51 men participated (51%); item-level missingness was NR. Twenty completed the retest.	Mean PHQ-9-A 7.2 (*SD* 5.2). ROC *AUC*=0.83 (*95% CI* 0.72-0.94); cutoff 7 gave sensitivity 83% and specificity 69% against HDRS-A cutoff 7.5. Conventional PHQ severity thresholds 5, 10, 15, and 20 and a previously validated Lebanese cutoff 10 were also reported.	NR
Patient Health Questionnaire-2 (English)<br>Original two-item form corresponding to PHQ-9 items 1 and 2	2	Derived from PHQ-9 items 1 and 2 in Graves and Bombardier and Bombardier et al.; independently administered in written or oral format in Poritz et al. Completion time NR.	**Included reports:** The two source items were reproduced within PHQ-9 item tables. Exact licensing terms, cost, and translations were NR.<br>**Current access (Sep 2026):** Pfizer specifically lists the PHQ-2 among PHQ variants available through PHQ Screeners. It can be downloaded after accepting the terms of use without a formal permission request; no separate licence fee or account-registration requirement is stated for this route. Approved translations are available. (Pfizer/PHQ Screeners)	Floor and ceiling effects were NR.	Graves and Bombardier: 3,652 complete PHQ-9 records. Bombardier et al.: 142 paired PHQ-2/SCID assessments. Poritz et al.: 116/144 completed both measures. Item-level missingness was NR.	Against SCID MDD, no PHQ-2 cutoff achieved both sensitivity and specificity >0.80: >=1, 1.00/0.48; >=2, 0.93/0.67; >=3, 0.57/0.84. Poritz et al. reported *AUC*=0.979 against PHQ-9>=10, but PHQ-9 was not an independent diagnostic gold standard. The evidence supports PHQ-2 only as a first-stage prescreen.	NR
PHQ-9-derived 3-item short form (English)<br>Post hoc three-item form comprising PHQ-9 items 1, 2 and 6	3	Post hoc score derived from complete PHQ-9 responses; not independently administered. Completion time NR.	**Included reports:** Source items 1, 2, and 6 were reproduced. Exact licensing terms, cost, and translations were NR.<br>**Current access (Sep 2026):** The source PHQ items follow Pfizer/PHQ Screeners terms, but this three-item score is a post hoc research-derived form and is not a separately distributed official PHQ instrument. Access to the parent items does not establish independent validation or separate licensing status for the derived score.	Floor and ceiling effects were NR.	All 3,652 analysed records had complete PHQ-9 data; the number excluded for incomplete PHQ-9 data was NR.	Relative efficiency versus PHQ-9 was reported as 0.67 in the results and 0.66 in the abstract/conclusion. Against parent PHQ-9>=10, cutoff >=3 had sensitivity 0.87 and specificity 0.93; cutoff >=4 had sensitivity 0.82 and specificity 0.95. These are parent-scale agreement data, not independent diagnostic criterion validity.	NR

PROM and version/form	Items, *n*	Administration burden and mode	Accessibility, licensing, registration, cost, and translations	Floor/ceiling effects	Missing data/completeness	Reference scores and cut-offs	MIC/ MID
SCL-90-R Cognitive/Affective Depression Research Subscale (English) Experimental 13-item cognitive/affective depression research subscale	13	Completed during the initial SCI rehabilitation evaluation; exact written/oral mode and completion time were NR.	**Included reports:** All 13 item stems were reproduced, but full administration/scoring instructions were not. Exact licensing terms, cost, and translations were NR. **Current access (Sep 2026):** Pearson distributes the parent SCL-90-R as a proprietary Qualification Level B assessment; test materials and report usages are purchased and qualification/account requirements apply. This experimental research subscale is not separately marketed, and the underlying SCL-90-R item rights remain proprietary. (Pearson Assessments)	Floor and ceiling effects were NR.	SCI sample *n*=52; item-level missingness and exclusions were NR.	SCI *α*=0.89. The exact experimental-subscale mean was not tabulated; the standard SCL-90-R Depression T score in the SCI group was 61.7. No diagnostic or severity cutoff was reported.	NR
SCL-90-R Somatic Depression Research Subscale (English) Experimental 7-item somatic depression research subscale	7	Completed during the initial SCI rehabilitation evaluation; exact written/oral mode and completion time were NR.	**Included reports:** All seven item stems were reproduced, but full administration/scoring instructions were not. Exact licensing terms, cost, and translations were NR. **Current access (Sep 2026):** Pearson distributes the parent SCL-90-R as a proprietary Qualification Level B assessment; test materials and report usages are purchased and qualification/account requirements apply. This experimental research subscale is not separately marketed, and the underlying SCL-90-R item rights remain proprietary. (Pearson Assessments)	Floor and ceiling effects were NR.	SCI sample *n*=52; item-level missingness and exclusions were NR.	SCI *α*=0.62. The exact subscale mean was not tabulated. Item 60 (overeating) had the weakest SCI item-total correlation (*r*=0.25), but no validated six-item version was reported. No diagnostic or severity cutoff was reported.	NR
Inventory to Diagnose Depression total severity score (English) Original 22-item self-report IDD total severity score	22	Self-completed when possible; assistance was provided for physical limitations. Completion time NR.	**Included reports:** Only abbreviated symptom labels were reported, not the full instrument. Licensing, cost, and translations were NR. **Current access (Sep 2026):** No current authoritative instrument-owner distribution/licensing or registration page was identified for the IDD version evaluated here. Access to the original publication does not establish free instrument reuse; free routine clinical use should therefore not be assumed without confirmation from the relevant rights holder/publisher.	Floor and ceiling effects were NR.	SCI group *n*=134; age data were available for *n*=132. Item-level missingness and the denominator for the reported MDD percentage were NR.	SCI mean total severity 13.3. The report stated 13 participants (11%) met DSM-III-R MDD criteria; 13/134 is not 11%, so the article-reported count and percentage are internally inconsistent. The embedded DSM-III-R algorithm was used; the exact numerical threshold was NR.	NR
Inventory to Diagnose Depression Dysphoria factor score (English) Post hoc nine-item dysphoria factor score	9	Post hoc score derived from the same assisted/self-completed IDD administration; completion time NR.	**Included reports:** Only abbreviated symptom labels were reported. Licensing, cost, and translations were NR. **Current access (Sep 2026):** No current authoritative licensing route was identified. The dysphoria score is a post hoc factor score rather than a separately distributed instrument; free routine clinical reuse and registration requirements are not established and should not be inferred from publication access.	Floor and ceiling effects were NR.	SCI group *n*=134; factor-score missingness was NR.	Nine-item post hoc dysphoria score. Mean scores: SCI 5.30, rheumatoid arthritis 7.17, students 6.10, and community 4.74. Exact score range and cutoff were NR.	NR
Brief Symptom Inventory SCI-specific Depression Research Factor (7-item English) SCI-specific seven-item Depression research factor	7	Full BSI administered orally by a psychologist or social worker during initial inpatient rehabilitation; completion time NR.	**Included reports:** Seven item stems were listed, but full BSI instructions and response options were not reproduced. Exact licensing terms, cost, and translations were NR. **Current access (Sep 2026):** The parent BSI is a proprietary Pearson Qualification Level B assessment with purchased materials/report usages and qualification/account requirements. This SCI-specific seven-item factor is research-derived and not separately marketed; underlying BSI item rights remain proprietary. (Pearson Assessments)	Floor and ceiling effects were NR.	Factor-analysis *n*=215; rehospitalization follow-up *n*=204, with 11 missing follow-up. Item-level missingness was NR.	Mean factor score 0.58 (*SD* 0.73); *α*=0.88. The published high-score cutoff was 1.17, but the stated rule was mean+1.5 *SD*, which gives approximately 1.68. The reported cutoff was retained as an unresolved article inconsistency and should not be treated as validated.	NR

Table SIV. COSMIN risk-of-bias ratings for individual measurement-property results

Notes: RoB, risk of bias; V, very good; A, adequate; D, doubtful; I, inadequate; NA, not applicable; NR, not reported. Ratings are reported at the individual analysis-result level. When one report contributed more than one analysis to the same measurement property, the analyses are shown separately; the summarized study-level table uses the lowest applicable rating. Accordingly, Tulsky et al. contributed an adequate CFA analysis and a doubtful IRT calibration analysis, and Graves and Bombardier contributed a very-good CFA analysis and a doubtful IRT analysis. The two pooled Buckelew et al. correlation analyses were not SCI-specific and were not rated or included in the SCI-specific synthesis. The SCI-QOL Depression Short Form 10a was derived from the item bank but was not independently evaluated for a COSMIN measurement property.

PROM and version/form	Report or analysis	Measurement property or study component	Analysis sample, *n*	Consensus COSMIN RoB rating
CES-D-20 (English)	McColl & Skinner, 1992	Structural validity	120	A
CES-D-20 (English)	McColl & Skinner, 1992	Internal consistency	120	V
CES-D-20 (English)	Miller et al., 2008	Internal consistency	47	V
CES-D-20 (English)	Cook et al., 2017	Cross-cultural validity/measurement invariance	SCI 238; reference 726; total 964	I
CES-D-20 (English)	Miller et al., 2008	Reliability	47	A
CES-D-20 (English)	Miller et al., 2008	Measurement error	47	A
CES-D-20 (English)	McColl & Skinner, 1992	Hypotheses testing for construct validity	120	I
CES-D-20 (English)	Miller et al., 2008	Hypotheses testing for construct validity	47	I
CES-D-20 (English)	Miller et al., 2008	Hypotheses testing for construct validity	47	I
Zung Self-Rating Depression Scale (English)	Tate et al., 1993	Structural validity	162	D
Zung Self-Rating Depression Scale (English)	Tate et al., 1993	Internal consistency	162	V
Zung Self-Rating Depression Scale (English)	Tate et al., 1993	Criterion validity	30	D
Zung Self-Rating Depression Scale (English)	Campagnolo et al., 2002	Hypotheses testing for construct validity	39	D
Zung Self-Rating Depression Scale (English)	Campagnolo et al., 2002	Hypotheses testing for construct validity	59	A
Zung Self-Rating Depression Scale (English)	Tate et al., 1993	Hypotheses testing for construct validity	162	I
Brief Symptom Inventory – Depression subscale (English)	Heinrich & Tate, 1996	Structural validity	215	I
Brief Symptom Inventory – Depression subscale (English)	Tate et al., 1993	Structural validity	162	I
Brief Symptom Inventory – Depression subscale (English)	Tate et al., 1993	Internal consistency	162	V
Brief Symptom Inventory – Depression subscale (English)	Mitchell et al., 2008	Criterion validity	40	A
Brief Symptom Inventory – Depression subscale (English)	Tate et al., 1993	Criterion validity	30	D
Brief Symptom Inventory – Depression subscale (English)	Heinrich & Tate, 1996	Hypotheses testing for construct validity	Follow-up *n*=204	D
Brief Symptom Inventory – Depression subscale (English)	Mitchell et al., 2008	Hypotheses testing for construct validity	40	D
Brief Symptom Inventory – Depression subscale (English)	Tate et al., 1990	Hypotheses testing for construct validity	SCI 79; normative 974; paired subset 20	I
Brief Symptom Inventory – Depression subscale (English)	Tate et al., 1993	Hypotheses testing for construct validity	162	I
Beck Depression Inventory-I (English)	Radnitz et al., 1997	Internal consistency	124	V
Beck Depression Inventory-I (English)	Radnitz et al., 1997	Criterion validity	124	A
Ilfeld Psychiatric Symptom Index - Depression subscale (English)	Campagnolo et al., 2002	Hypotheses testing for construct validity	39	I
Ilfeld Psychiatric Symptom Index - Depression subscale (English)	Campagnolo et al., 2002	Hypotheses testing for construct validity	59	A
Patient Health Questionnaire-9 (English)	Graves & Bombardier, 2008 - graded-response-model and subgroup-functioning analyses	Structural validity	3652	D
Patient Health Questionnaire-9 (English)	Graves & Bombardier, 2008 - one-factor	Structural validity	3652	V

PROM and version/form	Report or analysis	Measurement property or study component	Analysis sample, *n*	Consensus COSMIN RoB rating
	CFA			
Patient Health Questionnaire-9 (English)	Kalpakjian et al., 2009	Structural validity	RS1/RS2 *n*=584 each; sex-specific analyses *n*=292 each	V
Patient Health Questionnaire-9 (English)	Krause et al., 2008	Structural validity	568	V
Patient Health Questionnaire-9 (English)	Krause et al., 2010 - first follow-up EFA	Structural validity	211	D
Patient Health Questionnaire-9 (English)	Krause et al., 2010 - second follow-up CFA/EFA	Structural validity	302	D
Patient Health Questionnaire-9 (English)	Krause et al., 2011 - full-sample CFA	Structural validity	7296	V
Patient Health Questionnaire-9 (English)	Richardson & Richards, 2008	Structural validity	2570	V
Patient Health Questionnaire-9 (English)	Williams et al., 2009 - CTT factor analysis	Structural validity	EFA *n*=91; CFA *n*=111	V
Patient Health Questionnaire-9 (English)	Williams et al., 2009 - Rasch analysis	Structural validity	202	V
Patient Health Questionnaire-9 (English)	Williams et al., 2016 - original scoring	Structural validity	133 participants / 884 observations	I
Patient Health Questionnaire-9 (English)	Bombardier et al., 2004	Internal consistency	849	V
Patient Health Questionnaire-9 (English)	Kalpakjian et al., 2009	Internal consistency	RS1/RS2 *n*=584 each; sex-specific analyses *n*=292 each	V
Patient Health Questionnaire-9 (English)	Krause et al., 2008	Internal consistency	568	V
Patient Health Questionnaire-9 (English)	Krause et al., 2009	Internal consistency	NR (classification *n*=690)	V
Patient Health Questionnaire-9 (English)	Poritz et al., 2018	Internal consistency	116	V
Patient Health Questionnaire-9 (English)	Richardson & Richards, 2008	Internal consistency	2570	V
Patient Health Questionnaire-9 (English)	Williams et al., 2009 - Rasch analysis	Internal consistency	202	V
Patient Health Questionnaire-9 (English)	Williams et al., 2016 - original scoring	Internal consistency	133 participants / 884 observations	I
Patient Health Questionnaire-9 (English)	Cook et al., 2017	Cross-cultural validity/measurement invariance	SCI 3,694; reference 3,000; total 6,694	I
Patient Health Questionnaire-9 (English)	Graves & Bombardier, 2008 - graded-response-model and subgroup-functioning analyses	Cross-cultural validity/measurement invariance	3652	I
Patient Health Questionnaire-9 (English)	Graves & Bombardier, 2008 - one-factor CFA	Cross-cultural validity/measurement invariance	Men 2,863; women 789	I
Patient Health Questionnaire-9 (English)	Kalpakjian et al., 2009	Cross-cultural validity/measurement invariance	1,168 total; 292 women and 292 men in each random sample	I
Patient Health Questionnaire-9 (English)	Krause et al., 2010 - first follow-up EFA	Cross-cultural validity/measurement invariance	554, 211, and 302	I
Patient Health Questionnaire-9 (English)	Krause et al., 2011 - full-sample CFA	Cross-cultural validity/measurement invariance	582-5,767 across strata	I
Patient Health Questionnaire-9 (English)	Richardson & Richards, 2008	Cross-cultural validity/measurement invariance	2570	I
Patient Health Questionnaire-9 (English)	Bombardier et al., 2012	Criterion validity	142	A
Patient Health Questionnaire-9 (English)	Bombardier et al., 2004	Hypotheses testing for construct validity	144	D
Patient Health Questionnaire-9 (English)	Bombardier et al., 2004	Hypotheses testing for construct validity	638	D
Patient Health Questionnaire-9 (English)	Bombardier et al., 2004	Hypotheses testing for construct validity	849	A
Patient Health Questionnaire-9 (English)	Bombardier et al., 2012	Hypotheses testing for construct validity	Maximum 142; role-difficulty *n* NR	D
Patient Health Questionnaire-9 (English)	Krause et al., 2009	Hypotheses testing for construct validity	Reported as *r*(655)	D
Patient Health Questionnaire-9 (English)	Krause et al., 2009	Hypotheses testing for construct validity	Reported as *r*(678)	D

PROM and version/form	Report or analysis	Measurement property or study component	Analysis sample, *n*	Consensus COSMIN RoB rating
Patient Health Questionnaire-9 (English)	Richardson & Richards, 2008	Hypotheses testing for construct validity	670, 509, 640, and 709	D
Patient Health Questionnaire-9 (English)	Tulsky et al., 2015 - SCI-QOL Depression comparison	Hypotheses testing for construct validity	465	A
Hospital Anxiety and Depression Scale - Depression subscale (English)	Woolrich et al., 2006	Structural validity	963	D
Hospital Anxiety and Depression Scale - Depression subscale (English)	Woolrich et al., 2006	Internal consistency	963	V
Hospital Anxiety and Depression Scale - Depression subscale (English)	Woolrich et al., 2006	Cross-cultural validity/measurement invariance	963	I
Hospital Anxiety and Depression Scale - Depression subscale (English)	Woolrich et al., 2006	Hypotheses testing for construct validity	NR (maximum 963)	D
CES-D-10 (English)	Miller et al., 2008	Internal consistency	47	V
CES-D-10 (English)	Miller et al., 2008	Reliability	47	A
CES-D-10 (English)	Miller et al., 2008	Measurement error	47	A
CES-D-10 (English)	Miller et al., 2008	Hypotheses testing for construct validity	47	I
CES-D-10 (English)	Miller et al., 2008	Hypotheses testing for construct validity	47	I
Depression Anxiety Stress Scales-21 - Depression subscale (English)	Mitchell et al., 2008	Criterion validity	40	A
Depression Anxiety Stress Scales-21 - Depression subscale (English)	Mitchell et al., 2008	Hypotheses testing for construct validity	40	I
Older Adult Health and Mood Questionnaire (English)	Krause et al., 2009	Internal consistency	NR (classification *n*=682)	V
Older Adult Health and Mood Questionnaire (English)	Krause et al., 2009	Hypotheses testing for construct validity	Reported as *r*(655)	I
Older Adult Health and Mood Questionnaire (English)	Krause et al., 2009	Hypotheses testing for construct validity	Reported as *r*(671)	D
Hospital Anxiety and Depression Scale - Depression subscale (German)	Müller et al., 2012	Structural validity	100	D
Hospital Anxiety and Depression Scale - Depression subscale (German)	Müller et al., 2012	Internal consistency	100	V
Hospital Anxiety and Depression Scale - Depression subscale (German)	Müller et al., 2012	Cross-cultural validity/measurement invariance	100	I
Hospital Anxiety and Depression Scale - Depression subscale (Turkish)	Paker et al., 2013	Internal consistency	175	V
SCI-QOL Depression Item Bank (English)	Tulsky et al., 2015	PROM development - concept elicitation	NR	D
SCI-QOL Depression Item Bank (English)	Tulsky et al., 2015	PROM development - cognitive interviews/pilot testing	NR	D
SCI-QOL Depression Item Bank (English)	Tulsky et al., 2015	Content validity - overall PROM-development evidence	NR	D
SCI-QOL Depression Item Bank (English)	Tulsky et al., 2015 - CFA, internal consistency, DIF, and PHQ-9 comparison	Structural validity	716	A
SCI-QOL Depression Item Bank (English)	Tulsky et al., 2015 - graded-response IRT calibration	Structural validity	716	D
SCI-QOL Depression Item Bank (English)	Tulsky et al., 2015 - CFA, internal consistency, DIF, and PHQ-9 comparison	Internal consistency	716	V
SCI-QOL Depression Item Bank (English)	Tulsky et al., 2015 - CFA, internal consistency, DIF, and PHQ-9 comparison	Cross-cultural validity/measurement invariance	716	D
SCI-QOL Depression Item Bank (English)	Tulsky et al., 2015 - CFA, internal consistency, DIF, and PHQ-9 comparison	Hypotheses testing for construct validity	465	I
SCI-QOL Depression CAT (English)	Tulsky et al., 2015 - 1-2 week test-retest reliability	Reliability	245	D

**PROM and version/form**	**Report or analysis**	**Measurement property or study component**	**Analysis sample, *n***	**Consensus COSMIN RoB rating**
SCI-QOL Depression Short Form 10a (English)	Tulsky et al., 2015	No independent measurement-property evaluation	NA	NA
Patient Health Questionnaire-9, revised 3-category scoring (English)	Williams et al., 2016 - post hoc revised scoring	Structural validity	133 participants / 884 observations	I
Patient Health Questionnaire-9, revised 3-category scoring (English)	Williams et al., 2016 - post hoc revised scoring	Internal consistency	133 participants / 884 observations	I
Patient Health Questionnaire-9, revised 3-category scoring (English)	Williams et al., 2016 - post hoc revised scoring	Criterion validity	Exact week-12 *n* NR (maximum 133)	D
Patient Health Questionnaire-9, revised 3-category scoring (English)	Williams et al., 2016 - post hoc revised scoring	Hypotheses testing for construct validity	Exact week-12 *n* NR (maximum 133)	I
Patient Health Questionnaire-9, revised 3-category scoring (English)	Williams et al., 2016 - post hoc revised scoring	Responsiveness	133 participants / 884 observations	I
Hopkins Symptom Checklist-20 Depression Scale, Version B (English)	Williams et al., 2016 - original scoring	Structural validity	133 participants / 885 observations	I
Hopkins Symptom Checklist-20 Depression Scale, Version B (English)	Williams et al., 2016 - original scoring	Internal consistency	133 participants / 885 observations	I
Hopkins Symptom Checklist revised 12-item depression scale (English)	Williams et al., 2016 - post hoc revised scoring	Structural validity	133 participants / 885 observations	I
Hopkins Symptom Checklist revised 12-item depression scale (English)	Williams et al., 2016 - post hoc revised scoring	Internal consistency	133 participants / 885 observations	I
Hopkins Symptom Checklist revised 12-item depression scale (English)	Williams et al., 2016 - post hoc revised scoring	Criterion validity	Exact week-12 *n* NR (maximum 133)	I
Hopkins Symptom Checklist revised 12-item depression scale (English)	Williams et al., 2016 - post hoc revised scoring	Hypotheses testing for construct validity	Exact week-12 *n* NR (maximum 133)	I
Hopkins Symptom Checklist revised 12-item depression scale (English)	Williams et al., 2016 - post hoc revised scoring	Responsiveness	133 participants / 885 observations	I
PROMIS Depression Short Form 8b, version 1.0 (English)	Cook et al., 2017	Cross-cultural validity/measurement invariance	SCI 292; reference 761; total 1,053	I
Patient Health Questionnaire-9, Arabic version (Lebanese Arabic)	Summaka et al., 2019	Content validity - patient-rated comprehensibility	15	D
Patient Health Questionnaire-9, Arabic version (Lebanese Arabic)	Summaka et al., 2019 - factor analysis, internal consistency, retest reliability, ROC and convergent validity	Structural validity	51	D
Patient Health Questionnaire-9, Arabic version (Lebanese Arabic)	Summaka et al., 2019 - factor analysis, internal consistency, retest reliability, ROC and convergent validity	Internal consistency	51	A
Patient Health Questionnaire-9, Arabic version (Lebanese Arabic)	Summaka et al., 2019 - factor analysis, internal consistency, retest reliability, ROC and convergent validity	Reliability	20	D
Patient Health Questionnaire-9, Arabic version (Lebanese Arabic)	Summaka et al., 2019 - factor analysis, internal consistency, retest reliability, ROC and convergent validity	Criterion validity	51	I
Patient Health Questionnaire-9, Arabic version (Lebanese Arabic)	Summaka et al., 2019 - HDRS-A-defined known groups	Hypotheses testing for construct validity	51	D
Patient Health Questionnaire-9, Arabic version (Lebanese Arabic)	Summaka et al., 2019 - factor analysis, internal consistency, retest reliability, ROC and convergent validity	Hypotheses testing for construct validity	51	A
Patient Health Questionnaire-2 (English)	Graves & Bombardier, 2008	Structural validity	3652	D
Patient Health Questionnaire-2 (English)	Poritz et al., 2018	Internal consistency	116	V
Patient Health Questionnaire-2 (English)	Bombardier et al., 2012	Criterion validity	142	A
Patient Health Questionnaire-2 (English)	Poritz et al., 2018	Criterion validity	116	I
PHQ-9-derived 3-item short form (English)	Graves & Bombardier, 2008	Structural validity	3652	D
PHQ-9-derived 3-item short form (English)	Graves & Bombardier, 2008	Cross-cultural validity/measurement invariance	Men 2,863; women 789	I
PHQ-9-derived 3-item short form (English)	Graves & Bombardier, 2008	Criterion validity	3652	I
SCL-90-R Cognitive/Affective Depression	Buckelew et al., 1988 - SCI internal	Internal consistency	52	V

**PROM and version/form**	**Report or analysis**	**Measurement property or study component**	**Analysis sample, *n***	**Consensus COSMIN RoB rating**
Research Subscale (English)	consistency and known-groups validity			
SCL-90-R Cognitive/Affective Depression Research Subscale (English)	Buckelew et al., 1988 - SCI internal consistency and known-groups validity	Hypotheses testing for construct validity	SCI 52; total 275	D
SCL-90-R Cognitive/Affective Depression Research Subscale (English)	Buckelew et al., 1988 - pooled convergent/divergent correlations; not SCI-specific	Hypotheses testing for construct validity	Pooled *n*=275; SCI-specific unavailable	NA
SCL-90-R Somatic Depression Research Subscale (English)	Buckelew et al., 1988 - SCI internal consistency and known-groups validity	Internal consistency	52	V
SCL-90-R Somatic Depression Research Subscale (English)	Buckelew et al., 1988 - SCI internal consistency and known-groups validity	Hypotheses testing for construct validity	SCI 52; total 275	D
SCL-90-R Somatic Depression Research Subscale (English)	Buckelew et al., 1988 - pooled convergent/divergent correlations; not SCI-specific	Hypotheses testing for construct validity	Pooled *n*=275; SCI-specific unavailable	NA
Inventory to Diagnose Depression total severity score (English)	Frank et al., 1992	Structural validity	134	A
Inventory to Diagnose Depression total severity score (English)	Frank et al., 1992	Cross-cultural validity/measurement invariance	Total 502; SCI 134	D
Inventory to Diagnose Depression total severity score (English)	Frank et al., 1992	Hypotheses testing for construct validity	SCI recruitment *n*=134; the four-group ANOVA reported *df*=3,452, implying total analyzable *n*=456. Group-specific complete-case denominators were not reported.	D
Inventory to Diagnose Depression Dysphoria factor score (English)	Frank et al., 1992	Structural validity	134	D
Inventory to Diagnose Depression Dysphoria factor score (English)	Frank et al., 1992	Cross-cultural validity/measurement invariance	Total 502; SCI 134	D
Inventory to Diagnose Depression Dysphoria factor score (English)	Frank et al., 1992	Hypotheses testing for construct validity	SCI recruitment *n*=134; the four-group ANOVA reported *df*=3,452, implying total analyzable *n*=456. Group-specific complete-case denominators were not reported.	D
Brief Symptom Inventory SCI-specific Depression Research Factor (7-item English)	Heinrich & Tate, 1996	Structural validity	215	I
Brief Symptom Inventory SCI-specific Depression Research Factor (7-item English)	Heinrich & Tate, 1996	Internal consistency	215	V
Brief Symptom Inventory SCI-specific Depression Research Factor (7-item English)	Heinrich & Tate, 1996	Hypotheses testing for construct validity	Follow-up *n*=204	D

Table SV. Individual measurement-property results and COSMIN ratings

Notes: PROM, patient-reported outcome measure; RoB, risk of bias; V, very good; A, adequate; D, doubtful; I, inadequate; NA, not applicable; NR, not reported. COSMIN ratings: +, sufficient; -, insufficient; ?, indeterminate. Results are presented at the individual analysis-result level. Different language versions, scoring systems, short forms, item banks, CATs, and research-derived factors were treated as separate PROMs. Rows with RoB and COSMIN rating NA were not included in the SCI- specific synthesis. The SCI-QOL Depression Short Form 10a was derived from the calibrated item bank but was not independently evaluated. Published internal inconsistencies were retained and described rather than resolved by assumption.

PROM and version/form	Report or analysis	Measurement property or study component	Analysis sample, *n*	Reported result	COSMIN rating	RoB rating
CES-D-20 (English)	McColl & Skinner, 1992	Structural validity	120	Time 1, *n*=120. PCA first five eigenvalues 7.91, 1.53, 1.20, 1.10, 0.98; first factor explained 39.6%. One-factor LISREL CFA using polychoric correlations/ULS: RMSR=0.103, CD=0.951, AGFI=0.942. Current COSMIN v2 sufficient structural-validity criteria were not met.	-	A
CES-D-20 (English)	McColl & Skinner, 1992	Internal consistency	120	Time 1, *n*=120: Cronbach's *alpha*=0.906. Because sufficient structural validity was not established, internal consistency is indeterminate under COSMIN v2.	?	V
CES-D-20 (English)	Miller et al., 2008	Internal consistency	47	Miller et al. (2008), *n*=47: Cronbach's *alpha*=0.91. Deleting any individual item changed *alpha* by no more than 0.05. No new structural-validity analysis was conducted.	?	V
CES-D-20 (English)	Cook et al., 2017	Cross-cultural validity/measurement invariance	SCI 238; reference 726; total 964	Diagnosis-related DIF was evaluated by lordif ordinal logistic regression with a graded response model in SCI *n*=238 versus PROMIS community *n*=726. Combined-sample assumption checks were: *RMSEA*=0.103, *CFI*=0.925, *TLI*=0.925; EFA first-factor variance=57.7%, first/second eigenvalue ratio=7.8, factor correlation=0.657; 2/160 residual item pairs exceeded 0.20. No CES-D item met the study's standard meaningful-DIF criteria (>10% or >5% *beta* change, or McFadden delta-R2>0.05). At the deliberately sensitive delta-R2>=0.005 threshold, items 1, 2, 3, 6, 7, 10, 15, 17 and 19 were flagged; items 6 and 10 showed non-uniform DIF and the remainder uniform DIF. DIF-corrected and uncorrected SCI scores correlated 0.998; difference range -3 to 2, mean 0.0258, *SD* 0.74812. Exact item-level McFadden delta-R2 values were not reported, so it cannot be verified that all were <0.02 as required by COSMIN; the result was rated indeterminate.	?	I
CES-D-20 (English)	Miller et al., 2008	Reliability	47	Two-week test–retest reliability, *n*=47: *ICC*(1,1)=0.87 (*95% CI* 0.79–0.93). Item-level *ICC*(1,1), *95% CI*: item 1 0.32 (0.04–0.55); 2 0.51 (0.27–0.69); 3 0.73 (0.56–0.84); 4 0.11 (0.01–0.38); 5 0.62 (0.41–0.77); 6 0.67 (0.48–0.80); 7 0.59 (0.37–0.75); 8 0.59 (0.37–0.75); 9 0.28 (0.03–0.52); 10 0.47 (0.22–0.67); 11 0.64 (0.43–0.78); 12 0.52 (0.28–0.70); 13 0.64 (0.43–0.78); 14 0.55 (0.32–0.72); 15 0.43 (0.17–0.64); 16 0.63 (0.41–0.77); 17 0.54 (0.30–0.71); 18 0.62 (0.41–0.77); 19 0.71 (0.53–0.83); 20 0.68 (0.49–0.81). The Results text states the lowest item *ICC* was 0.30 for item 4, but The tabulated result reports 0.11; the table value was retained.	+	A
CES-D-20 (English)	Miller et al., 2008	Measurement error	47	Bland–Altman analysis indicated minimal systematic bias. Session 1 minus session 2 mean difference=-1.30 (*95% CI* -2.76 to 0.24; *t*=-1.70, *p*=0.10). The article reports that three of 47 points lay outside ±2 *SD* but does not clearly assign that count to a specific CES-D version. The authors stated that <15% scored at either scale extreme. Numeric LoA and an MIC were not reported.	?	A
CES-D-20 (English)	McColl & Skinner, 1992	Hypotheses testing for construct validity	120	Latent correlation between CES-D depression and the adapted SIP adjustment-to-disability factor: *r*=0.659; 1/1 reconstructed hypothesis for related-but-	+	I

PROM and version/form	Report or analysis	Measurement property or study component	Analysis sample, *n*	Reported result	COSMIN rating	RoB rating
				distinct constructs was supported.		
CES-D-20 (English)	Miller et al., 2008	Hypotheses testing for construct validity	47	A priori convergent-validity result, *n*=47: CES-D-20 correlated positively with VAS-*F* at *r*=0.52. The hypothesis *r*>0.50 was supported.	+	I
CES-D-20 (English)	Miller et al., 2008	Hypotheses testing for construct validity	47	A priori SF-36 correlations, *n*=47: mental health 0.75, emotional role 0.55, vitality 0.54, pain 0.27, social role 0.37, physical function 0.34, physical role 0.40, and general health 0.57. The published coefficients are printed positive signs, while the Methods, Results, and Discussion explicitly state that the expected negative direction was observed. Because the exact signed coefficients are internally inconsistent, this result set was rated indeterminate.	?	I
Zung Self-Rating Depression Scale (English)	Tate et al., 1993	Structural validity	162	Outpatients *n*=162. PCA with varimax rotation forced two factors to examine the established affective/somatic structure. The two factors explained 35% of total variance and largely corresponded to affective and psychosomatic dimensions, but did not completely replicate the published structure. Only 12 items with loadings ≥0.50 were displayed. COSMIN sufficient structural-validity criteria were not met.	-	D
Zung Self-Rating Depression Scale (English)	Tate et al., 1993	Internal consistency	162	Outpatients *n*=162: Cronbach's *alpha*=0.81 for the Zung total score. Because sufficient unidimensionality/structural validity was not established, the coefficient is indeterminate under COSMIN v2.	?	V
Zung Self-Rating Depression Scale (English)	Tate et al., 1993	Criterion validity	30	Inpatients *n*=30; concordant yes/no depression classification by two rehabilitation clinicians, guided by DSM-III-R symptoms (clinician kappa=0.63; 84% agreement). Zung cut-off >55: sensitivity 86%, specificity 61%, total agreement 67%, kappa=0.33 (*p*<0.01). No *AUC* or correlation with a structured diagnostic criterion was reported; result rated indeterminate.	?	D
Zung Self-Rating Depression Scale (English)	Campagnolo et al., 2002	Hypotheses testing for construct validity	39	Campagnolo et al. (2002), chronic SCI *n*=39. Zung total correlated with the Ilfeld PSI depression subscale *r*=0.78 (*p*<0.01), Ilfeld total *r*=0.72, anxiety *r*=0.59, and cognitive disturbance *r*=0.34. The depression-specific correlation was the strongest, as hypothesised.	+	D
Zung Self-Rating Depression Scale (English)	Campagnolo et al., 2002	Hypotheses testing for construct validity	59	Known-groups data, *n*=59: Zung means were 35.2 (*SD* 6.4) for tetraplegia, 38.2 (9.3) for paraplegia, and 29.4 (6.7) for controls; both SCI subgroup means exceeded the control mean by >10%. Overall ANOVA *F*(2,56)=6.87, *p*=0.002; Tukey testing was significant only for paraplegia versus controls (*p*=0.002). *AUC*=0.76 discriminated SCI from control status; this was treated as known-groups evidence, not criterion validity, because SCI status is not a depression gold standard.	+	A
Zung Self-Rating Depression Scale (English)	Tate et al., 1993	Hypotheses testing for construct validity	162	Outpatients *n*=162. Zung total correlated with BSI/GSI *r*=0.53 and BSI-D *r*=0.52; correlations with other BSI dimensions ranged 0.34–0.51. Zung somatic correlations ranged 0.24–0.43 (BSI-D *r*=0.37); Zung affective correlations ranged 0.27–0.54 (BSI-D *r*=0.54). Dichotomized agreement: Zung vs BSI-D kappa=0.59, 85% agreement; vs BSI/GSI kappa=0.44, 78% agreement. All 5 prespecified/reconstructable Zung hypotheses were supported.	+	I
Brief Symptom Inventory – Depression subscale (English)	Heinrich & Tate, 1996	Structural validity	215	All six standard BSI Depression items loaded together on the SCI Depression factor (loadings .50-.74), but the factor also included psychoticism item 14, and maximum-likelihood estimation split depression into two subscales. No CFA	?	I

PROM and version/form	Report or analysis	Measurement property or study component	Analysis sample, *n*	Reported result	COSMIN rating	RoB rating
				or separate six-item dimensionality test was performed. The standard BSI Depression structure was therefore rated indeterminate. Principal-components and maximum-likelihood exploratory analyses were run on all 53 BSI items (*n*=215; 4.06 participants/item) with oblique rotation. Models with 3-14 factors were examined. Factors were retained using substantive interpretability, >=5% variance, >=5 items and loading >=.35. The principal-components solution identified seven factors explaining 52.5% of variance; 45/53 items loaded >=.35. A general-somatization factor had *alpha*=.64 and was dropped, leaving six recommended research factors. The unrotated first factor explained 27%. Depression accounted for 9.6% and *alpha*=.88. Maximum-likelihood estimation gave a less stable depression solution, splitting depression into two subscales. Factor intercorrelations for Depression were .36 with Anxiety, .24 with Mental Blocks, .23 with Interpersonal Sensitivity, .13 with SCI and .25 with Hostile Suspiciousness. Seven-item Depression research factor: item 35 feeling hopeless about the future loading .74; item 18 feeling no interest in things .68; item 9 thoughts of ending your life .64; item 17 feeling blue .61; item 14 feeling lonely even when with people .60; item 16 feeling lonely .56; item 50 feelings of worthlessness .50.		
Brief Symptom Inventory – Depression subscale (English)	Tate et al., 1993	Structural validity	162	Outpatients *n*=162. PCA with varimax rotation of all 53 BSI items attempted to reproduce the expected nine-factor structure; the original dimensions were not reproduced and none of the resultant scales represented a distinct construct. When three factors were forced, only one represented a single conceptual dimension. The factor analysis tested 53 items with *n*=162 (<5 participants/item). COSMIN sufficient structural-validity criteria were not met.	-	I
Brief Symptom Inventory – Depression subscale (English)	Tate et al., 1993	Internal consistency	162	Outpatients *n*=162: Cronbach's *alpha*=0.87 for the BSI Depression subscale. Because sufficient structural validity of the depression dimension was not established, the coefficient is indeterminate under COSMIN v2.	?	V
Brief Symptom Inventory – Depression subscale (English)	Mitchell et al., 2008	Criterion validity	40	Reference standard: MINI English Version 5.0.0 structured diagnostic interview, administered by an onsite clinical psychologist blinded to questionnaire results. Seven of 40 participants had a depression diagnosis (4 major depressive episodes and 3 dysthymia). For the traditional BSI-D cutoff, sensitivity was 57% and specificity 82%; for the SCI-specific elevated cutoff, sensitivity was 14% and specificity 97%. *AUC* and correlation with the diagnostic criterion were not reported, so the result is indeterminate under the review's COSMIN rule.	?	A
Brief Symptom Inventory – Depression subscale (English)	Tate et al., 1993	Criterion validity	30	Inpatients *n*=30; concordant clinician depression classification guided by DSM-III-R symptoms. BSI-D T-score cut-off ≥65: sensitivity 57%, specificity 87%, total agreement 80%, kappa=0.44 (*p*<0.01). No *AUC* or correlation with a structured diagnostic criterion was reported; result rated indeterminate.	?	D
Brief Symptom Inventory – Depression subscale (English)	Heinrich & Tate, 1996	Hypotheses testing for construct validity	Follow-up *n*=204	For the standard six-item BSI Depression subscale, the rehospitalized group (*n*=51) had mean .78 (*SD* .92) versus .51 (*SD* .67) in the non-rehospitalized group (*n*=153), *t*=1.97, without a significance marker in the tabulated result. The prespecified significant-difference hypothesis was not supported.	-	D
Brief Symptom	Mitchell et al., 2008	Hypotheses testing for construct	40	Mitchell et al. (2008), *n*=40: BSI Depression correlated with DASS-21	+	D

PROM and version/form	Report or analysis	Measurement property or study component	Analysis sample, *n*	Reported result	COSMIN rating	RoB rating
Inventory – Depression subscale (English)		validity		Depression at *r*=0.70 (*p*<0.01). The same-construct hypothesis *r*≥0.50 was supported.		
Brief Symptom Inventory – Depression subscale (English)	Tate et al., 1990	Hypotheses testing for construct validity	SCI 79; normative 974; paired subset 20	BSI Depression raw score in SCI *n*=79 was mean .63 (*SD* .71) versus .28 (*SD* .46) in the nonpatient normative sample *n*=974; independent-samples *t*=4.27, *p*=.00001. The comparison was planned and remained significant with the Bonferroni criterion. The hypothesized higher depressive distress in SCI was supported, but the manual norm differed in age and sex and no adjustment was made. In the same SCI subsample *n*=20, BSI Depression standardized T score was 61.55 (*SD* 10.94; 6 items) and SCL-90-R Depression T score was 64.40 (*SD* 9.25; 13 items); paired *t*=2.47, *p*=.02. No correlation, agreement statistic or prespecified equivalence margin was reported, so the abbreviated-versus-full-form equivalence result was indeterminate. Additional SCL-90-R Depression items not contained in the BSI had the following SCI means/SDs: loss of sexual interest .6/1.2; low energy or slowed down 1.3/1.2; crying easily .6/.9; caught or trapped 1.1/1.3; blaming yourself .8/1.1; worrying too much 1.7/1.4; everything is an effort 1.9/1.1. The paired-form result was documented but not counted as an evaluable construct-validity hypothesis because no equivalence margin or agreement statistic was reported.	+	I
Brief Symptom Inventory – Depression subscale (English)	Tate et al., 1993	Hypotheses testing for construct validity	162	Outpatients *n*=162. BSI-D correlated with Zung total *r*=0.52, Zung somatic *r*=0.37, and Zung affective *r*=0.54 (all *p*<0.001). Dichotomized BSI-D/Zung agreement was 85%, kappa=0.59. All 3 prespecified/reconstructable BSI-D hypotheses were supported.	+	I
Beck Depression Inventory-I (English)	Radnitz et al., 1997	Internal consistency	124	Final analytic sample *n*=124. Cronbach's *alpha*=0.89 for the 21-item BDI-I. No eligible structural-validity evidence for the BDI-I total score was reported in this study; therefore internal consistency is indeterminate under COSMIN v2.	?	V
Beck Depression Inventory-I (English)	Radnitz et al., 1997	Criterion validity	124	Reference standard: independently administered SCID-*P* for DSM-III-R current Major Depressive Episode (either major depressive disorder or the depressive phase of bipolar disorder); 12/124 participants were positive. Research assistants received structured training; all 12 double-rated training interviews agreed on MDE diagnosis. Discriminant function analysis accounted for 59% of variance; 3/7 prespecified weak items were confirmed, and Somatic Preoccupation was also <0.30. Item–discriminant-function loadings: Indecisiveness 0.670; Sadness 0.559; Guilt 0.520; Insomnia 0.458; Suicidal Ideas 0.458; Social Withdrawal 0.450; Self-Dislike 0.423; Expectation of Punishment 0.416; Self-Accusations 0.414; Pessimism 0.409; Irritability 0.371; Loss of Appetite 0.358; Dissatisfaction 0.336; Crying 0.335; Fatigability 0.329; Loss of Libido 0.328; Sense of Failure 0.321; Somatic Preoccupation 0.294; Weight Loss 0.166; Body Image Change 0.163; Work Difficulty 0.140. SCID-*P* current MDE reference (12/124 positive). Cut-off 16: sensitivity 83.3%, specificity 85.3%, correctly classified 85.1%; 17: 83.3%, 90.0%, 89.3%; 18: 83.3%, 90.8%, 90.1%; 19: 83.3%, 90.8%, 90.1%; 20: 75.0%, 91.7%, 90.1%; 21: 75.0%, 93.6%, 91.7%; 22: 75.0%, 93.6%, 91.7%; 23: 66.7%, 96.3%, 93.4%; 24: 66.7%, 97.2%, 94.2%; 25: 58.3%, 98.1%, 94.2%; 26: 50.0%, 98.1%, 93.4%; 27: 50.0%, 100.0%, 95.0% in the tabulated result (95.2% in text); 28: 50.0%, 100.0%, 95.0%; 29: 41.7%, 100.0%, 94.2%; 30: 33.3%,	?	A

PROM and version/form	Report or analysis	Measurement property or study component	Analysis sample, *n*	Reported result	COSMIN rating	RoB rating
				100.0%, 93.4%. No ROC *AUC* or correlation with the diagnostic criterion was reported, so the criterion-validity result is indeterminate under the review's prespecified COSMIN rule. The authors noted that cut-offs were not externally cross-validated and should not be used alone to diagnose MDE.		
Ilfeld Psychiatric Symptom Index - Depression subscale (English)	Campagnolo et al., 2002	Hypotheses testing for construct validity	39	Campagnolo et al. (2002), chronic SCI *n*=39. The Ilfeld PSI depression-subscale score correlated with the Zung total score at *r*=0.78 (*p*<0.01).	+	I
Ilfeld Psychiatric Symptom Index - Depression subscale (English)	Campagnolo et al., 2002	Hypotheses testing for construct validity	59	Known-groups descriptive data, *n*=59: Ilfeld depression-subscale means were 16.2 (*SD* 12.1) for tetraplegia, 23.9 (18.1) for paraplegia, and 13.3 (11.2) for controls; both SCI subgroup means exceeded the control mean by >10%. No separate depression-subscale ANOVA or ROC analysis was reported. The article's *AUC*=0.55 and conventional ≥10 cutoff applied to the 29-item total Ilfeld index and were not assigned to the depression subscale.	+	A
Patient Health Questionnaire-9 (English)	Graves & Bombardier, 2008 - graded-response-model and subgroup-functioning analyses	Structural validity	3652	A two-parameter graded-response model was fitted to all nine items and test-information functions were reported. Item/category parameters, item-fit statistics, monotonicity, local independence and overall IRT model fit were not reported in extractable form. Full COSMIN IRT structural validity could therefore not be determined.	?	D
Patient Health Questionnaire-9 (English)	Graves & Bombardier, 2008 - one-factor CFA	Structural validity	3652	One-factor CFA in *n*=3,652: *chi-square*=847, *df*=27, *p*<0.001; GFI=0.95; *RMSEA*=0.091 (90% *CI* 0.086-0.097); NFI=0.924. Standardized item loadings for PHQ-9 items 1-9 were 0.72, 0.79, 0.60, 0.63, 0.59, 0.73, 0.60, 0.48 and 0.53. The authors judged the model adequate using older thresholds, but none of the current COSMIN CFA sufficiency criteria was met (*CFI*/*TLI* >0.95, *RMSEA* <0.06, or *SRMR* <0.08); structural validity was insufficient.	-	V
Patient Health Questionnaire-9 (English)	Kalpakjian et al., 2009	Structural validity	RS1/RS2 *n*=584 each; sex-specific analyses *n*=292 each	Principal-axis EFA with Promax rotation examined one- and two-factor solutions in two random samples (RS1 and RS2; *n*=584 each), and separately in men and women (*n*=292 per sex in each random sample). One-factor loadings, ordered as combined RS1/combined RS2/men RS1/men RS2/women RS1/women RS2: PHQ1 0.768/0.725/0.782/0.706/0.751/0.742; PHQ2 0.760/0.772/0.810/0.787/0.713/0.756; PHQ3 0.599/0.661/0.567/0.728/0.634/0.585; PHQ4 0.648/0.696/0.670/0.687/0.625/0.714; PHQ5 0.658/0.635/0.674/0.612/0.643/0.662; PHQ6 0.709/0.751/0.743/0.789/0.671/0.708; PHQ7 0.585/0.621/0.558/0.622/0.615/0.624; PHQ8 0.433/0.497/0.443/0.499/0.427/0.499; PHQ9 0.504/0.523/0.583/0.557/0.412/0.491. Eigenvalues were 3.666/3.917/3.890/4.064/3.458/3.793; variance explained was 41%/44%/38%/45%/45%/42%. Two-factor loadings, ordered as factor 1/factor 2 for combined RS1, combined RS2, men RS1, men RS2, women RS1, and women RS2: PHQ1 0.453/0.424, 0.496/0.349, 0.472/0.427, 0.550/0.307, 0.479/0.335, 0.573/0.308; PHQ2 0.609/0.266, 0.620/0.285, 0.648/0.282, 0.607/0.262, 0.651/0.254, 0.894/-0.034; PHQ3 0.066/0.641, 0.249/0.533,	-	V

PROM and version/form	Report or analysis	Measurement property or study component	Analysis sample, *n*	Reported result	COSMIN rating	RoB rating
				0.068/0.623, -0.070/0.894, 0.434/0.410, 0.339/0.425; PHQ4 0.066/0.703, 0.035/0.839, 0.100/0.723, 0.341/0.518, 0.063/0.811, 0.318/0.438; PHQ5 0.194/0.569, 0.325/0.417, 0.130/0.690, 0.360/0.426, 0.310/0.405, 0.276/0.515; PHQ6 0.619/0.201, 0.607/0.274, 0.786/0.082, 0.556/0.260, 0.710/0.200, 0.492/0.295; PHQ7 0.492/0.180, 0.425/0.297, 0.451/0.186, 0.627/0.085, 0.419/0.297, 0.365/0.370; PHQ8 0.234/0.261, 0.254/0.329, 0.352/0.155, 0.506/0.060, 0.087/0.520, 0.033/0.523; PHQ9 0.603/-0.013, 0.585/0.034, 0.678/-0.004, 0.532/0.022, 0.670/-0.030, 0.350/0.128. Eigenvalues for factors 1/2 were 2.917/2.883, 3.017/2.923, 3.208/2.927, 3.172/2.628, 3.332/2.934, and 2.831/2.265. Two-factor subscale alphas were 0.807/0.740, 0.817/0.743, 0.831/0.773, 0.811/0.762, 0.821/0.723, and 0.778/0.704. The two-factor solution explained 46%/48% in the combined samples, 50%/51% in men, and 43%/49% in women. Items PHQ1 and PHQ8 cross-loaded, and the two-factor pattern was unstable, especially among women. Under the COSMIN EFA criterion, the intended one-factor total score did not explain at least 50% of variance.		
Patient Health Questionnaire-9 (English)	Krause et al., 2008	Structural validity	568	Categorical maximum-likelihood CFA with the Mplus categorical command compared four models (*n*=568). One-factor 9-item model: *RMSEA*=0.110; standardized loadings—anhedonia 0.61, depressed mood 0.74, sleep 0.60, poor energy 0.71, appetite change 0.64, feelings of failure 0.77, concentration 0.75, psychomotor change 0.67, suicidal ideation 0.61. Preferred two-factor model with three somatic items: *RMSEA*=0.073; somatic loadings sleep 0.66, energy 0.79, appetite 0.71; general-factor loadings anhedonia 0.64, depressed mood 0.77, failure 0.79, concentration 0.77, psychomotor 0.68, suicidal ideation 0.63; factor correlation 0.76. Two-factor model with psychomotor change as a fourth somatic item: *RMSEA*=0.091; somatic loadings 0.63, 0.76, 0.71 and 0.68; general loadings 0.64, 0.77, 0.79, 0.78 and 0.63. The Results text gives the factor correlation as 0.92, whereas Tables 3 and 5 give 0.82; both values were retained. Eight-item two-factor model excluding psychomotor change: *RMSEA*=0.074; somatic loadings 0.67, 0.79 and 0.71; general loadings 0.64, 0.79, 0.80, 0.75 and 0.62; factor correlation 0.75. None of the models met the COSMIN sufficient-CFA threshold of *RMSEA*<0.06, and the original PHQ-9 total score was not supported as unidimensional.	-	V
Patient Health Questionnaire-9 (English)	Krause et al., 2010 - first follow-up EFA	Structural validity	211	At the first follow-up, approximately 17 months after SCI, two-factor EFA used *n*=211 and the published table reported *RMSEA*=0.09. Loadings factor 1/factor 2: anhedonia 0.70/0.14; depressed mood 0.85/0.02; sleep 0.37/0.42; fatigue 0.36/0.54; appetite 0.30/0.47; feeling bad about self 0.65/0.17; concentration 0.22/0.50; psychomotor -0.21/0.85; self-harm 0.65/-0.13. Factor 1 comprised anhedonia, depressed mood, feeling bad about self and self-harm; factor 2 comprised sleep, fatigue, appetite, concentration and psychomotor symptoms. The original total score was not supported as unidimensional. The first-follow-up results had been reported previously but the earlier source is not separately represented in this master file.	-	D
Patient Health Questionnaire-9	Krause et al., 2010 - second follow-up	Structural validity	302	At the second follow-up, approximately 29 months after SCI, the previously proposed six-nonsomatic/three-somatic CFA model showed poor fit	-	D

PROM and version/form	Report or analysis	Measurement property or study component	Analysis sample, *n*	Reported result	COSMIN rating	RoB rating
(English)	CFA/EFA			(*RMSEA*=0.110; analytic *n*=302). A subsequent two-factor EFA showed *RMSEA*=0.074 in the Results text and 0.08 after rounding in the tabulated result. Loadings factor 1/factor 2: anhedonia 0.55/0.29; depressed mood 0.27/0.63; sleep 0.90/-0.16; fatigue 0.75/0.01; appetite 0.65/0.10; feeling bad about self 0.10/0.78; concentration 0.42/0.27; psychomotor 0.34/0.26; self-harm -0.15/0.88. Factor 1 included anhedonia, sleep, fatigue, appetite and concentration; factor 2 included depressed mood, feeling bad about self and self-harm; psychomotor disturbance did not clearly load on either factor. The intended PHQ-9 total score was not supported as unidimensional. For context, baseline EFA *n*=554 had *RMSEA*=0.07 and loadings 0.37/0.23, 0.66/0.06, -0.03/0.63, -0.10/0.87, 0.14/0.49, 0.84/-0.13, 0.50/0.17, 0.36/0.23, and 0.53/-0.09; this baseline sample overlaps Krause et al. (2008) and was not counted again as independent evidence. Supplementary longitudinal predictive results not rated as a separate COSMIN property: baseline nonsomatic factor predicted second-follow-up nonsomatic *beta*=0.40 (*p*<0.001) and somatic *beta*=0.29 (*p*=0.006), whereas baseline somatic factor predicted neither nonsomatic *beta*=-0.01 nor somatic *beta*=0.07. First-follow-up nonsomatic predicted second-follow-up nonsomatic *beta*=0.66 but not somatic *beta*=0.09; first-follow-up somatic predicted second-follow-up somatic *beta*=0.66 but not nonsomatic *beta*=0.10. Item-level coefficients were highest for self-harm: 0.51, 0.44, and 0.70 across the three time-lag comparisons.		
Patient Health Questionnaire-9 (English)	Krause et al., 2011 - full-sample CFA	Structural validity	7296	Seven prespecified CFA models were tested using the categorical command and maximum-likelihood estimation in *n*=7,296. Model definitions: A, one factor/9 items; B, two factors/9 items with somatic sleep-appetite-fatigue and six nonsomatic items; C, two factors/9 items adding psychomotor change to the somatic factor; D, two factors/9 items with five somatic items (fatigue, sleep, appetite, concentration, psychomotor) and four nonsomatic items; E, two factors/8 items with somatic sleep-appetite-fatigue and five nonsomatic items, omitting psychomotor; *F*, two factors/8 items with somatic fatigue-sleep-appetite-anhedonia-concentration and nonsomatic suicidal ideation-feeling bad-depressed mood, omitting psychomotor; G, two factors/6 items with somatic sleep-appetite-fatigue and nonsomatic depressed mood-feeling bad-suicidal ideation. Full-sample *RMSEA* values A-G were 0.086/0.064/0.067/0.054/0.063/0.076/0.043. Factor correlations for Models B-G were 0.66/0.68/0.68/0.66/0.68/0.59. For the intended original 9-item total score, Model A had *CFI*=0.96; all models had *CFI*>0.90, but other exact *CFI* values were not reported. Although Model A *RMSEA* did not meet the COSMIN threshold, *CFI* exceeded 0.95; under the COSMIN OR rule, the one-factor result was rated sufficient. Alternative multidimensional models fit better: Model D was the best 9-item model (*RMSEA*=0.054), and Model G was the best overall model (*RMSEA*=0.043). The 6-item Model G and other alternative structures were analytical models only and were not treated as separately validated PROM versions. Subgroup *RMSEA* values A-G were: White *n*=5,273, 0.089/0.065/0.068/0.052/0.064/0.080/0.042; Black *n*=1,283, 0.077/0.065/0.066/0.052/0.064/0.063/0.039; Hispanic *n*=582, 0.094/0.074/0.081/0.079/0.078/0.095/0.077; men *n*=5,767,	+	V

PROM and version/form	Report or analysis	Measurement property or study component	Analysis sample, *n*	Reported result	COSMIN rating	RoB rating
				0.083/0.064/0.065/0.050/0.063/0.073/0.039; women *n*=1,529, 0.092/0.068/0.074/0.067/0.066/0.089/0.058; C1-C4 nonambulatory *n*=990, 0.082/0.061/0.070/0.053/0.061/0.070/0.034; C5-C8 nonambulatory *n*=1,691, 0.093/0.065/0.066/0.057/0.069/0.088/0.057; noncervical nonambulatory *n*=2,867, 0.079/0.061/0.063/0.049/0.061/0.067/0.035; ambulatory *n*=1,585, 0.086/0.064/0.066/0.055/0.061/0.081/0.045.		
Patient Health Questionnaire-9 (English)	Richardson & Richards, 2008	Structural validity	2570	Principal-axis factoring with Promax rotation was performed separately at 1, 5, 15 and 25 years postinjury (*n*=682, 517, 653 and 718). KMO indices were 0.88, 0.89, 0.90 and 0.87. Two correlated affective/somatic factors emerged at every time point; factor correlations were 0.73, 0.69, 0.75 and 0.71. Variance explained was 45.042%, 49.706%, 50.393% and 43.708%. Full loadings: 1 year - depressed 0.833/0.394, bad-self 0.757/0.335, death 0.668/0.145, interest 0.539/0.213, fatigue 0.007/0.711, sleep 0.027/0.625, psychomotor 0.086/0.562, concentration 0.054/0.539, appetite 0.043/0.538 (Affective/Somatic); 5 years - fatigue 0.892/0.125, sleep 0.770/0.075, appetite 0.564/0.095, interest 0.497/0.236, concentration 0.455/0.252, death 0.150/0.803, bad-self 0.037/0.754, depressed 0.285/0.602, psychomotor 0.252/0.245 (Somatic/Affective); 15 years - bad-self 0.896/0.086, death 0.636/0.076, depressed 0.628/0.232, concentration 0.603/0.073, interest 0.412/0.396, sleep 0.097/0.803, fatigue 0.023/0.763, appetite 0.201/0.570, psychomotor 0.369/0.098 (Affective/Somatic); 25 years - fatigue 0.858/0.100, sleep 0.660/0.020, appetite 0.645/0.052, psychomotor 0.402/0.022, death 0.231/0.731, depressed 0.175/0.721, bad-self 0.083/0.625, interest 0.376/0.382, concentration 0.347/0.275 (Somatic/Affective). The intended PHQ-9 total score was not supported as unidimensional, and item allocation was not fully stable across duration groups.	-	V
Patient Health Questionnaire-9 (English)	Williams et al., 2009 - CTT factor analysis	Structural validity	EFA *n*=91; CFA *n*=111	The total sample (*n*=202) was randomly split into EFA *n*=91 (inferred) and CFA *n*=111. EFA using polychoric correlations, unweighted least-squares estimation, and oblique quartimin rotation yielded two factors: affective 51.8% variance and somatic 14.9% variance; factor correlation=0.45. Rotated loadings (affective/somatic): anhedonia 0.449/-0.190; depressed mood 0.989/0.013; sleep disturbances 0.034/-0.702; low energy 0.467/-0.405; appetite disturbances 0.333/-0.430; worthlessness/guilt 0.860/0.073; trouble concentrating 0.286/-0.617; psychomotor agitation/retardation -0.114/-0.918; suicide/death thoughts 0.651/-0.085. Low energy cross-loaded. A one-factor categorical CFA using weighted least-squares estimation showed *chi-square*(12, *n*=111)=18.9, *p*=0.14, *CFI*=0.985, *TLI*=0.989, *RMSEA*=0.060. Because *CFI* and *TLI* exceeded 0.95, the one-factor model met the COSMIN CFA criterion for sufficient structural validity. Residual correlation between sleep disturbance and psychomotor agitation/retardation was *r*=-0.27, indicating local dependence.	+	V
Patient Health Questionnaire-9 (English)	Williams et al., 2009 - Rasch analysis	Structural validity	202	Rasch rating-scale analysis used *n*=202. Original four-category response use was 54% not at all, 23% several days, 7% more than half the days, and 15% nearly every day. Average measures were -15.2, -6.7, -1.0, and 13.3; step thresholds were -2.40, 5.78, and -3.38, showing disordered thresholds. Original	-	V

PROM and version/form	Report or analysis	Measurement property or study component	Analysis sample, *n*	Reported result	COSMIN rating	RoB rating
				four-category item statistics (average measure/infit mean square/infit Z/item-measure *r*): suicide/death thoughts 59.20/1.34/1.80/0.46; psychomotor agitation/retardation 56.40/1.48/2.70/0.47; trouble concentrating 52.70/0.99/0.00/0.59; appetite disturbances 51.00/1.12/1.00/0.57; worthlessness/guilt 50.20/0.86/-1.20/0.59; anhedonia 47.00/1.14/1.30/0.58; depressed mood 47.30/0.68/-3.30/0.70; low energy 44.20/0.96/-0.40/0.59; sleep disturbances 41.90/1.16/1.60/0.63. Psychomotor item misfit (infit=1.48); depressed mood was marginally below the authors' stated 0.70 infit lower bound. Person/item separation reliability was 0.62/0.96. After post-hoc collapsing of the two middle categories, item statistics were: suicide 63.30/1.22/1.40/0.51; psychomotor 60.90/1.36/2.40/0.50; concentration 54.10/0.95/-0.40/0.64; appetite 51.80/1.11/1.00/0.60; worthlessness/guilt 50.30/0.85/-1.40/0.66; anhedonia 44.90/1.09/0.90/0.61; depressed mood 45.60/0.69/-3.50/0.74; low energy 40.70/0.94/-0.60/0.67; sleep 38.40/1.18/1.80/0.61. Person/item separation reliability became 0.66/0.96. The post-hoc three-category rescoring was not treated as a separately validated PROM version. For the original four-category PHQ-9, disordered thresholds, local dependence (|*r*|=0.27), and item misfit meant that not all COSMIN Rasch structural-validity requirements were met.		
Patient Health Questionnaire-9 (English)	Williams et al., 2016 - original scoring	Structural validity	133 participants / 884 observations	Rasch rating-scale analysis used 884 repeated observations from 133 persons. Person-separation reliability=0.76; no misfitting items; point-measure correlations 0.44-0.72; variance explained 51.8% (eigenvalue 9.7); first residual contrast 9.8% (eigenvalue 1.8). Intermediate categories were underused and misordered: 'more than half the days' was never modal and 'several days' was infrequently endorsed. Disordered category functioning supports an insufficient result. Repeated observations were analysed without accounting for within-person dependence.	-	I
Patient Health Questionnaire-9 (English)	Bombardier et al., 2004	Internal consistency	849	Bombardier et al. (2004), *n*=849: Cronbach's *alpha*=0.87. Corrected item–total correlations ranged from 0.45 for psychomotor agitation/retardation and 0.48 for suicidal ideation to 0.69 for feelings of failure and 0.72 for depressed mood. No eligible structural-validity evidence for the PHQ-9 total score was reported in this study; internal consistency is therefore indeterminate under COSMIN v2.	?	V
Patient Health Questionnaire-9 (English)	Kalpakjian et al., 2009	Internal consistency	RS1/RS2 *n*=584 each; sex-specific analyses *n*=292 each	One-factor Cronbach's *alpha*, ordered as combined RS1/combined RS2/men RS1/men RS2/women RS1/women RS2, was 0.854/0.868/0.864/0.876/0.842/0.858. Two-factor subscale alphas were 0.807/0.740, 0.817/0.743, 0.831/0.773, 0.811/0.762, 0.821/0.723, and 0.778/0.704. Because the intended PHQ-9 total score did not have sufficient unidimensional structural validity, its internal consistency is indeterminate under COSMIN v2.	?	V
Patient Health Questionnaire-9 (English)	Krause et al., 2008	Internal consistency	568	Cronbach's *alpha* was 0.82 for the original 9-item total score. For the preferred two-factor model with three somatic items, *alpha* was 0.77 for the six-item non-somatic factor and 0.70 for the three-item somatic factor. For the four-somatic-item model, *alpha* was 0.74 for the non-somatic factor and 0.70 for the somatic factor. For the eight-item model excluding psychomotor change, *alpha*	?	V

PROM and version/form	Report or analysis	Measurement property or study component	Analysis sample, *n*	Reported result	COSMIN rating	RoB rating
				was 0.74 and 0.70. Because the original PHQ-9 total score did not have sufficient structural validity, its internal consistency is indeterminate under COSMIN v2.		
Patient Health Questionnaire-9 (English)	Krause et al., 2009	Internal consistency	NR (classification *n*=690)	Cronbach's *alpha*=0.89. The *alpha*-specific analysis *n* was not stated separately; the published table PHQ-9 classification counts totalled 690. This report did not perform a structural-validity analysis. Under COSMIN v2, internal consistency is indeterminate without at least low-certainty evidence for sufficient unidimensionality.	?	V
Patient Health Questionnaire-9 (English)	Poritz et al., 2018	Internal consistency	116	Cronbach's *alpha*=0.95 in *n*=116 community-residing Veterans with SCI/D. PHQ-9 was administered independently in written or oral format during routine care. No structural-validity analysis was performed in this report.	?	V
Patient Health Questionnaire-9 (English)	Richardson & Richards, 2008	Internal consistency	2570	PHQ-9 total-score Cronbach's *alpha* was 0.84, 0.87, 0.87 and 0.83 at 1, 5, 15 and 25 years. Factor-subscore alphas were: 1 year affective 0.81 and somatic 0.74; 5 years somatic 0.82 and affective 0.78; 15 years affective 0.84 and somatic 0.77; 25 years somatic 0.70 and affective 0.70. Because the intended total score was not unidimensional, total-score internal consistency is indeterminate under COSMIN v2.	?	V
Patient Health Questionnaire-9 (English)	Williams et al., 2009 - Rasch analysis	Internal consistency	202	For the original four-category PHQ-9, Rasch person-separation reliability was 0.62 and item-separation reliability was 0.96. After post-hoc collapsing of the two intermediate categories, the coefficients were 0.66 and 0.96. Cronbach's *alpha* or omega was not reported. COSMIN Version 2.0 has no formal result-rating threshold for Rasch separation reliability in the internal-consistency criterion, and the original Rasch analysis showed local dependence; the result was therefore rated indeterminate.	?	V
Patient Health Questionnaire-9 (English)	Williams et al., 2016 - original scoring	Internal consistency	133 participants / 884 observations	Cronbach's *alpha*=0.84 and person-separation reliability=0.76. Because original category functioning was insufficient and the repeated observations were not independent, internal consistency could not be rated sufficient.	?	I
Patient Health Questionnaire-9 (English)	Cook et al., 2017	Cross-cultural validity/measurement invariance	SCI 3,694; reference 3,000; total 6,694	Diagnosis-related DIF was evaluated by lordif ordinal logistic regression with a graded response model in SCI *n*=3,694 versus primary-care *n*=3,000. Combined-sample assumption checks were: *RMSEA*=0.087, *CFI*=0.975, *TLI*=0.966; EFA first-factor variance=63.7%, first/second eigenvalue ratio=7.6, factor correlation=0.807; no residual item pair exceeded 0.20. No PHQ-9 item met the study's standard meaningful-DIF criteria (>10% or >5% *beta* change, or McFadden delta-R2>0.05). At delta-R2>=0.005, items 1, 4, 5 and 9 were flagged, all for uniform DIF. DIF-corrected and uncorrected SCI scores correlated 0.994; difference range -3 to 3, mean -0.3871, *SD* 0.70528. Exact item-level McFadden delta-R2 values were not reported, so the COSMIN <0.02 criterion cannot be confirmed; the result was rated indeterminate.	?	I
Patient Health Questionnaire-9 (English)	Graves & Bombardier, 2008 - graded-response-model and subgroup-functioning analyses	Cross-cultural validity/measurement invariance	3652	For the full PHQ-9, reported relative-efficiency ratios were 0.97 for White versus non-White, 1.01 for time since injury <=2 versus >2 years, and 0.96 for education <=12 versus >12 years. Subgroup sizes and formal item-level DIF effect sizes were not reported. These information-function comparisons cannot establish COSMIN measurement invariance; the result was indeterminate.	?	I

PROM and version/form	Report or analysis	Measurement property or study component	Analysis sample, *n*	Reported result	COSMIN rating	RoB rating
Patient Health Questionnaire-9 (English)	Graves & Bombardier, 2008 - one-factor CFA	Cross-cultural validity/measurement invariance	Men 2,863; women 789	Sex-specific calibrations showed relative efficiency of the full PHQ-9 versus the total sample of 1.05 for men and 0.88 for women; women versus men=0.83. No individual item-parameter difference was statistically significant, but the authors described a cumulative sex effect. Because the analysis did not use a recognized multi-group CFA or formal item-level DIF model/effect-size criterion, measurement invariance was indeterminate.	?	I
Patient Health Questionnaire-9 (English)	Kalpakjian et al., 2009	Cross-cultural validity/measurement invariance	1,168 total; 292 women and 292 men in each random sample	Cross-sample one-factor congruence for combined/men/women was: Pearson loading correlations 0.948/0.791/0.925; Tucker coefficients 0.998/0.994/0.998; congruence tests 0.043/0.072/0.053. Cross-sample two-factor congruence, ordered combined factor1/factor2, men factor1/factor2, women factor1/factor2, was: Pearson 0.942/0.905, 0.768/0.715, 0.306/0.299; Tucker 0.983/0.970, 0.941/0.908, 0.858/0.773; congruence test 0.081/0.097, 0.165/0.184, 0.256/0.265. Gender congruence for the one-factor solution in RS1/RS2 was: Pearson 0.808/0.800, Tucker 0.994/0.996, congruence test 0.079/0.064. Gender congruence for the two-factor solution, ordered RS1 factor1/factor2 and RS2 factor1/factor2, was: Pearson 0.496/0.470 and 0.282/0.452; Tucker 0.872/0.843 and 0.858/0.809; congruence test 0.496/0.386 and 0.259/0.255. One-factor loading patterns were highly congruent across sex and split samples, but no multi-group CFA, formal loading/intercept equality test, or DIF analysis was performed; formal measurement invariance is therefore indeterminate.	?	I
Patient Health Questionnaire-9 (English)	Krause et al., 2010 - first follow-up EFA	Cross-cultural validity/measurement invariance	554, 211, and 302	The loading pattern changed markedly across inpatient baseline, approximately 17 months, and approximately 29 months after SCI. At baseline, sleep, fatigue and appetite formed the somatic factor; at first follow-up, concentration and psychomotor symptoms joined this factor; at second follow-up, anhedonia and concentration loaded with the somatic items, while psychomotor disturbance did not load clearly. Stability was assessed by separate CFA/EFA solutions, not by formal longitudinal measurement-invariance analysis. No equality constraints, multi-group/longitudinal CFA, threshold or intercept testing, or DIF analysis were conducted; therefore temporal measurement invariance was indeterminate.	?	I
Patient Health Questionnaire-9 (English)	Krause et al., 2011 - full-sample CFA	Cross-cultural validity/measurement invariance	582-5,767 across strata	The seven models were fitted separately by race/ethnicity, sex, and injury severity. Model G generally had the lowest *RMSEA*; the exception was the Hispanic subgroup, in which Model B was best (*RMSEA*=0.074), while no model showed excellent fit. One-factor Model A *RMSEA* values were 0.089 in White, 0.077 in Black, 0.094 in Hispanic, 0.083 in men, 0.092 in women, and 0.079-0.093 across injury-severity strata. However, the analyses were separate subgroup CFAs rather than formal multi-group measurement-invariance analyses. No configural/metric/scalar equality constraints or DIF testing were performed, the comparability of groups on other characteristics was not established, and the language profile of Hispanic participants was unknown. Formal measurement invariance therefore could not be determined.	?	I
Patient Health Questionnaire-9 (English)	Richardson & Richards, 2008	Cross-cultural validity/measurement invariance	2570	Separate exploratory factor analyses yielded broadly similar affective and somatic factors across 1-, 5-, 15- and 25-year groups. However, item allocation varied, some items failed to reach the 0.40 designation threshold, and no multi-group CFA, formal equality testing or DIF analysis was performed. Groups	?	I

PROM and version/form	Report or analysis	Measurement property or study component	Analysis sample, *n*	Reported result	COSMIN rating	RoB rating
				also differed significantly in age, race and neurological impairment. The measurement-invariance result is therefore indeterminate.		
Patient Health Questionnaire-9 (English)	Bombardier et al., 2012	Criterion validity	142	PHQ-9 was interview-administered by trained research assistants. The SCID DSM-IV MDD module was administered to all participants regardless of PHQ-9 score by trained mental-health professionals with SCI experience who were blinded to PHQ-9 results. Mean interval between PHQ-9 and SCID was 3.1 days (*SD* 2.2), and the final analysis required an interval <=7 days. Complete the published table PHQ-9 diagnostic-accuracy results against SCID MDD (*N*=142; SCID MDD *n*=14): total score >=9: 46 (32%), sensitivity 1.00 (*95% CI* 0.78-1.00), specificity 0.75 (0.67-0.82), Youden 0.75, *PPV* 0.30 (0.18-0.46), *NPV* 1.00 (0.96-1.00), *LR*+ 4.00 (2.96-5.40), *LR*- 0, kappa 0.37 (0.22-0.52); >=10: 39 (27%), sensitivity 1.00 (0.78-1.00), specificity 0.80 (0.73-0.86), Youden 0.80, *PPV* 0.36 (0.21-0.53), *NPV* 1.00 (0.96-1.00), *LR*+ 5.12 (3.60-7.28), *LR*- 0, kappa 0.45 (0.28-0.61); >=11: 35 (25%), sensitivity 1.00 (0.78-1.00), specificity 0.84 (0.76-0.89), Youden 0.84, *PPV* 0.40 (0.24-0.58), *NPV* 1.00 (0.97-1.00), *LR*+ 6.10 (4.12-9.01), *LR*- 0, kappa 0.50 (0.33-0.67); >=12: 29 (20%), sensitivity 0.86 (0.60-0.96), specificity 0.87 (0.80-0.92), Youden 0.72, *PPV* 0.41 (0.24-0.61), *NPV* 0.98 (0.94-1.00), *LR*+ 6.45 (3.95-10.55), *LR*- 0.17 (0.05-0.60), kappa 0.49 (0.30-0.68); total >=9 plus at least one cardinal item >=2: 28 (20%), sensitivity 0.64 (0.39-0.84), specificity 0.85 (0.78-0.90), Youden 0.49, *PPV* 0.32 (0.16-0.52), *NPV* 0.96 (0.90-0.99), *LR*+ 4.33 (2.45-7.66), *LR*- 0.42 (0.21-0.85), kappa 0.34 (0.14-0.54); total >=10 plus cardinal >=2: 26 (18%), sensitivity 0.64 (0.39-0.84), specificity 0.87 (0.80-0.92), Youden 0.51, *PPV* 0.35 (0.17-0.56), *NPV* 0.96 (0.90-0.99), *LR*+ 4.84 (2.68-8.73), *LR*- 0.41 (0.20-0.83), kappa 0.37 (0.16-0.57); total >=11 plus cardinal >=2: identical to the >=10/cardinal rule; total >=12 plus cardinal >=2: 21 (15%), sensitivity 0.50 (0.27-0.73), specificity 0.89 (0.82-0.93), Youden 0.39, *PPV* 0.33 (0.15-0.57), *NPV* 0.94 (0.88-0.98), *LR*+ 4.57 (2.22-9.39), *LR*- 0.56 (0.33-0.95), kappa 0.32 (0.10-0.54); DSM-IV algorithm, at least five symptoms >=2 and at least one cardinal >=2: 15 (11%), sensitivity 0.50 (0.27-0.73), specificity 0.94 (0.88-0.97), Youden 0.44, *PPV* 0.47 (0.21-0.73), *NPV* 0.94 (0.89-0.98), *LR*+ 8.00 (3.42-18.74), *LR*- 0.53 (0.32-0.90), kappa 0.42 (0.18-0.67); lowered-frequency algorithm, at least five symptoms >=1 and one cardinal >=1: 58 (41%), sensitivity 1.00 (0.78-1.00), specificity 0.66 (0.57-0.73), Youden 0.66, *PPV* 0.24 (0.14-0.37), *NPV* 1.00 (0.96-1.00), *LR*+ 2.91 (2.29-3.70), *LR*- 0, kappa 0.27 (0.15-0.40). Overall ROC *AUC*=0.921 (reported as 0.92 in text). The abstract gives the cutoff-11 *NPV CI* as 0.96-1.00, whereas the published table gives 0.97-1.00. The article contains participant-flow inconsistencies: the abstract reports 204 eligible patients, whereas Results reports 205 enrolled; Methods states *n*=37 were excluded because PHQ-9 and SCID could not be completed within 7 days, but 173 received SCID and 142 entered the final analysis, implying 31 with SCID were outside the paired-analysis window. The final 142 were broadly comparable with excluded/nonpaired participants on sex, tetraplegia, age, days since injury and PHQ-9 score, with a borderline racial-distribution difference (*p*=0.051). The optimal cutoff >=11 and the conventional cutoff >=10 both met the study's	+	A

PROM and version/form	Report or analysis	Measurement property or study component	Analysis sample, *n*	Reported result	COSMIN rating	RoB rating
				good-accuracy Youden threshold; *AUC* exceeded 0.70.		
Patient Health Questionnaire-9 (English)	Bombardier et al., 2004	Hypotheses testing for construct validity	144	Four-site subsample *n*=144: PHQ-9 severity correlated with Satisfaction With Life Scale at Spearman r_s=-0.51 and with poorer subjective health at r_s=0.50 (both *p*<0.001). Both prespecified hypotheses were supported.	+	D
Patient Health Questionnaire-9 (English)	Bombardier et al., 2004	Hypotheses testing for construct validity	638	Participants reporting at least one depressive symptom, *n*=638: PHQ-9 severity correlated with greater difficulty in daily role functioning at Spearman r_s=0.62 (*p*<0.001). The prespecified hypothesis was supported.	+	D
Patient Health Questionnaire-9 (English)	Bombardier et al., 2004	Hypotheses testing for construct validity	849	Known-groups hypotheses, *n*=849, used the PHQ-9 DSM-IV-based probable-MDD classification. Rates did not differ by injury level, injury completeness, or discharge motor FIM (3 hypotheses supported). The predicted higher rate among women was not observed, and the youngest group did not have the highest rate; rates were 6.5% in the youngest group, 15.0% at ages 25–49, and 8.7% in the older group (2 hypotheses not supported). Thus 3/5 known-groups hypotheses (60%) were supported. Item-level sensitivity, specificity, predictive values, and likelihood ratios against PHQ-9-defined probable MDD were not treated as criterion validity because the reference classification was derived from the same nine PHQ-9 items.	-	A
Patient Health Questionnaire-9 (English)	Bombardier et al., 2012	Hypotheses testing for construct validity	Maximum 142; role-difficulty *n* NR	PHQ-9 total score correlations, all *p*<0.001: poorer subjective health on SF-1 r_s=0.37 (higher SF-1 scores indicate poorer health); EuroQOL current-health-state thermometer r_s=-0.38 (higher scores indicate better health); Life-1 overall quality of life since SCI r_s=-0.38 (higher scores indicate better quality of life); difficulty with inpatient rehabilitation therapies or getting along with others r_s=0.37. The role-difficulty analysis was restricted to participants with PHQ-9 scores >0; its analytic *n* was not reported. All four prespecified directional magnitude hypotheses were supported.	+	D
Patient Health Questionnaire-9 (English)	Krause et al., 2009	Hypotheses testing for construct validity	Reported as *r*(655)	PHQ-9 and OAHMQ continuous scores were strongly correlated: Spearman *r*=0.781, reported as *r*(655), *p*<0.0001; the same-construct hypothesis *r*>=0.50 was met. Supplementary correlations from the tabulated result: PHQ-9 with OAHMQ>=6 *r*=0.678 and OAHMQ>=11 *r*=0.610; PHQ-9 algorithm MDD with OAHMQ continuous *r*=0.477, OAHMQ>=6 *r*=0.389, OAHMQ>=11 *r*=0.605; PHQ-9>=15 with OAHMQ *r*=0.440, OAHMQ>=6 *r*=0.356, OAHMQ>=11 *r*=0.527; PHQ-9>=10 with OAHMQ *r*=0.621, OAHMQ>=6 *r*=0.556, and OAHMQ>=11 *r*=0.693. All *p*<0.0001. These classification correlations were retained as supplementary construct/interpretability information, not criterion validity.	+	D
Patient Health Questionnaire-9 (English)	Krause et al., 2009	Hypotheses testing for construct validity	Reported as *r*(678)	PHQ-9 correlated negatively with SWLS: continuous score *r*=-0.477, reported as *r*(678), *p*<0.0001; PHQ-9 algorithm MDD *r*=-0.335; PHQ-9>=15 *r*=-0.342; PHQ-9>=10 *r*=-0.408. The related-construct hypothesis |*r*|>=0.30 was met. Mean SWLS was lower for PHQ-9 algorithm MDD versus no MDD [11.87 (*SD* 6.49) vs 21.67 (7.97)], PHQ-9>=15 versus <15 [10.94 (5.96) vs 21.62 (7.96)], and PHQ-9>=10 versus <10 [13.22 (7.15) vs 22.42 (7.65)]; all *p*<0.0001. No independent psychiatric diagnostic interview was performed.	+	D
Patient Health Questionnaire-9	Richardson & Richards, 2008	Hypotheses testing for construct validity	670, 509, 640, and 709	Factor-derived PHQ-9 subscores correlated negatively with SWLS. Affective/somatic correlations were -0.463/-0.346 at 1 year (*n*=670), -0.456/-	+	D

PROM and version/form	Report or analysis	Measurement property or study component	Analysis sample, *n*	Reported result	COSMIN rating	RoB rating
(English)				0.415 at 5 years (*n*=509), -0.404/-0.248 at 15 years (*n*=640), and -0.368/-0.255 at 25 years (*n*=709), all *p*<0.001. Six of eight correlations met the prespecified |*r*|≥0.30 threshold; all eight were in the expected negative direction.		
Patient Health Questionnaire-9 (English)	Tulsky et al., 2015 - SCI-QOL Depression comparison	Hypotheses testing for construct validity	465	PHQ-9 correlated *r*=0.76 with SCI-QOL Depression in *n*=465. The prespecified same-construct hypothesis *r*>=0.50 was met. PHQ items were recoded 1-4 only for linkage, a linear shift from conventional 0-3 scoring. The crosswalk is appropriate for group-level use but not exact individual interchangeability. PHQ-9 1–4-coded raw score→SCI-QOL T-score (*SE*): 9→22 (9.85); 10→46 (3.50); 11→48 (2.50); 12→49 (2.00); 13→50 (1.70); 14→51 (1.48); 15→52 (1.32); 16→53 (1.19); 17→53 (1.09); 18→54 (1.00); 19→55 (0.92); 20→55 (0.86); 21→56 (0.80); 22→56 (0.75); 23→57 (0.71); 24→58 (0.67); 25→58 (0.63); 26→59 (0.59); 27→60 (0.56); 28→60 (0.54); 29→61 (0.51); 30→62 (0.49); 31→63 (0.47); 32→63 (0.45); 33→64 (0.43); 34→65 (0.41); 35→66 (0.40); 36→67 (0.38)	+	A
Hospital Anxiety and Depression Scale - Depression subscale (English)	Woolrich et al., 2006	Structural validity	963	Woolrich et al. (2006), *n*=963. PCA with direct-oblimin rotation extracted two factors (eigenvalues 5.6 and 1.6), explaining 51.2% of total variance. For the target HADS-D subscale, all seven depression items loaded on the depression factor: item 2=0.722, item 4=0.741, item 6=0.729, item 8=0.544, item 10=0.666, item 12=0.797, item 14=0.394. Anxiety item 7 loaded on the depression factor (0.544), and anxiety item 1 cross-loaded at 0.309; these findings complicate the overall two-subscale HADS structure but do not negate the loading pattern of the independently scored HADS-D items.	+	D
Hospital Anxiety and Depression Scale - Depression subscale (English)	Woolrich et al., 2006	Internal consistency	963	Cronbach's *alpha*=0.79 for HADS-D (*n*=963). *Alpha* remained 0.79 if item 14 was removed and decreased if any other depression item was removed.	+	V
Hospital Anxiety and Depression Scale - Depression subscale (English)	Woolrich et al., 2006	Cross-cultural validity/measurement invariance	963	Separate PCAs by sex and injury level generally produced two factors. Total explained variance was 51.0% in men, 52.2% in women, 49.0% in tetraplegia, and 52.6% in paraplegia. Item 14 was unstable among women and participants with tetraplegia; item 8 cross-loaded in tetraplegia. No formal multi-group CFA, DIF analysis, or statistical test of measurement invariance was performed, so the result is indeterminate.	?	I
Hospital Anxiety and Depression Scale - Depression subscale (English)	Woolrich et al., 2006	Hypotheses testing for construct validity	NR (maximum 963)	HADS-D correlated negatively with the Life Satisfaction Questionnaire at *r*=-0.660 (*p*<0.001). The analysis-specific sample size was not reported (maximum possible *n*=963). The prespecified direction and magnitude hypothesis was supported.	+	D
CES-D-10 (English)	Miller et al., 2008	Internal consistency	47	Miller et al. (2008), *n*=47: Cronbach's *alpha*=0.86. Deleting any individual item changed *alpha* by no more than 0.05. No structural-validity analysis of the 10-item short form was conducted.	?	V
CES-D-10 (English)	Miller et al., 2008	Reliability	47	Two-week test–retest reliability, *n*=47: *ICC*(1,1)=0.85 (*95% CI* 0.75–0.92). CES-D-10 item ICCs (items identified in bold in the tabulated result): item 1 0.32 (0.04–0.55); 5 0.62 (0.41–0.77); 6 0.67 (0.48–0.80); 7 0.59 (0.37–0.75); 8 0.59 (0.37–0.75); 10 0.47 (0.22–0.67); 11 0.64 (0.43–0.78); 12 0.52 (0.28–0.70); 14 0.55 (0.32–0.72); 20 0.68 (0.49–0.81).	+	A

PROM and version/form	Report or analysis	Measurement property or study component	Analysis sample, *n*	Reported result	COSMIN rating	RoB rating
CES-D-10 (English)	Miller et al., 2008	Measurement error	47	Bland–Altman analysis indicated minimal systematic bias. Session 1 minus session 2 mean difference=-0.22 (*95% CI* -1.21 to 0.78; *t*=-0.44, *p*=0.66). The article reports that three of 47 points lay outside ±2 *SD* but does not clearly assign that count to a specific CES-D version. The authors stated that <15% scored at either scale extreme. Numeric LoA and an MIC were not reported.	?	A
CES-D-10 (English)	Miller et al., 2008	Hypotheses testing for construct validity	47	A priori convergent-validity result, *n*=47: CES-D-10 correlated positively with VAS-*F* at *r*=0.57. The hypothesis *r*>0.50 was supported.	+	I
CES-D-10 (English)	Miller et al., 2008	Hypotheses testing for construct validity	47	A priori SF-36 correlations, *n*=47: mental health 0.71, emotional role 0.56, vitality 0.60, pain 0.38, social role 0.42, physical function 0.37, physical role 0.49, and general health 0.60. The published coefficients are printed positive signs, while the Methods, Results, and Discussion explicitly state that the expected negative direction was observed. Because the exact signed coefficients are internally inconsistent, this result set was rated indeterminate.	?	I
Depression Anxiety Stress Scales-21 - Depression subscale (English)	Mitchell et al., 2008	Criterion validity	40	Reference standard: MINI English Version 5.0.0 structured diagnostic interview, administered by an onsite clinical psychologist blinded to questionnaire results. Seven of 40 participants had a depression diagnosis (4 major depressive episodes and 3 dysthymia). DASS-21 Depression correctly identified 3/4 major depressive episodes and 1/3 dysthymia cases; sensitivity was 57% and specificity 76%. *AUC* and correlation with the diagnostic criterion were not reported, so the result is indeterminate under the review's COSMIN rule.	?	A
Depression Anxiety Stress Scales-21 - Depression subscale (English)	Mitchell et al., 2008	Hypotheses testing for construct validity	40	Mitchell et al. (2008), *n*=40: DASS-21 Depression correlated with BSI Depression at *r*=0.70 (*p*<0.01). It also correlated significantly with seven other BSI subscales and the GSI, but exact coefficients were not reported and were not rated as additional hypotheses.	+	I
Older Adult Health and Mood Questionnaire (English)	Krause et al., 2009	Internal consistency	NR (classification *n*=682)	Cronbach's *alpha*=0.87. The *alpha*-specific analysis *n* was not stated separately; the published table OAHMQ classification counts totalled 682. This report did not evaluate structural validity. Under COSMIN v2, internal consistency is indeterminate without sufficient unidimensionality evidence.	?	V
Older Adult Health and Mood Questionnaire (English)	Krause et al., 2009	Hypotheses testing for construct validity	Reported as *r*(655)	OAHMQ and PHQ-9 continuous scores were strongly correlated: Spearman *r*=0.781, reported as *r*(655), *p*<0.0001; the same-construct hypothesis *r*>=0.50 was met. OAHMQ correlations with PHQ-9 algorithm MDD, PHQ-9>=15, and PHQ-9>=10 were 0.477, 0.440, and 0.621; OAHMQ>=6 correlations with these classifications were 0.389, 0.356, and 0.556; OAHMQ>=11 correlations were 0.605, 0.527, and 0.693. Same-sample classification agreement: against PHQ-9 algorithm MDD, OAHMQ>=6 sensitivity/specificity 100.00%/63.20%, kappa=0.26, and OAHMQ>=11 89.71%/88.76%, kappa=0.56; against PHQ-9>=15, OAHMQ>=6 100.00%/62.14%, kappa=0.23, and OAHMQ>=11 86.21%/87.10%, kappa=0.48; against PHQ-9>=10, OAHMQ>=6 99.22%/70.21%, kappa=0.48, and OAHMQ>=11 75.00%/94.12%, kappa=0.69. Exploratory optimal OAHMQ cutoffs were 12 for PHQ-9 algorithm MDD (sensitivity 86.8%, specificity 92.7%, *AUC* 0.95017), 10 for PHQ-9>=15 (91.4%, 85.1%, *AUC* 0.94562), and 9 for PHQ-9>=10 (85.9%, 89.6%, *AUC* 0.95091). The PHQ-9 was not an independent diagnostic gold standard and has	+	I

PROM and version/form	Report or analysis	Measurement property or study component	Analysis sample, *n*	Reported result	COSMIN rating	RoB rating
				mixed/insufficient structural-validity evidence in SCI; these results were not treated as criterion validity.		
Older Adult Health and Mood Questionnaire (English)	Krause et al., 2009	Hypotheses testing for construct validity	Reported as *r*(671)	OAHMQ correlated negatively with SWLS: continuous score *r*=-0.538, reported as *r*(671), *p*<0.0001; OAHMQ>=6 *r*=-0.485; OAHMQ>=11 *r*=-0.443. The related-construct hypothesis |*r*|>=0.30 was met. Mean SWLS was lower for OAHMQ>=11 versus <11 [12.46 (*SD* 6.70) vs 22.53 (7.61)], *p*<0.0001.	+	D
Hospital Anxiety and Depression Scale - Depression subscale (German)	Müller et al., 2012	Structural validity	100	Partial-credit Rasch analysis used *n*=100. Overall HADS-D *chi-square*=24.411 (*df*=14, *p*=0.041). Item statistics (location/*SE*/*z*/*chi-square*/*df*/*p*), all with ordered four-category thresholds: Dep2 'I still enjoy the things I used to enjoy' 0.156/0.180/-1.259/3.926/2/0.140; Dep4 'I can laugh and see the funny side of things' 0.747/0.185/-1.136/2.544/2/0.281; Dep6 'I feel cheerful' 0.560/0.178/-0.763/5.233/2/0.073; Dep8 'I feel as if I am slowed down' -1.907/0.172/0.066/2.148/2/0.342; Dep10 'I have lost interest in my appearance' 0.586/0.174/0.735/4.678/2/0.096; Dep12 'I look forward with enjoyment to things' -1.249/0.162/-0.464/0.003/2/0.998; Dep14 'I can enjoy a good book or radio or TV program' 1.107/0.190/1.056/5.878/2/0.053. All item *z* residuals were within -2 to 2 and no item misfit was reported. Residual PCA eigenvalue=1.63 and exactly 5.00% of independent *t* tests were significant. Mean person location=-1.49 (*SD* 1.86); 4% were below the lowest threshold and no ceiling effect was reported. Under COSMIN Version 2.0, sufficient Rasch structural validity additionally requires explicit evidence for unidimensionality using *CFI*/*TLI*/*RMSEA*/*SRMR*, local independence using residual correlations or Q3, and monotonicity using item-response graphs or scalability. These were not directly reported; the result is indeterminate.	?	D
Hospital Anxiety and Depression Scale - Depression subscale (German)	Müller et al., 2012	Internal consistency	100	Rasch person reliability index=0.82 for the seven-item HADS-D. COSMIN recognizes an IRT-based separation reliability coefficient as an appropriate study method, but the Version 2.0 criteria for rating internal consistency require at least low evidence for sufficient unidimensionality and a Cronbach *alpha* >=0.70. Cronbach *alpha* or omega was not reported in this study, and sufficient Rasch structural validity was not established; the result is indeterminate.	?	V
Hospital Anxiety and Depression Scale - Depression subscale (German)	Müller et al., 2012	Cross-cultural validity/measurement invariance	100	No statistically significant uniform or non-uniform DIF was detected for age (< versus >=52 years), gender, relationship status, lesion level (paraplegia versus tetraplegia), or education (< versus >=13 years) using Rasch residual ANOVA with Bonferroni-adjusted *alpha*=0.00143. However, the total analysed sample was only *n*=100, every comparison group was below 100, group similarity on other relevant characteristics was not demonstrated, and DIF effect-size estimates such as McFadden R2 change were not reported. The COSMIN criterion of no important DIF (McFadden R2<0.02) therefore cannot be confirmed.	?	I
Hospital Anxiety and Depression Scale - Depression subscale (Turkish)	Paker et al., 2013	Internal consistency	175	Cronbach's *alpha* for the Turkish HADS-D was 0.77 in *n*=175 adults with traumatic SCI. The calculation and sample size were adequate. However, this study did not evaluate structural validity of the Turkish HADS-D in SCI. Structural-validity evidence from English or German HADS-D versions was	?	V

PROM and version/form	Report or analysis	Measurement property or study component	Analysis sample, *n*	Reported result	COSMIN rating	RoB rating
				not transferred across language versions. Therefore, despite *alpha* >=0.70, the internal-consistency result was rated indeterminate under COSMIN. The article's use of the term 'reliability' refers only to internal consistency; no test-retest reliability parameter was reported.		
SCI-QOL Depression Item Bank (English)	Tulsky et al., 2015	PROM development - concept elicitation	NR	Patient and clinician focus groups defined the depression domain and generated or retained items across the severity continuum. Expert item review was used to identify content gaps and add or revise items. The process supported item relevance and comprehensiveness, but participant numbers and several methodological details required for a complete COSMIN development appraisal were not reported in this article.	+ for relevance; + for comprehensiveness	D
SCI-QOL Depression Item Bank (English)	Tulsky et al., 2015	PROM development - cognitive interviews/pilot testing	NR	The preliminary items underwent cognitive debriefing, and no item required modification on the basis of participant feedback. All items were written at or below a fifth-grade reading level. Reporting of the instructions, response options, sample characteristics, and cognitive-debriefing findings was insufficient to confirm comprehensibility under the COSMIN criteria.	?	D
SCI-QOL Depression Item Bank (English)	Tulsky et al., 2015	Content validity - overall PROM-development evidence	NR	The SCI-specific mixed-method development process supported relevance and comprehensiveness. Comprehensibility remained indeterminate because the cognitive-debriefing methods and results were incompletely reported; the overall content-validity result was therefore indeterminate.	?	D
SCI-QOL Depression Item Bank (English)	Tulsky et al., 2015 - CFA, internal consistency, DIF, and PHQ-9 comparison	Structural validity	716	Final 28-item CFA in *n*=716: *CFI*=0.968 and *RMSEA*=0.066. *CFI* exceeded the COSMIN threshold of 0.95, so the CTT unidimensional result was sufficient under the OR rule. Twenty-six items had R2>0.40, two had R2<0.40, no residual correlation exceeded |0.20|, and the first-to-second eigenvalue ratio was 14.3. Complete the published table item statistics: 1. Dep_31 | I had given up on my dreams. | mean=1.76, *SD*=1.098, %min=59.9, %max=2.7 2. EDDEP04*P* | I felt worthless. | mean=1.66, *SD*=1.025, %min=63.6, %max=2.5 3. EDDEP05*P* | I felt that I had nothing to look forward to. | mean=1.66, *SD*=1.017, %min=63.1, %max=2.2 4. EDDEP06*P* | I felt helpless. | mean=1.99, *SD*=1.155, %min=48.0, %max=4.5 5. EDDEP07*P* | I withdrew from other people. | mean=1.72, *SD*=0.973, %min=56.6, %max=1.1 6. EDDEP09*P* | I felt that nothing could cheer me up. | mean=1.73, *SD*=0.988, %min=56.7, %max=1.7 7. EDDEP17*P* | I felt sad. | mean=2.38, *SD*=1.122, %min=27.0, %max=4.7 8. EDDEP19*P* | I felt that I wanted to give up on everything. | mean=1.42, *SD*=0.853, %min=75.8, %max=1.3 9. EDDEP28*P* | I felt lonely. | mean=2.24, *SD*=1.184, %min=37.2, %max=4.1 10. EDDEP29*P* | I felt depressed. | mean=2.04, *SD*=1.112, %min=43.3, %max=3.4 11. EDDEP31*P* | I felt discouraged about the future. | mean=2.16, *SD*=1.139, %min=39.1, %max=3.4 12. EDDEP35*P* | I found that things in my life were overwhelming. | mean=2.05, *SD*=1.085, %min=41.3, %max=3.4 13. EDDEP36*P* | I felt unhappy. | mean=2.22, *SD*=1.117, %min=33.8, %max=3.8 14. EDDEP39*P* | I felt I had no reason for living. | mean=1.35, *SD*=0.805, %min=79.3, %max=1.4 15. EDDEP41*P* | I felt hopeless. | mean=1.65, *SD*=0.987, %min=63.0, %max=2.0 16. EDDEP45*P* | I felt that nothing was interesting. | mean=1.78, *SD*=1.022, %min=54.6, %max=2.1 17. EDDEP46*P* | I felt pessimistic. | mean=2.01, *SD*=1.073, %min=43.4, %max=2.2 18.	+	A

PROM and version/form	Report or analysis	Measurement property or study component	Analysis sample, *n*	Reported result	COSMIN rating	RoB rating
				EDDEP48*P* | I felt that my life was empty. | mean=1.78, *SD*=1.055, %min=55.9, %max=2.5 19. EDDEP54*P* | I felt emotionally exhausted. | mean=2.09, *SD*=1.127, %min=42.0, %max=3.6 20. NQDEP01*N* | I felt lonely even when I was with other people. | mean=1.74, *SD*=1.000, %min=57.4, %max=1.8 21. NQDEP08*N* | I was critical of myself for my mistakes. | mean=2.47, *SD*=1.242, %min=30.3, %max=7.0 22. NQDEP15 | I wished I were dead and away from it all. | mean=1.34, *SD*=0.832, %min=81.8, %max=1.7 23. NQDEP16 | I thought about suicide. | mean=1.20, *SD*=0.625, %min=88.5, %max=0.8 24. NQDEP20*N* | I felt unloved. | mean=1.53, *SD*=0.931, %min=69.0, %max=2.1 25. NQDEP22 | I felt that others would be better off if I were dead. | mean=1.40, *SD*=0.890, %min=78.4, %max=2.4 26. NQDEP26*N* | I had trouble keeping my mind on what I was doing. | mean=2.09, *SD*=1.058, %min=38.4, %max=2.0 27. NQDEP29*N* | I felt like I needed help for my depression. | mean=1.66, *SD*=0.997, %min=62.4, %max=1.8 28. NQDEP30*N* | I had trouble enjoying things that I used to enjoy. | mean=2.40, *SD*=1.255, %min=34.0, %max=7.1		
SCI-QOL Depression Item Bank (English)	Tulsky et al., 2015 - graded-response IRT calibration	Structural validity	716	Graded-response IRT calibration in *n*=716. Original SCI-specific slopes ranged 1.43-4.36 and thresholds -0.87 to 2.84; no residual correlation exceeded |0.20|. The article stated that all final items had adequate or better S-X2 fit while reporting *P*<0.05, an internal contradiction. Two retained items showed inversion of average raw scores between response categories 4 and 5. Monotonicity was not directly evaluated with item-response graphs or scalability coefficients, and exact item-level S-X2 statistics were not reported; full COSMIN IRT structural validity was therefore indeterminate. Complete the published table PROMIS-linked IRT parameters: 1. Dep_31 | slope=2.15998; b1=0.41389, b2=1.02506, b3=1.69092, b4=2.54440 2. EDDEP04*P* | slope=3.55165; b1=0.47556, b2=0.93740, b3=1.70107, b4=2.18427 3. EDDEP05*P* | slope=4.23071; b1=0.44395, b2=0.93198, b3=1.65946, b4=2.14865 4. EDDEP06*P* | slope=2.32652; b1=0.05125, b2=0.66820, b3=1.62690, b4=2.18065 5. EDDEP07*P* | slope=2.28323; b1=0.30366, b2=1.01594, b3=1.97905, b4=2.98945 6. EDDEP09*P* | slope=3.27472; b1=0.28513, b2=0.93234, b3=1.79038, b4=2.35423 7. EDDEP17*P* | slope=2.82139; b1=-0.60395, b2=0.22965, b3=1.27734, b4=1.98449 8. EDDEP19*P* | slope=3.43829; b1=0.83163, b2=1.32431, b3=1.98523, b4=2.56590 9. EDDEP28*P* | slope=1.77956; b1=-0.33732, b2=0.38464, b3=1.45255, b4=2.49997 10. EDDEP29*P* | slope=3.40087; b1=-0.07614, b2=0.52751, b3=1.47203, b4=2.06163 11. EDDEP31*P* | slope=2.77590; b1=-0.19851, b2=0.42318, b3=1.39802, b4=2.18645 12. EDDEP35*P* | slope=2.15291; b1=-0.15561, b2=0.62625, b3=1.82065, b4=2.40451 13. EDDEP36*P* | slope=3.28354; b1=-0.35504, b2=0.38967, b3=1.34430, b4=2.02462 14. EDDEP39*P* | slope=3.39440; b1=0.96514, b2=1.48114, b3=2.01474, b4=2.49881 15. EDDEP41*P* | slope=4.22822; b1=0.44127, b2=0.96638, b3=1.71221, b4=2.21806 16. EDDEP45*P* | slope=2.52519; b1=0.24102, b2=0.89763, b3=1.84310, b4=2.48422 17. EDDEP46*P* | slope=2.11924; b1=-0.10251, b2=0.64033, b3=1.79079, b4=2.67257 18. EDDEP48*P* | slope=3.02589; b1=0.26893, b2=0.91047,	?	D

PROM and version/form	Report or analysis	Measurement property or study component	Analysis sample, *n*	Reported result	COSMIN rating	RoB rating
				b3=1.64966, b4=2.28172 19. EDDEP54*P* | slope=2.09932; b1=-0.14714, b2=0.52312, b3=1.68354, b4=2.37133 20. NQDEP01*N* | slope=2.64925; b1=0.30872, b2=0.89880, b3=1.88422, b4=2.55140 21. NQDEP08*N* | slope=1.39097; b1=-0.67682, b2=0.14277, b3=1.41130, b4=2.44408 22. NQDEP15 | slope=3.13286; b1=1.08723, b2=1.48687, b3=2.01529, b4=2.45718 23. NQDEP16 | slope=2.59718; b1=1.46551, b2=1.89301, b3=2.56600, b4=2.97717 24. NQDEP20*N* | slope=1.89448; b1=0.72376, b2=1.40645, b3=2.34282, b4=2.86678 25. NQDEP22 | slope=2.76672; b1=0.96544, b2=1.42641, b3=2.00417, b4=2.36611 26. NQDEP26*N* | slope=1.59980; b1=-0.28470, b2=0.63940, b3=1.96753, b4=3.14329 27. NQDEP29*N* | slope=2.19849; b1=0.48058, b2=1.08175, b3=1.96821, b4=2.70108 28. NQDEP30*N* | slope=1.92691; b1=-0.41660, b2=0.20044, b3=1.27276, b4=2.02554		
SCI-QOL Depression Item Bank (English)	Tulsky et al., 2015 - CFA, internal consistency, DIF, and PHQ-9 comparison	Internal consistency	716	Final 28-item bank: Cronbach's *alpha*=0.964; item-total correlations 0.51-0.81; marginal reliability=0.950. Because the CTT CFA met the COSMIN unidimensionality criterion and *alpha* was >=0.70, internal consistency was sufficient.	+	V
SCI-QOL Depression Item Bank (English)	Tulsky et al., 2015 - CFA, internal consistency, DIF, and PHQ-9 comparison	Cross-cultural validity/measurement invariance	716	DIF was evaluated for age, sex, education, injury level, injury completeness, and time since injury. The article states that 10 items were flagged using *chi-square P*<0.01 and McFadden pseudo-R2>0.02, described as small but non-negligible, but then states that all DIF was negligible and retained all items. Exact item-level R2 values were not reported. Applying the published >0.02 criterion strictly, the result was insufficient; the narrative contradiction was retained.	-	D
SCI-QOL Depression Item Bank (English)	Tulsky et al., 2015 - CFA, internal consistency, DIF, and PHQ-9 comparison	Hypotheses testing for construct validity	465	SCI-QOL Depression correlated *r*=0.76 with PHQ-9 in the nested crosswalk subsample *n*=465. The prespecified same-construct hypothesis *r*>=0.50 was met. Over half of linked and observed scores were within 0.5 *SD*, but 106 participants differed by >1 *SD*; the crosswalk is suitable for group-level comparison but not precise individual interchangeability. PHQ-9 1–4-coded raw score→SCI-QOL T-score (*SE*): 9→22 (9.85); 10→46 (3.50); 11→48 (2.50); 12→49 (2.00); 13→50 (1.70); 14→51 (1.48); 15→52 (1.32); 16→53 (1.19); 17→53 (1.09); 18→54 (1.00); 19→55 (0.92); 20→55 (0.86); 21→56 (0.80); 22→56 (0.75); 23→57 (0.71); 24→58 (0.67); 25→58 (0.63); 26→59 (0.59); 27→60 (0.56); 28→60 (0.54); 29→61 (0.51); 30→62 (0.49); 31→63 (0.47); 32→63 (0.45); 33→64 (0.43); 34→65 (0.41); 35→66 (0.40); 36→67 (0.38)	+	I
SCI-QOL Depression CAT (English)	Tulsky et al., 2015 - 1-2 week test-retest reliability	Reliability	245	Independent community sample *n*=245 completed the default variable-length CAT at baseline and 1-2 weeks. Pearson *r*=0.80 (*P*<0.001) and *ICC*(2,1)=0.80 (*95% CI* 0.75-0.84). The *ICC* met the COSMIN criterion >=0.70. Stability of depressive symptoms and equivalence of repeated administration conditions were not directly verified.	+	D
SCI-QOL Depression Short Form 10a (English)	Tulsky et al., 2015	No independent measurement-property evaluation	NA	The fixed 10-item form was selected from the calibrated SCI-QOL Depression item bank and a raw-score-to-T-score conversion table was provided. It was not administered or validated independently for any COSMIN measurement	NA	NA

PROM and version/form	Report or analysis	Measurement property or study component	Analysis sample, *n*	Reported result	COSMIN rating	RoB rating
				property in the included report.		
Patient Health Questionnaire-9, revised 3-category scoring (English)	Williams et al., 2016 - post hoc revised scoring	Structural validity	133 participants / 884 observations	After collapsing original categories 1 and 2, the revised 9-item score had person-separation reliability=0.77, zero misfitting items, point-measure correlations 0.44-0.73, variance explained 48.4% (eigenvalue 8.4), and first residual contrast 10.4% (eigenvalue 1.8). Local independence and monotonicity were not directly reported; optimization and evaluation used the same repeated observations without accounting for within-person dependence. Full COSMIN Rasch structural validity was indeterminate. the appendix retained all nine PHQ-9 items and collapsed original categories 1 ('several days') and 2 ('more than half the days') for every item; no PHQ-9 item misfit. Items: 1. Little interest or pleasure in doing things; 2. Feeling down, depressed, or hopeless; 3. Trouble falling or staying asleep, or sleeping too much; 4. Feeling tired or having little energy; 5. Poor appetite or overeating; 6. Feeling bad about yourself, or that you are a failure or have let yourself or your family down; 7. Trouble concentrating on things, such as reading the newspaper or watching television; 8. Moving or speaking slowly, or being unusually fidgety/restless; 9. Thoughts that you would be better off dead or of hurting yourself.	?	I
Patient Health Questionnaire-9, revised 3-category scoring (English)	Williams et al., 2016 - post hoc revised scoring	Internal consistency	133 participants / 884 observations	Cronbach's *alpha*=0.82 and person-separation reliability=0.77. Sufficient structural validity was not established for the revised version, so internal consistency was indeterminate.	?	I
Patient Health Questionnaire-9, revised 3-category scoring (English)	Williams et al., 2016 - post hoc revised scoring	Criterion validity	Exact week-12 *n* NR (maximum 133)	Week 12: continuous revised PHQ-9 with SCID-D MDD diagnosis *r*=0.79; 50%-response classification with SCID-D *r*=-0.86; PHQ remission <=4 with SCID-D *r*=-0.97. Absolute correlations exceeded 0.70, so criterion validity was sufficient. Exact analytic *n* was NR. the published table response-classification correlations: PHQ-HSCL 0.84, PHQ-HAM-D 0.89, HSCL-HAM-D 0.78; SCID-D with PHQ/HSCL/HAM-D response classifications = -0.86/-0.81/-0.70. PHQ remission (<=4) and HAM-D remission (<=7) correlated 0.84; their correlations with week-12 SCID-D MDD diagnosis were -0.97 and -0.98.	+	D
Patient Health Questionnaire-9, revised 3-category scoring (English)	Williams et al., 2016 - post hoc revised scoring	Hypotheses testing for construct validity	Exact week-12 *n* NR (maximum 133)	All eight prespecified convergent results met *r*>=0.50: HSCL 0.78, HAM-D 0.68, Maier 0.67, Bech 0.69, PGI 0.58, CGI 0.66, HSCL response classification 0.84 and HAM-D response classification 0.89. Complete week-12 the published table correlations: PHQ-HSCL 0.78; PHQ-HAM-D 0.68; PHQ-Maier 0.67; PHQ-Bech 0.69; HSCL-HAM-D 0.69; HSCL-Maier 0.72; HSCL-Bech 0.73; HAM-D-Maier 0.84; HAM-D-Bech 0.88; Maier-Bech 0.96; PGI with PHQ/HSCL/HAM-D/Maier/Bech = 0.58/0.58/0.58/0.60/0.62; CGI with PHQ/HSCL/HAM-D/Maier/Bech/PGI = 0.66/0.54/0.58/0.61/0.62/0.59; SCID-D with PHQ/HSCL/HAM-D/Maier/Bech/PGI/CGI = 0.79/0.82/0.91/0.87/0.85/0.74/0.69. HAM-D comparator details from the appendix: 1. Depressed Mood: collapse 1,2,3, not misfit; 2. Feelings of Guilt: collapse 1,2,3, not misfit; 3. Suicide: collapse 1 with 2 and 3 with 4, not misfit; 4. Insomnia Early: collapse 1 and 2, not misfit; 5. Insomnia Middle: collapse 1 and 2, not misfit; 6. Insomnia Late: collapse 1 and 2, not misfit; 7. Work and Activities: collapse 1,2,3, not misfit; 8. Retardation (Psychic and Motoric):	+	I

PROM and version/form	Report or analysis	Measurement property or study component	Analysis sample, *n*	Reported result	COSMIN rating	RoB rating
				collapse 1 with 2 and 3 with 4, misfit/deleted; 9. Agitation: collapse 1,2,3, misfit/deleted; 10. Anxiety Psychic: no revision, not misfit; 11. Anxiety Somatic: no revision, not misfit; 12. Somatic Symptoms - Gastrointestinal: collapse 1 and 2, not misfit; 13. Somatic Symptoms - General: no revision, not misfit; 14. Reduced Libido: collapse 1 and 2, misfit/deleted; 15. Hypochondriasis: no revision, misfit/deleted; 16. Weight Loss: collapse 1 and 2, misfit/deleted; 17. Insight: collapse 1 and 2, misfit/deleted. the published table HAM-D original/revised: 17/11 items; 886/886 observations; person-separation reliability 0.76/0.75; *alpha* 0.81/0.77; misfitting items 6/0; point-measure correlations 0.06-0.66/0.44-0.67; variance explained 43.2% (eigenvalue 12.9)/46.6% (9.6); first residual contrast 6.4% (1.9)/8.3% (1.7); floor 2.4%/3.6%.		
Patient Health Questionnaire-9, revised 3-category scoring (English)	Williams et al., 2016 - post hoc revised scoring	Responsiveness	133 participants / 884 observations	Prespecified hypothesis: treatment-control standardized mean-difference estimates should differ by <0.20 *SD*. PHQ active-treatment coefficient=-0.06 (*SE* 0.11, NS); comparators ranged -0.02 to -0.13, maximum difference 0.07 *SD*. The comparative hypothesis was met, although the PHQ treatment effect itself was not statistically significant. Complete the published table multilevel-growth coefficients (coefficient/*SE*/*t*/*p*): PHQ-9 intercept 0.72/0.08/8.49/<0.001, active treatment -0.06/0.11/-0.57/NS, week -0.21/0.02/-10.83/<0.001, week^2 0.01/0.002/5.88/<0.001; HSCL intercept 0.67/0.09/7.44/<0.001, active -0.02/0.12/-0.15/NS, week -0.19/0.02/-9.48/<0.001, week^2 0.01/0.00/4.85/<0.001; HAM-D intercept 0.60/0.09/6.54/<0.001, active -0.13/0.12/-1.09/NS, week -0.19/0.02/-9.75/<0.001, week^2 0.01/0.00/5.34/<0.001; Maier intercept 0.65/0.09/7.59/<0.001, active -0.12/0.11/-1.14/NS, week -0.21/0.02/-9.87/<0.001, week^2 0.01/0.00/5.38/<0.001; Bech intercept 0.66/0.09/7.63/<0.001, active -0.13/0.11/-1.15/NS, week -0.21/0.02/-10.34/<0.001, week^2 0.01/0.00/5.73/<0.001.	+	I
Hopkins Symptom Checklist-20 Depression Scale, Version B (English)	Williams et al., 2016 - original scoring	Structural validity	133 participants / 885 observations	Rasch analysis used 885 repeated observations. Response categories were underused and misordered; The tabulated result reports six misfitting items, point-measure correlations 0.13-0.66, variance explained 46.9% (eigenvalue 17.7), and first residual contrast 8.0% (eigenvalue 3.0). The Results text says seven items but lists six. Category disorder, item misfit and residual eigenvalue >2 support an insufficient result. the appendix applied the same category revision to all 20 original HSCL items: original categories 1 ('a little bit'), 2 ('moderately') and 3 ('quite a bit') were collapsed. Item/misfit status: 1. Feeling lonely or blue (retained according to the appendix); 2. Feeling hopeless about the future (retained according to the appendix); 3. Feeling no interest in things (retained according to the appendix); 4. Inability to take pleasure in things (retained according to the appendix); 5. Poor appetite (misfit/deleted); 6. Overeating (misfit/deleted); 7. Trouble falling asleep (misfit/deleted); 8. Awakening in the early morning (misfit/deleted); 9. Sleep that is restless or disturbed (misfit/deleted); 10. Thinking, speaking, and moving at a slower pace (retained according to the appendix); 11. Feeling so restless that you could not sit still (retained according to the appendix); 12. Thoughts of death or dying	-	I

PROM and version/form	Report or analysis	Measurement property or study component	Analysis sample, *n*	Reported result	COSMIN rating	RoB rating
				(retained according to the appendix); 13. Thoughts of ending your life (misfit/deleted); 14. Feeling low in energy or slowed down (retained according to the appendix); 15. Feeling everything is an effort (retained according to the appendix); 16. Blaming yourself for things (retained according to the appendix); 17. Feelings of worthlessness (retained according to the appendix); 18. Feelings of guilt (retained according to the appendix); 19. Trouble concentrating (retained according to the appendix); 20. Difficulty making decisions (retained according to the appendix). The Results text says seven items misfit but lists six; The tabulated result reports six misfitting items, and the appendix marks six. the published table nevertheless reports a 12-item revised score, whereas deleting six items would leave 14; the final 12-item composition cannot be reconstructed.		
Hopkins Symptom Checklist-20 Depression Scale, Version B (English)	Williams et al., 2016 - original scoring	Internal consistency	133 participants / 885 observations	Cronbach's *alpha*=0.91 and person-separation reliability=0.82. Because structural validity was insufficient, internal consistency was indeterminate despite high coefficients.	?	I
Hopkins Symptom Checklist revised 12-item depression scale (English)	Williams et al., 2016 - post hoc revised scoring	Structural validity	133 participants / 885 observations	The tabulated result reports a 12-item three-category score with person-separation reliability=0.84, zero misfitting items, point-measure correlations 0.42-0.72, variance explained 45.3% (eigenvalue 11.6), and first residual contrast 9.5% (eigenvalue 2.4). The residual eigenvalue exceeds the article's <2 criterion. the appendix marks only six misfitting/deleted items, which would leave 14 rather than 12; the revised version cannot be reconstructed. Structural validity was insufficient. the appendix applied the same category revision to all 20 original HSCL items: original categories 1 ('a little bit'), 2 ('moderately') and 3 ('quite a bit') were collapsed. Item/misfit status: 1. Feeling lonely or blue (retained according to the appendix); 2. Feeling hopeless about the future (retained according to the appendix); 3. Feeling no interest in things (retained according to the appendix); 4. Inability to take pleasure in things (retained according to the appendix); 5. Poor appetite (misfit/deleted); 6. Overeating (misfit/deleted); 7. Trouble falling asleep (misfit/deleted); 8. Awakening in the early morning (misfit/deleted); 9. Sleep that is restless or disturbed (misfit/deleted); 10. Thinking, speaking, and moving at a slower pace (retained according to the appendix); 11. Feeling so restless that you could not sit still (retained according to the appendix); 12. Thoughts of death or dying (retained according to the appendix); 13. Thoughts of ending your life (misfit/deleted); 14. Feeling low in energy or slowed down (retained according to the appendix); 15. Feeling everything is an effort (retained according to the appendix); 16. Blaming yourself for things (retained according to the appendix); 17. Feelings of worthlessness (retained according to the appendix); 18. Feelings of guilt (retained according to the appendix); 19. Trouble concentrating (retained according to the appendix); 20. Difficulty making decisions (retained according to the appendix). The Results text says seven items misfit but lists six; The tabulated result reports six misfitting items, and the appendix marks six. the published table nevertheless reports a 12-item revised score, whereas deleting six items would leave 14; the final 12-item composition cannot be	-	I

PROM and version/form	Report or analysis	Measurement property or study component	Analysis sample, *n*	Reported result	COSMIN rating	RoB rating
				reconstructed.		
Hopkins Symptom Checklist revised 12-item depression scale (English)	Williams et al., 2016 - post hoc revised scoring	Internal consistency	133 participants / 885 observations	Cronbach's *alpha*=0.90 and person-separation reliability=0.84. Structural validity was insufficient and retained-item composition unresolved; internal consistency was indeterminate.	?	I
Hopkins Symptom Checklist revised 12-item depression scale (English)	Williams et al., 2016 - post hoc revised scoring	Criterion validity	Exact week-12 *n* NR (maximum 133)	Week 12: continuous revised HSCL with SCID-D MDD diagnosis *r*=0.82 and 50%-response classification with SCID-D *r*=-0.81. Absolute correlations exceeded 0.70, so the reported criterion result was sufficient; applicability is limited by the unreproducible 12-item composition. the published table response-classification correlations: PHQ-HSCL 0.84, PHQ-HAM-D 0.89, HSCL-HAM-D 0.78; SCID-D with PHQ/HSCL/HAM-D response classifications = -0.86/-0.81/-0.70. PHQ remission (<=4) and HAM-D remission (<=7) correlated 0.84; their correlations with week-12 SCID-D MDD diagnosis were -0.97 and -0.98.	+	I
Hopkins Symptom Checklist revised 12-item depression scale (English)	Williams et al., 2016 - post hoc revised scoring	Hypotheses testing for construct validity	Exact week-12 *n* NR (maximum 133)	All eight prespecified convergent results met *r*>=0.50: PHQ-9 0.78, HAM-D 0.69, Maier 0.72, Bech 0.73, PGI 0.58, CGI 0.54, PHQ response classification 0.84 and HAM-D response classification 0.78. Complete week-12 the published table correlations: PHQ-HSCL 0.78; PHQ-HAM-D 0.68; PHQ-Maier 0.67; PHQ-Bech 0.69; HSCL-HAM-D 0.69; HSCL-Maier 0.72; HSCL-Bech 0.73; HAM-D-Maier 0.84; HAM-D-Bech 0.88; Maier-Bech 0.96; PGI with PHQ/HSCL/HAM-D/Maier/Bech = 0.58/0.58/0.58/0.60/0.62; CGI with PHQ/HSCL/HAM-D/Maier/Bech/PGI = 0.66/0.54/0.58/0.61/0.62/0.59; SCID-D with PHQ/HSCL/HAM-D/Maier/Bech/PGI/CGI = 0.79/0.82/0.91/0.87/0.85/0.74/0.69. HAM-D comparator details from the appendix: 1. Depressed Mood: collapse 1,2,3, not misfit; 2. Feelings of Guilt: collapse 1,2,3, not misfit; 3. Suicide: collapse 1 with 2 and 3 with 4, not misfit; 4. Insomnia Early: collapse 1 and 2, not misfit; 5. Insomnia Middle: collapse 1 and 2, not misfit; 6. Insomnia Late: collapse 1 and 2, not misfit; 7. Work and Activities: collapse 1,2,3, not misfit; 8. Retardation (Psychic and Motoric): collapse 1 with 2 and 3 with 4, misfit/deleted; 9. Agitation: collapse 1,2,3, misfit/deleted; 10. Anxiety Psychic: no revision, not misfit; 11. Anxiety Somatic: no revision, not misfit; 12. Somatic Symptoms - Gastrointestinal: collapse 1 and 2, not misfit; 13. Somatic Symptoms - General: no revision, not misfit; 14. Reduced Libido: collapse 1 and 2, misfit/deleted; 15. Hypochondriasis: no revision, misfit/deleted; 16. Weight Loss: collapse 1 and 2, misfit/deleted; 17. Insight: collapse 1 and 2, misfit/deleted. the published table HAM-D original/revised: 17/11 items; 886/886 observations; person-separation reliability 0.76/0.75; *alpha* 0.81/0.77; misfitting items 6/0; point-measure correlations 0.06-0.66/0.44-0.67; variance explained 43.2% (eigenvalue 12.9)/46.6% (9.6); first residual contrast 6.4% (1.9)/8.3% (1.7); floor 2.4%/3.6%.	+	I
Hopkins Symptom Checklist revised 12-item depression scale (English)	Williams et al., 2016 - post hoc revised scoring	Responsiveness	133 participants / 885 observations	Revised HSCL active-treatment coefficient=-0.02 (*SE* 0.12, NS); comparator estimates ranged -0.06 to -0.13, maximum absolute difference 0.11 *SD*, below the prespecified 0.20-*SD* threshold. The comparative hypothesis was met, although the HSCL treatment effect itself was not statistically significant.	+	I

PROM and version/form	Report or analysis	Measurement property or study component	Analysis sample, *n*	Reported result	COSMIN rating	RoB rating
				Complete the published table multilevel-growth coefficients (coefficient/*SE*/*t*/*p*): PHQ-9 intercept 0.72/0.08/8.49/<0.001, active treatment -0.06/0.11/-0.57/NS, week -0.21/0.02/-10.83/<0.001, week^2 0.01/0.002/5.88/<0.001; HSCL intercept 0.67/0.09/7.44/<0.001, active -0.02/0.12/-0.15/NS, week -0.19/0.02/-9.48/<0.001, week^2 0.01/0.00/4.85/<0.001; HAM-D intercept 0.60/0.09/6.54/<0.001, active -0.13/0.12/-1.09/NS, week -0.19/0.02/-9.75/<0.001, week^2 0.01/0.00/5.34/<0.001; Maier intercept 0.65/0.09/7.59/<0.001, active -0.12/0.11/-1.14/NS, week -0.21/0.02/-9.87/<0.001, week^2 0.01/0.00/5.38/<0.001; Bech intercept 0.66/0.09/7.63/<0.001, active -0.13/0.11/-1.15/NS, week -0.21/0.02/-10.34/<0.001, week^2 0.01/0.00/5.73/<0.001.		
PROMIS Depression Short Form 8b, version 1.0 (English)	Cook et al., 2017	Cross-cultural validity/measurement invariance	SCI 292; reference 761; total 1,053	Diagnosis-related DIF was evaluated by lordif ordinal logistic regression with a graded response model in SCI *n*=292 versus PROMIS community *n*=761. Combined-sample assumption checks were: *RMSEA*=0.116, *CFI*=0.993, *TLI*=0.990; EFA first-factor variance=74.6%, first/second eigenvalue ratio=18.3, factor correlation=0.930; no residual item pair exceeded 0.20. No PROMIS-D item met the study's standard meaningful-DIF criteria (>10% or >5% *beta* change, or McFadden delta-R2>0.05). At delta-R2>=0.005, items 1, 3 and 5 were flagged; item 1 showed non-uniform DIF and items 3 and 5 uniform DIF. DIF-corrected and uncorrected SCI scores correlated 0.998; difference range -1.77 to 1.64, mean -0.0341, *SD* 0.43448. Exact item-level McFadden delta-R2 values were not reported, so the COSMIN <0.02 criterion cannot be confirmed; the result was rated indeterminate.	?	I
Patient Health Questionnaire-9, Arabic version (Lebanese Arabic)	Summaka et al., 2019	Content validity - patient-rated comprehensibility	15	The translated questionnaire was pilot tested in 15 adults with SCI. Participants reported no problems understanding the items and completed the questionnaire in approximately 10 minutes. The pilot supported comprehensibility, although the small convenience sample and limited reporting resulted in doubtful methodological quality.	+	D
Patient Health Questionnaire-9, Arabic version (Lebanese Arabic)	Summaka et al., 2019 - factor analysis, internal consistency, retest reliability, ROC and convergent validity	Structural validity	51	PCA with varimax rotation in *n*=51: KMO=0.611; Bartlett *df*=36, *p*<0.001. Three factors had eigenvalues 3.15, 1.57 and 1.22 and explained 66.2% total variance. Factor 1 (27.06%): item 6 worthlessness/failure loading 0.841, communality 0.795; item 8 psychomotor change 0.823, communality 0.702; item 9 death/self-harm 0.827, communality 0.708. Factor 2 (21.05%): item 1 anhedonia 0.713, communality 0.696; item 2 depressed mood 0.532, communality 0.595; item 3 sleep 0.690, communality 0.503; item 4 fatigue 0.713, communality 0.556. Factor 3 (18.03%): item 5 appetite 0.854, communality 0.738; item 7 concentration 0.803, communality 0.661. The exploratory three-factor solution did not provide a confirmatory test of the intended total-score structure; the COSMIN result was therefore rated indeterminate. The first-to-second eigenvalue ratio was approximately 2.01, and no CFA was performed.	?	D
Patient Health Questionnaire-9, Arabic version	Summaka et al., 2019 - factor analysis, internal consistency, retest	Internal consistency	51	Cronbach's *alpha*=0.711 for the nine-item total score (*n*=51). Because sufficient unidimensional structural validity was not established, internal consistency was rated indeterminate under COSMIN.	?	A

PROM and version/form	Report or analysis	Measurement property or study component	Analysis sample, *n*	Reported result	COSMIN rating	RoB rating
(Lebanese Arabic)	reliability, ROC and convergent validity					
Patient Health Questionnaire-9, Arabic version (Lebanese Arabic)	Summaka et al., 2019 - factor analysis, internal consistency, retest reliability, ROC and convergent validity	Reliability	20	Retest *n*=20 after an average of 15 days: *ICC* average measure=0.886 (*95% CI* 0.711-0.955), *p*<0.001. The coefficient exceeded 0.70, but depression stability, repeated-test conditions, the *ICC* model, agreement-versus-consistency choice and the use of an average-measure rather than single-measure *ICC* were incompletely reported.	+	D
Patient Health Questionnaire-9, Arabic version (Lebanese Arabic)	Summaka et al., 2019 - factor analysis, internal consistency, retest reliability, ROC and convergent validity	Criterion validity	51	Against HDRS-A classification using cutoff 7.5, PHQ-9-A ROC *AUC*=0.83 (*95% CI* 0.72-0.94), *p*<0.001. The best PHQ-9-A cutoff was 7, sensitivity 83% and specificity 69%. HDRS-A is a clinician-rated depression-severity scale rather than an accepted diagnostic-interview gold standard; the criterion-validity result was therefore rated indeterminate.	?	I
Patient Health Questionnaire-9, Arabic version (Lebanese Arabic)	Summaka et al., 2019 - HDRS-A-defined known groups	Hypotheses testing for construct validity	51	HDRS-A-defined depressed participants had PHQ-9-A mean 11.8 (*SD* 5.2) versus 5.8 (*SD* 4.5) in non-depressed participants, *p*<0.001. Exact subgroup sizes and the statistical test were not reported; the directional known-groups hypothesis was met.	+	D
Patient Health Questionnaire-9, Arabic version (Lebanese Arabic)	Summaka et al., 2019 - factor analysis, internal consistency, retest reliability, ROC and convergent validity	Hypotheses testing for construct validity	51	PHQ-9-A and HDRS-A total scores: Spearman *r*=0.713, *p*<0.001, *n*=51. The prespecified same-construct hypothesis *r*>=0.50 was met.	+	A
Patient Health Questionnaire-2 (English)	Graves & Bombardier, 2008	Structural validity	3652	The two-item configuration comprised PHQ-9 items 1 and 2 and was analysed with a two-parameter graded-response model in the same *n*=3,652 records. Relative efficiency was 0.46 versus the full PHQ-9, and the two-item and full scales reached peak information at approximately theta=1.4. No separate dimensionality test, item-fit statistics, monotonicity, local independence or model-fit results were reported; structural validity was indeterminate.	?	D
Patient Health Questionnaire-2 (English)	Poritz et al., 2018	Internal consistency	116	Cronbach's *alpha*=0.91 in *n*=116. PHQ-2 was independently administered rather than calculated from PHQ-9 responses. No sufficient structural-validity evidence for PHQ-2 was provided in this report.	?	V
Patient Health Questionnaire-2 (English)	Bombardier et al., 2012	Criterion validity	142	PHQ-9 was interview-administered by trained research assistants. The SCID DSM-IV MDD module was administered to all participants regardless of PHQ-9 score by trained mental-health professionals with SCI experience who were blinded to PHQ-9 results. Mean interval between PHQ-9 and SCID was 3.1 days (*SD* 2.2), and the final analysis required an interval <=7 days. Complete the published table PHQ-2/cardinal-symptom results against SCID MDD (*N*=142): PHQ-2 >=1: 81 (57%), sensitivity 1.00 (*95% CI* 0.78-1.00), specificity 0.48 (0.39-0.56), Youden 0.48, *PPV* 0.17 (0.10-0.27), *NPV* 1.00 (0.94-1.00), *LR*+ 1.91 (1.62-2.25), *LR*- 0, kappa 0.15 (0.07-0.23); PHQ-2 >=2: 55 (39%), sensitivity 0.93 (0.69-0.99), specificity 0.67 (0.59-0.75), Youden 0.60, *PPV* 0.24 (0.13-0.37), *NPV* 0.99 (0.94-1.00), *LR*+ 2.83 (2.12-3.77), *LR*- 0.11 (0.02-0.71), kappa 0.26 (0.13-0.39); PHQ-2 >=3: 29 (20%), sensitivity 0.57 (0.33-0.79), specificity 0.84 (0.76-0.89), Youden 0.41, *PPV* 0.28 (0.13-0.47), *NPV* 0.95 (0.89-0.98), *LR*+ 3.48 (1.91-6.34), *LR*- 0.51 (0.28-0.94), kappa	-	A

PROM and version/form	Report or analysis	Measurement property or study component	Analysis sample, *n*	Reported result	COSMIN rating	RoB rating
				0.28 (0.08-0.47); at least one cardinal symptom >=2: 35 (25%), sensitivity 0.64 (0.39-0.84), specificity 0.80 (0.72-0.86), Youden 0.44, *PPV* 0.26 (0.12-0.43), *NPV* 0.95 (0.89-0.98), *LR+* 3.17 (1.88-5.32), *LR-* 0.45 (0.22-0.91), kappa 0.26 (0.09-0.44). No PHQ-2 cutoff achieved both sensitivity and specificity above 0.80; criterion validity was insufficient for stand-alone diagnosis, although >=1 had 100% sensitivity and was proposed as the first step in a two-stage screen. The article contains participant-flow inconsistencies: the abstract reports 204 eligible patients, whereas Results reports 205 enrolled; Methods states *n*=37 were excluded because PHQ-9 and SCID could not be completed within 7 days, but 173 received SCID and 142 entered the final analysis, implying 31 with SCID were outside the paired-analysis window. The final 142 were broadly comparable with excluded/nonpaired participants on sex, tetraplegia, age, days since injury and PHQ-9 score, with a borderline racial-distribution difference (*p*=0.051).		
Patient Health Questionnaire-2 (English)	Poritz et al., 2018	Criterion validity	116	PHQ-9 score >=10 was used as a proxy for clinically significant depressive symptoms, not an independent diagnostic gold standard. Estimated prevalence was 20.7%. PHQ-2 mean=1.17 (*SD* 1.948); PHQ-9 mean=5.23 (*SD* 7.451). ROC *AUC* for PHQ-2=0.979. Complete reported results: cutoff >=1, classification accuracy 84.5%, sensitivity 100%, specificity 80.4%, *PPV* 57.1%, *NPV* 100%; cutoff >=2, accuracy 92.2%, sensitivity 95.8%, specificity 91.3%, *PPV* 74.2%, *NPV* 98.8%; cutoff >=3, accuracy 94.8%, sensitivity 83.3%, specificity 97.8%, *PPV* 90.9%, *NPV* 95.7%; cutoff >=4, accuracy 93.1%, sensitivity 70.8%, specificity 98.9%, *PPV* 94.4%, *NPV* 92.9%; cutoff >=5, accuracy 89.7%, sensitivity 54.2%, specificity 98.9%, *PPV* 92.9%, *NPV* 89.2%. Cutoff >=3 maximized overall classification accuracy. Because PHQ-9 >=10 is a PROM-based proxy and shares the same symptom content, these results do not establish criterion validity against a diagnostic interview.	?	I
PHQ-9-derived 3-item short form (English)	Graves & Bombardier, 2008	Structural validity	3652	The post hoc three-item configuration comprised PHQ-9 items 1, 2 and 6, selected because their one-factor CFA loadings were 0.72, 0.79 and 0.73. In the same *n*=3,652 sample, graded-response-model relative efficiency was 0.67 in the Results and 0.66 in the abstract/conclusion. The three-item and full scales reached peak information at approximately theta=1.4. No separate dimensionality test, item-fit statistics, monotonicity, local independence or model-fit results were reported, and the short form was selected and evaluated in the same dataset; structural validity was indeterminate.	?	D
PHQ-9-derived 3-item short form (English)	Graves & Bombardier, 2008	Cross-cultural validity/measurement invariance	Men 2,863; women 789	For the three-item form, relative efficiency versus the total sample was 1.02 for men and 0.93 for women; women versus men=0.90, suggesting a smaller cumulative sex difference than for the full PHQ-9. No individual item-parameter difference was statistically significant. No recognized multi-group CFA, formal item-level DIF test or COSMIN-compatible effect-size criterion was reported; measurement invariance was indeterminate.	?	I
PHQ-9-derived 3-item short form (English)	Graves & Bombardier, 2008	Criterion validity	3652	The squared correlation between three-item and full PHQ-9 total scores was 0.794. Parent PHQ-9 score >=10 classified 566/3,652 (15.5%) as probable major depression. Three-item cutoff >=3 classified 941 (25.8%): TP=528, FN=38, FP=413, TN=2,673; sensitivity=0.87, specificity=0.93, *PPV*=0.56.	?	I

PROM and version/form	Report or analysis	Measurement property or study component	Analysis sample, *n*	Reported result	COSMIN rating	RoB rating
				Cutoff >=4 classified 603 (16.5%): TP=462, FN=104, FP=141, TN=2,945; sensitivity=0.82, specificity=0.95, *PPV*=0.77. Because the criterion was the overlapping parent PHQ-9 rather than an independent diagnostic interview, these data represent parent-scale agreement and screening interpretability, not sufficient criterion validity.		
SCL-90-R Cognitive/Affective Depression Research Subscale (English)	Buckelew et al., 1988 - SCI internal consistency and known-groups validity	Internal consistency	52	SCI-specific Cronbach's *alpha*=.89 (*n*=52). Cognitive-subscale alphas were .89 in SCI, .91 in chronic pain, .91 in college controls, and .91 in the total sample. In the pooled sample, the average inter-item correlation was .43 (range .26-.70) and the item-total range was .58-.81. Complete Reported item-total correlations in the order SCI/pain/college/all groups: item 15=.55/.63/.67/.64; 10=.48/.64/.55/.58; 22=.63/.61/.70/.65; 26=.65/.58/.66/.63; 29=.67/.78/.66/.70; 30=.81/.81/.73/.78; 31=.74/.74/.66/.71; 32=.86/.70/.67/.71; 54=.80/.79/.76/.78; 71=.55/.70/.71/.67; 79=.77/.83/.80/.81; 59=.51/.64/.66/.64; 89=.68/.63/.71/.68. No structural-validity analysis of this 13-item subscale was performed in the article; internal consistency was therefore indeterminate under COSMIN despite *alpha*>=.70.	?	V
SCL-90-R Cognitive/Affective Depression Research Subscale (English)	Buckelew et al., 1988 - SCI internal consistency and known-groups validity	Hypotheses testing for construct validity	SCI 52; total 275	Three-group MANOVA (SCI *n*=52, chronic pain *n*=78, college controls *n*=145) found a diagnosis main effect, Wilks *F*(4,536)=21, *p*<.0001; sex *F*(2,268)=.71, *p*=.49 and diagnosis-by-sex interaction *F*(4,536)=.15, *p*=.97 were not significant. Standard SCL-90-R Depression T scores were pain 64.5, SCI 61.7 and college 64.2, *F*(2,272)=1.86, *p*=.16. Exact cognitive and somatic group means were shown only graphically in the published figure and were not tabulated. Cognitive-depression group effect *F*(2,269)=4.05, *p*<.01; Duncan post hoc tests showed that the SCI sample reported significantly less cognitive depression than both the chronic-pain and college-control groups, *p*<.05. In hierarchical regression restricted to the two rehabilitation groups, disability duration was not significant and diagnosis remained significant, *F*(1,124)=10.49, *p*<.001.	+	D
SCL-90-R Cognitive/Affective Depression Research Subscale (English)	Buckelew et al., 1988 - pooled convergent/divergent correlations; not SCI-specific	Hypotheses testing for construct validity	Pooled *n*=275; SCI-specific unavailable	Pooled across SCI, chronic-pain and college samples (total *n*=275), not separately extractable for SCI: cognitive depression correlations with SCL-90-R Somatization=.54, Obsessive-Compulsive=.78, Interpersonal Sensitivity=.71, Anxiety=.77, Hostility=.57, Phobic Anxiety=.50, Paranoid Ideation=.68 and Psychoticism=.74; somatic depression correlations with the same scales=.66, .31, .57, .53, .32, .33, .35 and .47. Cognitive and somatic depression correlated .56. These pooled correlations were documented but excluded from the SCI-specific COSMIN synthesis.	NA	NA
SCL-90-R Somatic Depression Research Subscale (English)	Buckelew et al., 1988 - SCI internal consistency and known-groups validity	Internal consistency	52	SCI-specific Cronbach's *alpha*=.62 (*n*=52). Somatic-subscale alphas were .62 in SCI, .70 in chronic pain, .63 in college controls, and .69 in the total sample. In the pooled sample, the average inter-item correlation was .25 (range -.22 to .70) and the item-total range was .29-.80. Complete Reported item-total correlations in the order SCI/pain/college/all groups: item 5=.48/.57/.35/.52; 14=.65/.68/.48/.61; 19=.42/.50/.57/.46; 60=.25/.23/.45/.29; 44=.76/.65/.75/.76; 64=.62/.67/.52/.66; 66=.63/.80/.75/.80. Item 60 (overeating) had the weakest SCI item-total correlation (.25) and the authors recommended omitting it, but they did not recalculate or validate a six-item version. No structural-validity	?	V

PROM and version/form	Report or analysis	Measurement property or study component	Analysis sample, *n*	Reported result	COSMIN rating	RoB rating
				analysis of the seven-item subscale was performed; internal consistency was therefore indeterminate under COSMIN.		
SCL-90-R Somatic Depression Research Subscale (English)	Buckelew et al., 1988 - SCI internal consistency and known-groups validity	Hypotheses testing for construct validity	SCI 52; total 275	Three-group MANOVA (SCI *n*=52, chronic pain *n*=78, college controls *n*=145) found a diagnosis main effect, Wilks $F(4,536)=21$, $p<.0001$; sex $F(2,268)=.71$, $p=.49$ and diagnosis-by-sex interaction $F(4,536)=.15$, $p=.97$ were not significant. Standard SCL-90-R Depression T scores were pain 64.5, SCI 61.7 and college 64.2, $F(2,272)=1.86$, $p=.16$. Exact cognitive and somatic group means were shown only graphically in the published figure and were not tabulated. Somatic-depression group effect $F(2,269)=28.73$, $p<.0001$; Duncan post hoc tests showed that chronic-pain patients reported significantly more somatic depression than both the SCI and college-control groups, $p<.05$. No significant SCI-versus-college difference was reported. In hierarchical regression restricted to the two rehabilitation groups, disability duration was not significant and diagnosis remained significant, $F(1,124)=24.34$, $p<.0001$.	-	D
SCL-90-R Somatic Depression Research Subscale (English)	Buckelew et al., 1988 - pooled convergent/divergent correlations; not SCI-specific	Hypotheses testing for construct validity	Pooled *n*=275; SCI-specific unavailable	Pooled across SCI, chronic-pain and college samples (total *n*=275), not separately extractable for SCI: cognitive depression correlations with SCL-90-R Somatization=.54, Obsessive-Compulsive=.78, Interpersonal Sensitivity=.71, Anxiety=.77, Hostility=.57, Phobic Anxiety=.50, Paranoid Ideation=.68 and Psychoticism=.74; somatic depression correlations with the same scales=.66, .31, .57, .53, .32, .33, .35 and .47. Cognitive and somatic depression correlated .56. These pooled correlations were documented but excluded from the SCI-specific COSMIN synthesis.	NA	NA
Inventory to Diagnose Depression total severity score (English)	Frank et al., 1992	Structural validity	134	Separate 22-item exploratory factor analyses were conducted in SCI *n*=134, rheumatoid arthritis *n*=78, students *n*=140 and community participants *n*=150 using unweighted least-squares extraction and varimax rotation. KMO values ranged .80-.82. Kaiser-Guttman suggested at least six factors; the scree test suggested four to five, and a four-factor solution was selected for interpretability. Loading cutoff was |.35|. The four-factor solutions accounted for an average 54% of common variance across groups; the first factor accounted for 33%. Skew-sensitive reanalysis and four random 25%-deletion replications yielded mean salient-variable indices >.70 for at least three factors. The authors explicitly stated that the main factor did not make the IDD unidimensional. Because the IDD total severity score is interpreted as one score but a four-factor structure emerged and the first factor explained only 33%, structural validity of the total score was insufficient. SCI dysphoria-factor loadings for IDD items 1-22 were: low mood .38; decreased energy -.05; psychomotor agitation .15; psychomotor retardation .12; loss of interest .34; loss of pleasure .18; loss of libido .06; guilt .53; worthlessness .42; suicidal ideation .49; concentration .18; indecisiveness .33; decreased appetite .34; weight loss .12; increased appetite .10; weight gain -.05; insomnia .24; hypersomnia .05; anxiety .49; hopelessness .81; irritability .34; somatic complaints .44.	-	A
Inventory to Diagnose Depression total severity score (English)	Frank et al., 1992	Cross-cultural validity/measurement invariance	Total 502; SCI 134	Four-factor patterns were compared across SCI, rheumatoid arthritis, student and community groups using the salient-variable index. The common dysphoria factor had mean S>.83 overall, with S>.70 in SCI and S>.71 in	?	D

PROM and version/form	Report or analysis	Measurement property or study component	Analysis sample, *n*	Reported result	COSMIN rating	RoB rating
				rheumatoid arthritis; replication was stronger in students (>1.0) and community (>.90). This was not a recognized multigroup CFA or item-level DIF analysis, and groups differed substantially in age and clinical status; measurement invariance was indeterminate.		
Inventory to Diagnose Depression total severity score (English)	Frank et al., 1992	Hypotheses testing for construct validity	SCI recruitment *n*=134; the four-group ANOVA reported *df*=3,452, implying total analyzable *n*=456. Group-specific complete-case denominators were not reported.	IDD total-severity means were rheumatoid arthritis 16.4, SCI 13.3, students 12.1 and community 9.4; *F*(3,452)=8.01, *p*<.0001. Scheffe groupings were A, AB, B and B, respectively. Thus the SCI mean was not significantly different from the community or student means, so the prespecified SCI-versus-community known-groups hypothesis was not supported. Reported DSM-III-R MDD was 13 SCI participants (11%), 8 rheumatoid arthritis (14%), 7 students (5.7%) and 6 community (4%); *chi-square*=12.73, *df*=3, *p*<.05. The count 13/134 does not equal 11%, implying a nonmissing denominator that was not reported.	-	D
Inventory to Diagnose Depression Dysphoria factor score (English)	Frank et al., 1992	Structural validity	134	A nine-item common dysphoria factor was identified across at least three of four populations, but it was selected post hoc from the same exploratory analyses and was not subjected to a separate unidimensional CFA or complete item-fit analysis. In the SCI solution, several proposed common-factor items had loadings below .35 (loss of pleasure .18, indecisiveness .33, irritability .34). Structural validity of the derived score was therefore indeterminate. SCI dysphoria-factor loadings for IDD items 1-22 were: low mood .38; decreased energy -.05; psychomotor agitation .15; psychomotor retardation .12; loss of interest .34; loss of pleasure .18; loss of libido .06; guilt .53; worthlessness .42; suicidal ideation .49; concentration .18; indecisiveness .33; decreased appetite .34; weight loss .12; increased appetite .10; weight gain -.05; insomnia .24; hypersomnia .05; anxiety .49; hopelessness .81; irritability .34; somatic complaints .44.	?	D
Inventory to Diagnose Depression Dysphoria factor score (English)	Frank et al., 1992	Cross-cultural validity/measurement invariance	Total 502; SCI 134	Four-factor patterns were compared across SCI, rheumatoid arthritis, student and community groups using the salient-variable index. The common dysphoria factor had mean S>.83 overall, with S>.70 in SCI and S>.71 in rheumatoid arthritis; replication was stronger in students (>1.0) and community (>.90). This was not a recognized multigroup CFA or item-level DIF analysis, and groups differed substantially in age and clinical status; measurement invariance was indeterminate.	?	D
Inventory to Diagnose Depression Dysphoria factor score (English)	Frank et al., 1992	Hypotheses testing for construct validity	SCI recruitment *n*=134; the four-group ANOVA reported *df*=3,452, implying total analyzable *n*=456. Group-specific complete-case denominators were not reported.	Dysphoria factor-severity means were rheumatoid arthritis 7.17, SCI 5.30, students 6.10 and community 4.74; *F*(3,452)=3.55, *p*<.01. Scheffe groupings were A, A, A and B, respectively, so the SCI score was significantly higher than the community score and the prespecified known-groups hypothesis was supported.	+	D
Brief Symptom Inventory SCI-specific Depression Research Factor (7-item English)	Heinrich & Tate, 1996	Structural validity	215	The seven-item SCI Depression factor was derived post hoc from the same full-BSI exploratory analysis. Although all seven items loaded >=.50 in the principal-components solution, the maximum-likelihood solution did not reproduce a single identical depression factor and no independent CFA was performed. Structural validity was therefore indeterminate. Principal-	?	I

PROM and version/form	Report or analysis	Measurement property or study component	Analysis sample, *n*	Reported result	COSMIN rating	RoB rating
				components and maximum-likelihood exploratory analyses were run on all 53 BSI items (*n*=215; 4.06 participants/item) with oblique rotation. Models with 3-14 factors were examined. Factors were retained using substantive interpretability, >=5% variance, >=5 items and loading >=.35. The principal-components solution identified seven factors explaining 52.5% of variance; 45/53 items loaded >=.35. A general-somatization factor had *alpha*=.64 and was dropped, leaving six recommended research factors. The unrotated first factor explained 27%. Depression accounted for 9.6% and *alpha*=.88. Maximum-likelihood estimation gave a less stable depression solution, splitting depression into two subscales. Factor intercorrelations for Depression were .36 with Anxiety, .24 with Mental Blocks, .23 with Interpersonal Sensitivity, .13 with SCI and .25 with Hostile Suspiciousness. Seven-item Depression research factor: item 35 feeling hopeless about the future loading .74; item 18 feeling no interest in things .68; item 9 thoughts of ending your life .64; item 17 feeling blue .61; item 14 feeling lonely even when with people .60; item 16 feeling lonely .56; item 50 feelings of worthlessness .50.		
Brief Symptom Inventory SCI-specific Depression Research Factor (7-item English)	Heinrich & Tate, 1996	Internal consistency	215	Cronbach's *alpha*=.88 for the seven-item SCI Depression factor (*n*=215). Because sufficient unidimensional structural validity was not established independently, internal consistency was rated indeterminate under COSMIN.	?	V
Brief Symptom Inventory SCI-specific Depression Research Factor (7-item English)	Heinrich & Tate, 1996	Hypotheses testing for construct validity	Follow-up *n*=204	Among participants with follow-up data, the rehospitalized group (*n*=51) had a baseline SCI Depression factor mean .83 (*SD* .95) versus .51 (*SD* .65) in the non-rehospitalized group (*n*=153), *t*=2.28, *p*<.01. The prespecified directional hypothesis was supported. Outcome ascertainment omitted rehospitalizations outside the study system and multiple subscales were tested without a reported multiplicity adjustment.	+	D

Table SVIA. Complete COSMIN-modified GRADE downgrading decisions for summarized measurement-property results

All bodies of evidence started at high certainty. Downgrading was considered for risk of bias, inconsistency, imprecision, and indirectness. Publication bias was not assessed. Imprecision was not separately graded for content validity, structural validity, or cross-cultural validity/measurement invariance. The final certainty levels reproduce the consensus ratings in the official COSMIN review-management workbook. For internal consistency, certainty was capped at the certainty of the corresponding structural-validity evidence.

Downgrade notation: 0, no serious concern; −1, serious; −2, very serious; −3, extremely serious; NA, not separately assessed under COSMIN. Ratings: +, sufficient; −, insufficient. The total downgrade is capped at very low certainty.

For bodies containing overlapping, nested, matched, or repeated-measures samples, participant counts were not arithmetically pooled. Only evidence contributing to the summarized rating was considered in the certainty assessment.

PROM and version/form	Measurement property	Overall rating	Evidence base used for certainty assessment	Risk of bias	Inconsistency	Imprecision	Indirectness	Final certainty	Complete downgrading rationale
CES-D-20 (English)	Structural validity	-	McColl & Skinner, 1992; *n*=120; one adequate-quality CFA/PCA study.	−1 Serious	0	NA	0	Moderate	A single adequate-quality study supported the summarized insufficient rating. Imprecision is not separately graded for structural validity.
CES-D-20 (English)	Reliability	+	Miller et al., 2008; *n*=47; ICC(1,1)=0.87.	−1 Serious	0	−2 Very serious	0	Very low	One adequate-quality study and a total sample below 50 produced serious RoB and very serious imprecision.
CES-D-20 (English)	Construct validity – hypotheses testing	+	McColl & Skinner, 1992 (*n*=120) and Miller et al., 2008 (*n*=47); two interpretable hypotheses supported; one SF-36 result set indeterminate.	−3 Extremely serious	0	0	0	Very low	All interpretable evidence came from inadequate-quality analyses; the indeterminate SF-36 result was not used to overturn the sufficient summary.
Zung Self-Rating Depression Scale (English)	Structural validity	-	Tate et al., 1993; *n*=162; one doubtful-quality exploratory factor analysis.	−2 Very serious	0	NA	0	Low	A single doubtful-quality study supported the insufficient rating. Imprecision is not separately graded for structural validity.
Zung Self-Rating Depression Scale (English)	Construct validity – hypotheses testing	+	Tate et al., 1993 (*n*=162) and Campagnolo et al., 2002 (*n*=39/59); 8/8 hypotheses supported.	−2 Very serious	0	0	0	Low	Evidence combined inadequate, doubtful, and adequate analyses, with most support coming from lower-quality methods; the total evidence base exceeded 100 participants.
Brief Symptom Inventory – Depression subscale (English)	Structural validity	-	Tate et al., 1993 (*n*=162) and Heinrich & Tate, 1996 (*n*=215); the definite insufficient result came from the former, while the latter was indeterminate.	−3 Extremely serious	0	NA	0	Very low	The summarized insufficient rating depended on one inadequate-quality structural analysis; the second inadequate analysis was indeterminate. Imprecision is not separately graded for structural validity.

PROM and version/form	Measurement property	Overall rating	Evidence base used for certainty assessment	Risk of bias	Inconsistency	Imprecision	Indirectness	Final certainty	Complete downgrading rationale
Brief Symptom Inventory – Depression subscale (English)	Construct validity – hypotheses testing	+	Tate et al., 1990/1993, Mitchell et al., 2008, and Heinrich & Tate, 1996; 5/6 evaluable hypotheses supported.	−2 Very serious	−1 Serious	0	0	Very low	Multiple inadequate or doubtful analyses and one unsupported rehospitalization hypothesis required downgrading for RoB and inconsistency.
Ilfeld Psychiatric Symptom Index - Depression subscale (English)	Construct validity – hypotheses testing	+	Campagnolo et al., 2002; SCI-specific analyses *n*=39 and *n*=59 from one cohort; 2/2 hypotheses supported.	−2 Very serious	0	−1 Serious	0	Very low	Evidence came from one study with one inadequate and one adequate analysis; the unique SCI sample was below 100.
Patient Health Questionnaire-9 (English)	Structural validity	-	Ten principal structural-analysis units across Graves & Bombardier, Kalpakjian, Krause, Richardson & Richards, and Williams; *n*=202–7,296; possible cohort overlap considered.	0	0	NA	0	High	Multiple large, predominantly very-good-quality analyses directly addressed the English PHQ-9. Eight of ten principal units were insufficient; the two sufficient and one indeterminate analyses were explained by model and method differences, so no unexplained inconsistency downgrade was applied.
Patient Health Questionnaire-9 (English)	Criterion validity	+	Bombardier et al., 2012; *n*=142; blinded SCID comparison; AUC=0.921.	−1 Serious	0	0	0	Moderate	One adequate-quality diagnostic-accuracy study; sample size exceeded 100.
Patient Health Questionnaire-9 (English)	Construct validity – hypotheses testing	+	Bombardier et al., 2004/2012, Richardson & Richards, 2008, Krause et al., 2009, and Tulsky et al., 2015; 15/19 hypotheses supported; overlapping cohorts considered.	−1 Serious	−1 Serious	0	0	Low	Most analyses were doubtful, with some adequate analyses; the sufficient summary used a ≥75% majority despite four unsupported hypotheses, requiring one-level downgrades for RoB and inconsistency.
Hospital Anxiety and Depression Scale - Depression subscale (English)	Structural validity	+	Woolrich et al., 2006; *n*=963; one large two-factor PCA study.	−1 Serious	0	NA	0	Moderate	The single study had reporting and method limitations judged serious but not sufficient for a two-level downgrade; imprecision is not separately graded for structural validity.

PROM and version/form	Measurement property	Overall rating	Evidence base used for certainty assessment	Risk of bias	Inconsistency	Imprecision	Indirectness	Final certainty	Complete downgrading rationale
Hospital Anxiety and Depression Scale - Depression subscale (English)	Internal consistency	+	Woolrich et al., 2006; *n*=963; Cronbach's alpha=0.79, with sufficient structural evidence for the depression subscale.	0	0	0	0	Moderate	The internal-consistency analysis was very-good quality and large, with no serious concern in the four GRADE domains. Under COSMIN, however, certainty for internal consistency cannot exceed the certainty of the corresponding sufficient structural-validity evidence; it was therefore capped at Moderate.
Hospital Anxiety and Depression Scale - Depression subscale (English)	Construct validity – hypotheses testing	+	Woolrich et al., 2006; maximum *n*=963; 1/1 hypothesis supported.	−1 Serious	0	0	0	Moderate	One large study with doubtful methodological quality; no imprecision downgrade.
CES-D-10 (English)	Reliability	+	Miller et al., 2008; *n*=47; ICC(1,1)=0.85.	−1 Serious	0	−2 Very serious	0	Very low	One adequate-quality study and a sample below 50.
Depression Anxiety Stress Scales-21 - Depression subscale (English)	Construct validity – hypotheses testing	+	Mitchell et al., 2008; *n*=40; 1/1 hypothesis supported.	−3 Extremely serious	0	0	0	Very low	The only contributing construct-validity analysis was inadequate; the certainty floor was therefore very low.
Older Adult Health and Mood Questionnaire (English)	Construct validity – hypotheses testing	+	Krause et al., 2009; two analyses from the same cohort (reported as *r*(655) and *r*(671)); 2/2 hypotheses supported.	−3 Extremely serious	0	0	0	Very low	Evidence came from one report with inadequate/doubtful analyses and no independent replication.
SCI-QOL Depression Item Bank (English)	Content validity – relevance	+	Tulsky et al., 2015; SCI-specific PROM-development programme; development sample details incompletely reported.	−2 Very serious	0	NA	0	Low	One doubtful-quality PROM-development study; no separate patient or professional content-validity study. Sample size is incorporated in content-validity RoB and was not graded again for imprecision.
SCI-QOL Depression Item Bank (English)	Content validity – comprehensiveness	+	Tulsky et al., 2015; SCI-specific concept elicitation, expert review, and cognitive debriefing.	−2 Very serious	0	NA	0	Low	One doubtful-quality development study; qualitative sampling and saturation procedures were incompletely reported. No separate content-validity study was available.

PROM and version/form	Measurement property	Overall rating	Evidence base used for certainty assessment	Risk of bias	Inconsistency	Imprecision	Indirectness	Final certainty	Complete downgrading rationale
SCI-QOL Depression Item Bank (English)	Structural validity	+	Tulsky et al., 2015; *n*=716; adequate CFA was sufficient; the graded-response IRT analysis was doubtful and indeterminate.	−1 Serious	0	NA	0	Moderate	One adequate sufficient CFA plus one doubtful indeterminate IRT analysis from the same calibration sample; no imprecision grading for structural validity.
SCI-QOL Depression Item Bank (English)	Internal consistency	+	Tulsky et al., 2015; *n*=716; alpha=0.964 and marginal reliability=0.950.	0	0	0	0	Moderate	The internal-consistency analysis was very-good quality and large, with no serious concern in the four GRADE domains. Under COSMIN, however, certainty for internal consistency cannot exceed the certainty of the corresponding sufficient structural-validity evidence; it was therefore capped at Moderate.
SCI-QOL Depression Item Bank (English)	Cross-cultural validity/measurement invariance	-	Tulsky et al., 2015; *n*=716; DIF by age, sex, education, injury characteristics, and time since injury.	−2 Very serious	0	NA	0	Low	One doubtful-quality DIF analysis with contradictory reporting and incomplete effect-size interpretation. Imprecision is not separately graded for cross-cultural validity/measurement invariance.
SCI-QOL Depression Item Bank (English)	Construct validity – hypotheses testing	+	Tulsky et al., 2015; *n*=465; correlation with PHQ-9 *r*=0.76; 1/1 hypothesis supported.	−3 Extremely serious	0	0	0	Very low	The sole contributing analysis was rated inadequate, yielding very-low certainty despite the large sample.
SCI-QOL Depression CAT (English)	Reliability	+	Tulsky et al., 2015; independent retest *n*=245; ICC(2,1)=0.80.	−2 Very serious	0	0	0	Low	One doubtful-quality reliability study; sample size exceeded 100.
Patient Health Questionnaire-9, revised 3-category scoring (English)	Criterion validity	+	Williams et al., 2016; post hoc scoring; exact week-12 *n* not reported, maximum *n*=133.	−2 Very serious	0	0	0	Low	One doubtful-quality post hoc analysis; no additional imprecision downgrade was applied because the final analytic *n* was not reported but could be above 100.
Patient Health Questionnaire-9, revised 3-category scoring (English)	Construct validity – hypotheses testing	+	Williams et al., 2016; post hoc scoring; maximum *n*=133; 8/8 hypotheses	−3 Extremely serious	0	0	0	Very low	The only contributing analysis was inadequate and used a scoring form derived and

PROM and version/form	Measurement property	Overall rating	Evidence base used for certainty assessment	Risk of bias	Inconsistency	Imprecision	Indirectness	Final certainty	Complete downgrading rationale
			supported.						evaluated in the same sample.
Patient Health Questionnaire-9, revised 3-category scoring (English)	Responsiveness	+	Williams et al., 2016; 133 participants/884 observations; treatment-effect differences ≤0.07 *SD*.	−3 Extremely serious	0	0	0	Very low	One inadequate post hoc repeated-measures analysis; repeated observations did not constitute independent participants.
Hopkins Symptom Checklist-20 Depression Scale, Version B (English)	Structural validity	-	Williams et al., 2016; 133 participants/885 observations; Rasch analysis.	−3 Extremely serious	0	NA	0	Very low	The only structural analysis was inadequate; repeated observations were not independent. Imprecision is not separately graded for structural validity.
Hopkins Symptom Checklist revised 12-item depression scale (English)	Structural validity	-	Williams et al., 2016; 133 participants/885 observations; post hoc 12-item form.	−3 Extremely serious	0	NA	0	Very low	One inadequate post hoc structural analysis; the final item set was not fully reconstructable. Imprecision is not separately graded for structural validity.
Hopkins Symptom Checklist revised 12-item depression scale (English)	Criterion validity	+	Williams et al., 2016; post hoc form; exact week-12 *n* not reported, maximum *n*=133.	−3 Extremely serious	0	0	0	Very low	The sole criterion-related analysis was inadequate and based on a post hoc form evaluated in the derivation sample.
Hopkins Symptom Checklist revised 12-item depression scale (English)	Construct validity – hypotheses testing	+	Williams et al., 2016; post hoc form; maximum *n*=133; 8/8 hypotheses supported.	−3 Extremely serious	0	0	0	Very low	One inadequate post hoc analysis with no independent validation.
Hopkins Symptom Checklist revised 12-item depression scale (English)	Responsiveness	+	Williams et al., 2016; 133 participants/885 observations; treatment-effect differences ≤0.11 *SD*.	−3 Extremely serious	0	0	0	Very low	One inadequate post hoc repeated-measures analysis; repeated observations were not independent.
Patient Health Questionnaire-9, Arabic version (Lebanese Arabic)	Content validity – relevance	+	Summaka et al., 2019; 15-person SCI pilot of an existing Arabic translation.	−2 Very serious	0	NA	0	Low	Evidence came from one limited pilot rather than a full content-validity study. Sample size is part of content-validity RoB and was not graded again for imprecision.
Patient Health Questionnaire-9, Arabic version (Lebanese Arabic)	Content validity – comprehensiveness	+	Summaka et al., 2019; 15-person SCI pilot of an existing Arabic	−2 Very serious	0	NA	0	Low	One limited pilot; no SCI-specific concept elicitation or formal evaluation

PROM and version/form	Measurement property	Overall rating	Evidence base used for certainty assessment	Risk of bias	Inconsistency	Imprecision	Indirectness	Final certainty	Complete downgrading rationale
			translation.						of missing concepts.
Patient Health Questionnaire-9, Arabic version (Lebanese Arabic)	Content validity – comprehensibility	+	Summaka et al., 2019; *n*=15; no reported ambiguity; mean completion time 4–6 min.	−2 Very serious	0	NA	0	Low	One small pilot with limited detail on understanding of instructions and recall period; no independent replication.
Patient Health Questionnaire-9, Arabic version (Lebanese Arabic)	Reliability	+	Summaka et al., 2019; retest *n*=20; ICC=0.886.	−2 Very serious	0	0	0	Low	One doubtful-quality study. No additional imprecision downgrade was applied to avoid double counting the very small retest sample within the study-level methodological judgement.
Patient Health Questionnaire-9, Arabic version (Lebanese Arabic)	Construct validity – hypotheses testing	+	Summaka et al., 2019; *n*=51; convergent correlation and HDRS-A-defined known-groups comparison; 2/2 hypotheses supported.	−1 Serious	0	−1 Serious	0	Low	One adequate and one doubtful analysis from the same small sample; total *n* was between 50 and 100.
Patient Health Questionnaire-2 (English)	Criterion validity	-	Bombardier et al., 2012; *n*=142; blinded SCID comparison. Poritz et al., 2018 (*n*=116) used PHQ-9 as a non-independent proxy and was indeterminate.	−1 Serious	0	0	0	Moderate	The insufficient rating was determined by one adequate direct diagnostic study; the proxy comparison was excluded from the summarized rating.
SCL-90-R Cognitive/Affective Depression Research Subscale (English)	Construct validity – hypotheses testing	+	Buckelew et al., 1988; SCI *n*=52; one known-groups analysis. Pooled correlations were not SCI-specific and were excluded.	−2 Very serious	0	−1 Serious	0	Very low	One doubtful-quality study and an SCI-specific sample below 100.
SCL-90-R Somatic Depression Research Subscale (English)	Construct validity – hypotheses testing	-	Buckelew et al., 1988; SCI *n*=52; one known-groups analysis. Pooled correlations were not SCI-specific and were excluded.	−2 Very serious	0	−1 Serious	0	Very low	One doubtful-quality study and an SCI-specific sample below 100.
Inventory to Diagnose Depression total severity score	Structural validity	-	Frank et al., 1992; SCI *n*=134; one adequate EFA.	−1 Serious	0	NA	0	Moderate	One adequate-quality structural study. Imprecision is not separately

PROM and version/form	Measurement property	Overall rating	Evidence base used for certainty assessment	Risk of bias	Inconsistency	Imprecision	Indirectness	Final certainty	Complete downgrading rationale
(English)									graded for structural validity.
Inventory to Diagnose Depression total severity score (English)	Construct validity – hypotheses testing	-	Frank et al., 1992; SCI recruitment *n*=134 within a four-group analysis (total analyzable *n*≈456); 0/1 hypothesis supported.	−2 Very serious	0	0	−1 Serious	Very low	One doubtful analysis with unclear group-specific complete-case denominators; an additional indirectness downgrade reflected reliance on a mixed-population four-group comparison rather than a dedicated SCI validation design.
Inventory to Diagnose Depression Dysphoria factor score (English)	Construct validity – hypotheses testing	+	Frank et al., 1992; SCI recruitment *n*=134 within a four-group analysis (total analyzable *n*≈456); 1/1 hypothesis supported.	−2 Very serious	0	0	−1 Serious	Very low	One doubtful post hoc factor analysis with unclear group-specific complete-case denominators; evidence was indirect because the score was derived and compared within a mixed-population design.
Brief Symptom Inventory SCI-specific Depression Research Factor (7-item English)	Construct validity – hypotheses testing	+	Heinrich & Tate, 1996; follow-up *n*=204; rehospitalized versus non-rehospitalized comparison.	−2 Very serious	0	0	−1 Serious	Very low	One doubtful analysis; rehospitalization was an indirect proxy for depressive construct validity and rehospitalizations outside the study system were not captured.

Table SVIB. Indeterminate or unavailable summarized results not eligible for GRADE assessment
Under COSMIN methodology, an indeterminate summarized result (?) is not assigned a GRADE certainty level. This panel documents every such result and the reason that certainty was not graded.

Abbreviations: CFA, confirmatory factor analysis; COSMIN, COnsensus-based Standards for the selection of health Measurement INstruments; DIF, differential item functioning; GRADE, Grading of Recommendations Assessment, Development and Evaluation; HDRS-A, Arabic Hamilton Depression Rating Scale; IRT, item response theory; MIC, minimal important change; PHQ, Patient Health Questionnaire; RoB, risk of bias; SCID, Structured Clinical Interview for DSM; SCI, spinal cord injury.
An empty evidence domain in the main summary-of-findings table should therefore be interpreted as either no evidence or an indeterminate result, not as high certainty.

PROM and version/form	Measurement property	Summarized result/status	Evidence base	Reason no GRADE level was assigned
CES-D-20 (English)	Internal consistency	?	McColl & Skinner, 1992; 120 | Miller et al., 2008; 47	Cronbach's alpha=0.906 (*n*=120) and 0.91 (*n*=47); because CES-D-20 structural validity was insufficient, internal consistency remains indeterminate. COSMIN does not assign a GRADE level to an indeterminate (?) summarized result.
CES-D-20 (English)	Cross-cultural validity/measurement invariance	?	Cook et al., 2016; SCI 238; reference 726; total 964	No CES-D item met the authors' standard DIF thresholds, but exact item-level McFadden delta-R2 values were not reported; the COSMIN <0.02 criterion cannot be verified. COSMIN does not assign a GRADE level to an indeterminate (?) summarized result.
CES-D-20 (English)	Measurement error	?	Miller et al., 2008; 47	Mean difference=-1.30 (95% *CI* -2.76 to 0.24), with minimal bias; numeric LoA and MIC were not reported. COSMIN does not assign a GRADE level to an indeterminate (?) summarized result.
Zung Self-Rating Depression Scale (English)	Internal consistency	?	Tate et al., 1993; 162	Cronbach's alpha=0.81 (*n*=162), but structural validity was insufficient; internal consistency is indeterminate. COSMIN does not assign a GRADE level to an indeterminate (?) summarized result.
Zung Self-Rating Depression Scale (English)	Criterion validity	?	Tate et al., 1993; 30	Sensitivity 86%, specificity 61%, agreement 67%, kappa=0.33 against non-structured concordant clinician classification; no AUC/correlation. COSMIN does not assign a GRADE level to an indeterminate (?) summarized result.
Brief Symptom Inventory – Depression subscale (English)	Internal consistency	?	Tate et al., 1993; 162	Cronbach's alpha=0.87 (*n*=162), but structural validity of BSI-D was insufficient; internal consistency is indeterminate. COSMIN does not assign a GRADE level to an indeterminate (?) summarized result.
Brief Symptom Inventory – Depression subscale (English)	Criterion validity	?	Tate et al., 1993; 30 | Mitchell et al., 2008; 40	Tate et al. (1993): sensitivity 57%, specificity 87% against concordant clinician classification. Mitchell et al. (2008): MINI reference; traditional cutoff sensitivity/specificity 57%/82%, elevated cutoff 14%/97%. No eligible AUC or criterion correlation was reported. COSMIN does not assign a GRADE level to an indeterminate (?) summarized result.
Beck Depression Inventory-I (English)	Internal consistency	?	Radnitz et al., 1997; 124	Cronbach's alpha=0.89 (*n*=124); without sufficient structural-validity evidence, internal consistency is indeterminate. COSMIN does not assign a GRADE level to an indeterminate (?) summarized result.
Beck Depression Inventory-I (English)	Criterion validity	?	Radnitz et al., 1997; 124	SCID-*P* current MDE reference; 12/124 cases. At cut-off 18: sensitivity 83.3%, specificity 90.8%, 90.1% correctly classified; at cut-off 27: sensitivity 50.0%, specificity 100%, 95.0% in Table 2 (95.2% in text). No AUC/correlation; result indeterminate. COSMIN does not assign a GRADE level to an indeterminate (?) summarized result.

PROM and version/form	Measurement property	Summarized result/status	Evidence base	Reason no GRADE level was assigned
Patient Health Questionnaire-9 (English)	Internal consistency	?	Bombardier et al., 2004; 849 | Richardson & Richards, 2008; 2570 | Krause et al., 2008; 568 | Kalpakjian et al., 2009; RS1/RS2 *n*=584 each; sex-specific analyses *n*=292 each | Williams et al., 2009 - Rasch analysis; 202 | Krause et al., 2009; NR (classification *n*=690) | Williams et al., 2016 - original scoring; 133 participants / 884 observations | Poritz et al., 2017; 116	Poritz et al. (2017) added alpha=0.95, but cumulative original-PHQ-9 structural validity remains insufficient; total-score internal consistency remains indeterminate. COSMIN does not assign a GRADE level to an indeterminate (?) summarized result.
Patient Health Questionnaire-9 (English)	Cross-cultural validity/measurement invariance	?	Richardson & Richards, 2008; 2570 | Kalpakjian et al., 2009; 1,168 total; 292 women and 292 men in each random sample | Krause et al., 2010 - first follow-up EFA; 554, 211, and 302 | Krause et al., 2011 - full-sample CFA; 582-5,767 across strata | Cook et al., 2016; SCI 3,694; reference 3,000; total 6,694 | Graves & Bombardier, 2008 - one-factor CFA; Men 2,863; women 789 | Graves & Bombardier, 2008 - graded-response-model and subgroup-functioning analyses; 3652	Subgroup information-function comparisons suggested cumulative sex differences and little difference by race, injury duration or education, but formal COSMIN measurement-invariance testing was not performed. COSMIN does not assign a GRADE level to an indeterminate (?) summarized result.
Hospital Anxiety and Depression Scale - Depression subscale (English)	Cross-cultural validity/measurement invariance	?	Woolrich et al., 2006; 963	Separate subgroup PCAs suggested item instability, but formal measurement invariance/DIF was not tested; the result is indeterminate. COSMIN does not assign a GRADE level to an indeterminate (?) summarized result.
CES-D-10 (English)	Internal consistency	?	Miller et al., 2008; 47	Cronbach's alpha=0.86 (*n*=47); without structural-validity evidence, internal consistency is indeterminate. COSMIN does not assign a GRADE level to an indeterminate (?) summarized result.
CES-D-10 (English)	Measurement error	?	Miller et al., 2008; 47	Mean difference=-0.22 (95% *CI* -1.21 to 0.78), with minimal bias; numeric LoA and MIC were not reported. COSMIN does not assign a GRADE level to an indeterminate (?) summarized result.
CES-D-10 (English)	Construct validity – hypotheses testing	?	Miller et al., 2008; 47	VAS-*F* *r*=0.57 supported the prespecified hypothesis, but the SF-36 signs conflict between Table 3 and the narrative; overall construct-validity evidence from this report is indeterminate. COSMIN does not assign a GRADE level to an indeterminate (?) summarized result.
Depression Anxiety Stress Scales-21 - Depression subscale (English)	Criterion validity	?	Mitchell et al., 2008; 40	MINI reference, 7/40 depression diagnoses: sensitivity 57%, specificity 76%; no eligible AUC or criterion correlation. COSMIN does not assign a GRADE level to an indeterminate (?) summarized result.
Older Adult Health and Mood Questionnaire (English)	Internal consistency	?	Krause et al., 2009; NR (classification *n*=682)	Alpha=0.87, but no structural-validity evidence was available for the OAHMQ in this report; internal consistency is indeterminate. COSMIN does not assign a GRADE level to an indeterminate (?) summarized result.
Hospital Anxiety and Depression Scale - Depression subscale (German)	Structural validity	?	Müller et al., 2012; 100	Rasch item fit, residual PCA and ordered thresholds were favourable, but local independence, monotonicity and COSMIN-specified unidimensionality statistics were not reported. COSMIN does not assign a GRADE level to an indeterminate (?) summarized result.

PROM and version/form	Measurement property	Summarized result/status	Evidence base	Reason no GRADE level was assigned
Hospital Anxiety and Depression Scale - Depression subscale (German)	Internal consistency	?	Müller et al., 2012; 100	Person reliability index=0.82, but sufficient unidimensionality and Cronbach alpha/omega were not established. COSMIN does not assign a GRADE level to an indeterminate (?) summarized result.
Hospital Anxiety and Depression Scale - Depression subscale (German)	Cross-cultural validity/measurement invariance	?	Müller et al., 2012; 100	No DIF was statistically detected, but subgroup sample sizes were inadequate and McFadden R2 effect sizes were not reported. COSMIN does not assign a GRADE level to an indeterminate (?) summarized result.
Hospital Anxiety and Depression Scale - Depression subscale (Turkish)	Internal consistency	?	Paker et al., 2013; 175	Cronbach's alpha=0.77, but structural validity of the Turkish HADS-D was not evaluated and evidence from other language versions was not transferred. COSMIN does not assign a GRADE level to an indeterminate (?) summarized result.
SCI-QOL Depression Item Bank (English)	Content validity – comprehensibility	?	Tulsky et al., 2015; cognitive debriefing was reported, but understanding of instructions, recall period, and response-option matching was not sufficiently documented.	The summarized result was indeterminate; COSMIN does not assign a GRADE level to an indeterminate (?) result.
SCI-QOL Depression Short Form 10a (English)	All measurement properties	No evidence	Tulsky et al., 2015; 10-item form derived from the calibrated item bank and accompanied by a raw-score/T-score lookup table.	The short form was not independently evaluated for any COSMIN measurement property in the included report; no summarized result or GRADE assessment was possible.
Patient Health Questionnaire-9, revised 3-category scoring (English)	Structural validity	?	Williams et al., 2016 - post hoc revised scoring; 133 participants / 884 observations	Rasch structural evidence was indeterminate because local independence/monotonicity and independent validation were lacking. COSMIN does not assign a GRADE level to an indeterminate (?) summarized result.
Patient Health Questionnaire-9, revised 3-category scoring (English)	Internal consistency	?	Williams et al., 2016 - post hoc revised scoring; 133 participants / 884 observations	Alpha=0.82 and person-separation reliability=0.77, but sufficient structural validity was not established. COSMIN does not assign a GRADE level to an indeterminate (?) summarized result.
Hopkins Symptom Checklist-20 Depression Scale, Version B (English)	Internal consistency	?	Williams et al., 2016 - original scoring; 133 participants / 885 observations	Alpha=0.91 and person-separation reliability=0.82, but structural validity was insufficient. COSMIN does not assign a GRADE level to an indeterminate (?) summarized result.
Hopkins Symptom Checklist revised 12-item depression scale (English)	Internal consistency	?	Williams et al., 2016 - post hoc revised scoring; 133 participants / 885 observations	Alpha=0.90 and person-separation reliability=0.84, but structural validity was insufficient. COSMIN does not assign a GRADE level to an indeterminate (?) summarized result.
PROMIS Depression Short Form 8b, version 1.0 (English)	Cross-cultural validity/measurement invariance	?	Cook et al., 2016; SCI 292; reference 761; total 1,053	No PROMIS-D item met the authors' standard DIF thresholds, but exact item-level McFadden delta-R2 values were not reported; the COSMIN <0.02 criterion cannot be verified. COSMIN does not assign a GRADE level to an indeterminate (?) summarized result.
Patient Health Questionnaire-9, Arabic version (Lebanese Arabic)	Structural validity	?	Summaka et al., 2019 - factor analysis, internal consistency, retest reliability, ROC and convergent validity; 51	Exploratory PCA yielded three factors explaining 66.2%; no confirmatory evidence established the intended total-score structure. COSMIN does not assign a GRADE level to an indeterminate (?) summarized result.
Patient Health Questionnaire-9, Arabic version (Lebanese Arabic)	Internal consistency	?	Summaka et al., 2019 - factor analysis, internal consistency, retest reliability, ROC and convergent validity; 51	Alpha=0.711, but sufficient unidimensionality was not established. COSMIN does not assign a GRADE level to an indeterminate (?) summarized result.

PROM and version/form	Measurement property	Summarized result/status	Evidence base	Reason no GRADE level was assigned
Patient Health Questionnaire-9, Arabic version (Lebanese Arabic)	Criterion validity	?	Summaka et al., 2019 - factor analysis, internal consistency, retest reliability, ROC and convergent validity; 51	ROC AUC=0.83 with sensitivity 83% and specificity 69%, but HDRS-A cutoff classification was not an accepted diagnostic gold standard. COSMIN does not assign a GRADE level to an indeterminate (?) summarized result.
Patient Health Questionnaire-2 (English)	Structural validity	?	Graves & Bombardier, 2008; 3652	Relative efficiency was 0.46, but no complete COSMIN structural-validity evaluation of the two-item form was reported. COSMIN does not assign a GRADE level to an indeterminate (?) summarized result.
Patient Health Questionnaire-2 (English)	Internal consistency	?	Poritz et al., 2017; 116	Alpha=0.91, but PHQ-2 unidimensional structural validity has not been established in the available SCI evidence; internal consistency remains indeterminate. COSMIN does not assign a GRADE level to an indeterminate (?) summarized result.
PHQ-9-derived 3-item short form (English)	Structural validity	?	Graves & Bombardier, 2008; 3652	The post hoc three-item form retained 0.67/0.66 of full-scale information, but item selection and evaluation used the same sample and complete COSMIN IRT evidence was absent. COSMIN does not assign a GRADE level to an indeterminate (?) summarized result.
PHQ-9-derived 3-item short form (English)	Cross-cultural validity/measurement invariance	?	Graves & Bombardier, 2008; Men 2,863; women 789	Sex-related information-function differences were smaller than for the PHQ-9, but no formal multi-group CFA or item-level DIF criterion was used. COSMIN does not assign a GRADE level to an indeterminate (?) summarized result.
PHQ-9-derived 3-item short form (English)	Criterion validity	?	Graves & Bombardier, 2008; 3652	Cutoffs >=3 and >=4 had high agreement with parent PHQ-9 >=10, but the overlapping parent scale was not an independent diagnostic criterion. COSMIN does not assign a GRADE level to an indeterminate (?) summarized result.
SCL-90-R Cognitive/Affective Depression Research Subscale (English)	Internal consistency	?	Buckelew et al., 1988 - SCI internal consistency and known-groups validity; 52	SCI alpha=.89 with complete SCI item-total correlations, but no sufficient structural-validity evidence was available. COSMIN does not assign a GRADE level to an indeterminate (?) summarized result.
SCL-90-R Somatic Depression Research Subscale (English)	Internal consistency	?	Buckelew et al., 1988 - SCI internal consistency and known-groups validity; 52	SCI alpha=.62; item 60 was weak, but no sufficient structural-validity evidence and no validated six-item recalculation were available. COSMIN does not assign a GRADE level to an indeterminate (?) summarized result.
Inventory to Diagnose Depression total severity score (English)	Cross-cultural validity/measurement invariance	?	Frank et al., 1992; Total 502; SCI 134	Factor-pattern similarity was assessed with salient-variable indices rather than formal multigroup CFA or DIF. COSMIN does not assign a GRADE level to an indeterminate (?) summarized result.
Inventory to Diagnose Depression Dysphoria factor score (English)	Structural validity	?	Frank et al., 1992; 134	Post hoc nine-item factor was not independently tested as a unidimensional score. COSMIN does not assign a GRADE level to an indeterminate (?) summarized result.
Inventory to Diagnose Depression Dysphoria factor score (English)	Cross-cultural validity/measurement invariance	?	Frank et al., 1992; Total 502; SCI 134	Cross-population factor-pattern similarity was reported, but formal measurement-invariance methods were absent. COSMIN does not assign a GRADE level to an indeterminate (?) summarized result.
Brief Symptom Inventory SCI-specific Depression Research Factor (7-item English)	Structural validity	?	Heinrich & Tate, 1996; 215	Post hoc seven-item factor from one inadequate-quality EFA; no independent confirmatory validation. COSMIN does not assign a GRADE level to an indeterminate (?) summarized result.
Brief Symptom Inventory SCI-specific Depression Research Factor (7-item English)	Internal consistency	?	Heinrich & Tate, 1996; 215	Alpha=.88, but sufficient unidimensional structural validity was not established. COSMIN does not assign a GRADE level to an indeterminate (?) summarized result.

Table SVII. Reviewer-based and integrated content-validity assessment of included PROM versions/forms

COSMIN rules applied. Ratings were made for depressive symptoms in adults with traumatic or non-traumatic SCI and the stated context of use. Criteria 1–5 determine relevance, criterion 6 determines comprehensiveness, and criteria 9–10 determine reviewer-rated comprehensibility; criteria 7–8 are not used for the review-team rating. When no eligible study-based evidence was available, conclusions based solely on consensus reviewer ratings were assigned very-low certainty; indeterminate results were not graded. Study-based evidence was identified only for the SCI-QOL Depression Item Bank and for patient-rated comprehensibility of the Lebanese Arabic PHQ-9. Two reviewers independently completed criterion-level ratings for each PROM version/form for all assessable aspects and resolved disagreements by consensus, with third-reviewer adjudication when required. For non-English versions, reviewer-rated comprehensibility was recorded only when adequate language proficiency was available. Tables SVIIA and SVIIB present the final consensus and integrated ratings.

Rating key: + sufficient; - insufficient; ± inconsistent or mixed; ? indeterminate. C1 item relevance to construct; C2 relevance to target population; C3 relevance to context; C4 response options; C5 recall period; C6 no key concepts missing; C9 appropriate wording; C10 response options match questions. R, relevance; C, comprehensiveness; U, comprehensibility.

Table SVIIA. Consensus criterion-level reviewer assessments and derived ratings

PROM/version/form	Intended context	Study-based content-validity evidence	Instrument material/status	C1 / C2 / C3 / C4 / C5	C6	C9 / C10	Derived R / C / U	Key rationale and limitations
CES-D-20 (English)	Screening and depressive-symptom severity assessment	No eligible SCI-specific PROM-development or content-validity study.	Complete 20-item form, response options, recall period, and scoring available.	+ / + / - / + / +	+	+ / +	± / + / +	Broad coverage of depressive symptoms. Appetite, effort, sleep, and reduced activation items may be influenced by SCI, pain, medication, or secondary complications; therefore relevance is context-dependent rather than unequivocally sufficient.
Zung Self-Rating Depression Scale (English)	Screening and severity assessment	No eligible SCI-specific PROM-development or content-validity study.	Complete 20-item form and four-category response structure available.	+ / - / - / + / ?	+	+ / +	- / + / +	The scale is broad and understandable, but several items (sleep, appetite/weight, constipation, heart rate, fatigue, sexual interest, and usual activities) are poorly specific to depression in adults with SCI.
Brief Symptom Inventory–Depression subscale (English)	Depressive-distress screening within a multidomain symptom inventory	No eligible SCI-specific PROM-development or content-validity study.	Complete six-item depression subscale and standard BSI response options identifiable.	+ / + / + / + / +	-	+ / +	+ / - / +	The six affective/cognitive items are relevant and clear, but the subscale omits multiple important depressive manifestations and is not comprehensive for the broad depressive-symptom construct.
Beck Depression Inventory-I (English)	Screening and depressive-symptom severity assessment	No eligible SCI-specific PROM-development or content-validity study.	Complete 21-item form and item-specific graded response statements available.	+ / + / - / + / +	+	+ / +	± / + / +	The measure covers cognitive, affective, behavioural, and somatic symptoms. Its sleep, appetite, weight, fatigue, work, somatic-preoccupation, and libido content may be confounded by SCI; item-specific graded statements are nevertheless interpretable.

PROM/version/form	Intended context	Study-based content-validity evidence	Instrument material/status	C1 / C2 / C3 / C4 / C5	C6	C9 / C10	Derived R / C / U	Key rationale and limitations
Ilfeld Psychiatric Symptom Index–Depression subscale (English)	Depressive-symptom screening	No eligible SCI-specific PROM-development or content-validity study.	All 29 PSI items were reproduced, but the exact independently scored 10-item depression subset was not explicitly demarcated and was inferred from item grouping.	? / ? / ? / + / ?	?	? / ?	? / ? / ?	A defensible item-level assessment requires an authoritative specification of the independently scored depression subset. The included report also identified substantial somatic contamination in the PSI.
Patient Health Questionnaire-9 (English)	Probable-MDD screening and depressive-symptom severity assessment	No eligible SCI-specific PROM-development or content-validity study.	Complete nine-item form, response options, 2-week recall period, and scoring available.	+ / + / - / + / +	+	+ / +	± / + / +	All nine DSM depressive-symptom domains are represented and wording is clear. Sleep, energy, appetite, concentration, and psychomotor items may reflect SCI, pain, medication, or rehabilitation context, making relevance context-dependent.
Hospital Anxiety and Depression Scale–Depression subscale (English)	Screening when limiting somatic symptom overlap is important	No eligible SCI-specific PROM-development or content-validity study.	Complete seven-item English HADS-D form available.	+ / + / + / + / +	-	+ / +	+ / - / +	The focus on anhedonia and low mood is relevant to SCI and limits somatic overlap. The seven-item subscale omits several cognitive, behavioural, vegetative, and suicidality-related manifestations of the broad construct.
CES-D-10 (English)	Brief screening and depressive-symptom assessment	No eligible SCI-specific PROM-development or content-validity study.	Complete 10-item short form and scoring available.	+ / + / - / + / +	-	+ / +	± / - / +	The short form contains relevant mood, positive-affect, interpersonal, and somatic content, but effort/sleep/reduced-activation content may be confounded and the abbreviated form omits important depressive domains.
DASS-21 Depression subscale (English)	Brief depressive-symptom severity assessment	No eligible SCI-specific PROM-development or content-validity study.	Complete seven-item depression subscale, past-week recall, and response options available.	+ / + / + / + / +	-	+ / +	+ / - / +	Affective and cognitive items are relevant and avoid most direct somatic confounding. The subscale is narrow and does not cover several clinically important depressive manifestations.
Older Adult Health and Mood Questionnaire (English)	Screening with limited vegetative content	No eligible SCI-specific PROM-development or content-validity study.	The included report described the 22-item true/false form and its rationale but did not reproduce the complete authoritative item set.	? / ? / ? / + / ?	?	? / ?	? / ? / ?	The instrument was designed for older adults and people with physical disability and intentionally limits vegetative content, but the 85% item-level COSMIN criteria cannot be applied without the complete authoritative form.

PROM/version/form	Intended context	Study-based content-validity evidence	Instrument material/status	C1 / C2 / C3 / C4 / C5	C6	C9 / C10	Derived R / C / U	Key rationale and limitations
Hospital Anxiety and Depression Scale–Depression subscale (German)	German-language screening and severity assessment	No eligible SCI-specific PROM-development or content-validity study.	German HADS-D structure and scoring available; language-specific comprehensibility requires a German-proficient reviewer.	+ / + / + / + / +	-	? / ?	+ / - / ?	Conceptual content mirrors the English HADS-D and limits somatic overlap, but it remains incomplete for broad depressive symptoms. German-language wording cannot be rated without documented proficiency.
Hospital Anxiety and Depression Scale–Depression subscale (Turkish)	Turkish-language screening and severity assessment	No eligible SCI-specific PROM-development or content-validity study.	Turkish HADS-D structure and scoring available; language-specific comprehensibility requires a Turkish-proficient reviewer.	+ / + / + / + / +	-	? / ?	+ / - / ?	Conceptual content mirrors the English HADS-D and limits somatic overlap, but it remains incomplete for broad depressive symptoms. Turkish-language wording cannot be rated without documented proficiency.
SCI-QOL Depression Item Bank (English)	SCI-specific depressive-symptom severity assessment	Tulsky et al. (2015): SCI-specific PROM development supported relevance and comprehensiveness; study-based comprehensibility was indeterminate because reporting of qualitative testing was incomplete.	Complete calibrated 28-item bank, 7-day recall, response options, and development documentation available.	+ / + / + / + / +	+	+ / +	+ / + / +	Items were generated and reviewed with people with SCI and clinicians, cover the intended cognitive-affective depression domain, and deliberately limit confounded somatic content. Under COSMIN, the indeterminate study rating for comprehensibility does not prevent a sufficient summarized rating when the reviewer criteria 9 and 10 are both sufficient.
SCI-QOL Depression CAT (English)	Computer-adaptive SCI-specific severity assessment	No separate CAT-specific content-validity study; indirect development evidence derives from the calibrated item bank.	Item bank, response options, CAT algorithm, and stopping rules available.	+ / + / + / + / +	?	+ / +	+ / ? / +	Every administered item comes from the relevant SCI-specific bank and uses clear response options. The content represented in a short adaptive administration varies by severity, so form-level comprehensiveness cannot be established confidently.
SCI-QOL Depression Short Form 10a (English)	Fixed short-form SCI-specific severity assessment	No short-form-specific content-validity study; indirect development evidence derives from the calibrated item bank.	Complete fixed 10-item form, instructions, response options, and scoring table available.	+ / + / + / + / +	?	+ / +	+ / ? / +	The selected items are relevant and clearly worded. They were selected mainly for measurement information rather than a documented form-specific content-coverage exercise, so comprehensiveness remains indeterminate.

PROM/version/form	Intended context	Study-based content-validity evidence	Instrument material/status	C1 / C2 / C3 / C4 / C5	C6	C9 / C10	Derived R / C / U	Key rationale and limitations
PHQ-9, revised 3-category scoring (English)	Post hoc treatment-monitoring score	No eligible form-specific PROM-development or content-validity study.	Original PHQ-9 item content retained; the two intermediate response categories were collapsed post hoc.	+ / + / - / + / +	+	+ / +	± / + / +	Content coverage is identical to the PHQ-9 and therefore retains the same SCI-related symptom-attribution concern. The collapsed response categories are understandable but were not developed through a form-specific content-validity process.
Hopkins Symptom Checklist-20 Depression Scale, Version B (English)	Depressive-symptom severity and treatment monitoring	No eligible SCI-specific PROM-development or content-validity study.	All 20 item stems, five response options, and past-week recall available.	+ / + / - / + / +	+	+ / +	± / + / +	The measure is broad and understandable, but several somatic and functional items may reflect SCI or treatment context rather than depression.
HSCL revised 12-item depression scale (English)	Post hoc treatment-monitoring score	No eligible form-specific PROM-development or content-validity study.	The report stated a 12-item form, but the exact retained-item set and reproducible scoring algorithm could not be reconstructed.	? / ? / ? / ? / +	?	? / ?	? / ? / ?	Without a verifiable final item set, relevance, comprehensiveness, and comprehensibility cannot be assessed reliably.
PROMIS Depression Short Form 8b, version 1.0 (English)	Brief depressive-symptom severity assessment	No eligible SCI-specific PROM-development or content-validity study.	The fixed eight-item form and standard five-category response structure were identifiable.	+ / + / + / + / +	-	+ / +	+ / - / +	Affective/cognitive items are highly relevant and avoid direct somatic confounding. The eight-item form does not represent the full broad depressive-symptom spectrum, including suicidality and vegetative manifestations.
Patient Health Questionnaire-9 (Lebanese Arabic)	Arabic-language screening and severity assessment	Summaka et al. (2019): a 15-person pilot directly supported comprehensibility only; no SCI-specific relevance or comprehensiveness study was performed.	Complete Lebanese Arabic nine-item form, response options, 2-week recall period, and scoring were available for reviewer assessment.	+ / + / + / + / +	+	+ / +	+ / + / +	The version retains all nine PHQ-9 depressive-symptom domains, with appropriate response options and recall period. The 15-person pilot reported no comprehension problems. Somatic and functional symptoms may require clinical attribution in SCI, but this affects interpretive specificity rather than their relevance to the depressive-symptom construct. The pilot did not directly evaluate relevance or comprehensiveness.
Patient Health Questionnaire-2 (English)	Ultra-brief first-stage prescreen	No eligible SCI-specific PROM-development or content-validity study.	Complete two-item form and standard PHQ response options available.	+ / + / + / + / +	-	+ / +	+ / - / +	Depressed mood and anhedonia are relevant and clear for a first-stage prescreen, but two items are not comprehensive for the broad depressive-symptom construct or for severity assessment.

PROM/version/form	Intended context	Study-based content-validity evidence	Instrument material/status	C1 / C2 / C3 / C4 / C5	C6	C9 / C10	Derived R / C / U	Key rationale and limitations
PHQ-9-derived 3-item short form (English)	Brief first-stage screen for probable depression	No eligible form-specific PROM-development or content-validity study.	Complete three-item form comprising PHQ-9 items 1, 2, and 6 available.	+ / + / + / + / +	-	+ / +	+ / - / +	The three psychological symptoms are relevant, clear, and less vulnerable to somatic confounding, but they cannot comprehensively represent the broad depressive-symptom construct.
SCL-90-R Cognitive/Affective Depression research subscale (English)	Research assessment of cognitive/affective depressive distress	No eligible SCI-specific PROM-development or content-validity study.	Experimental 13-item subset and standard response options identifiable from the report.	+ / + / + / + / +	-	+ / +	+ / - / +	Cognitive and affective content is relevant and avoids most direct somatic overlap, but the research subscale does not comprehensively cover the full depressive-symptom spectrum.
SCL-90-R Somatic Depression research subscale (English)	Research assessment of somatic depressive distress	No eligible SCI-specific PROM-development or content-validity study.	Experimental seven-item somatic subset and standard response options identifiable from the report.	+ / - / - / + / +	-	+ / +	- / - / +	Somatic content is highly vulnerable to direct effects of SCI, pain, medication, and rehabilitation and is not sufficient to represent depression as a whole; wording and response options are understandable.
Inventory to Diagnose Depression total severity score (English)	Depressive-syndrome classification and severity assessment	No eligible SCI-specific PROM-development or content-validity study.	The 22 symptom domains were reported, but complete instructions, response categories, and the detailed scoring algorithm were unavailable in the included report.	+ / + / - / ? / ?	+	? / ?	± / + / ?	DSM-based content is broad, but multiple somatic and functional symptoms may be confounded by SCI. Comprehensibility cannot be rated confidently without the authoritative administration form.
Inventory to Diagnose Depression Dysphoria factor score (English)	Research assessment of core dysphoria	No eligible form-specific PROM-development or content-validity study.	Nine factor items were identifiable, but complete item-response instructions and an independently administered form were unavailable.	+ / + / + / ? / ?	-	? / ?	± / - / ?	The post hoc factor represents relevant cognitive/affective dysphoria but is not comprehensive for broad depressive symptoms. Unknown response and administration details prevent a comprehensibility judgement.
Brief Symptom Inventory SCI-specific Depression Research Factor, 7-item (English)	Research/clinical interpretation during initial SCI rehabilitation	No eligible independent content-validity study; the factor was empirically derived in an SCI sample.	Seven salient items and standard BSI response options were reported.	+ / + / + / + / +	-	+ / +	+ / - / +	The SCI-derived affective/cognitive items are relevant and understandable, but the narrow seven-item factor does not comprehensively represent the full depressive-symptom construct.

Table SVIIB. Integrated content-validity ratings and certainty by aspect

Notes. *Overall content validity is shown only as an orientation using the COSMIN combination rule: + when R, C, and U are all +; - when all are -; ± when sufficient/inconsistent and insufficient/inconsistent ratings coexist; and ? when one or more aspects are indeterminate. The three aspects should remain visible separately in the manuscript and recommendation process. The very-low certainty assigned to reviewer-only conclusions reflects the absence of eligible study-based content-validity evidence, not necessarily poor content. For German and Turkish forms, reviewer-rated comprehensibility remains indeterminate unless a proficient reviewer evaluates the actual language version. For the Lebanese Arabic PHQ-9, the 15-person patient pilot directly supports comprehensibility only; certainty is low for comprehensibility and very low for reviewer-only relevance and comprehensiveness. For the SCI-QOL Depression Item Bank, study-based comprehensibility was indeterminate, but the reviewer rating is sufficient because criteria 9 and 10 are sufficient; under COSMIN, an indeterminate study rating may be ignored when forming the summarized rating.

PROM/version/form	Integrated evidence basis	Relevance	Comprehensiveness	Comprehensibility	Overall content-validity orientation*	Interpretive note
CES-D-20 (English)	Consensus reviewer assessment only	± (Very low)	+ (Very low)	+ (Very low)	±	Broad coverage of depressive symptoms. Appetite, effort, sleep, and reduced activation items may be influenced by SCI, pain, medication, or secondary complications; therefore relevance is context-dependent rather than unequivocally sufficient.
Zung Self-Rating Depression Scale (English)	Consensus reviewer assessment only	- (Very low)	+ (Very low)	+ (Very low)	±	The scale is broad and understandable, but several items (sleep, appetite/weight, constipation, heart rate, fatigue, sexual interest, and usual activities) are poorly specific to depression in adults with SCI.
Brief Symptom Inventory–Depression subscale (English)	Consensus reviewer assessment only	+ (Very low)	- (Very low)	+ (Very low)	±	The six affective/cognitive items are relevant and clear, but the subscale omits multiple important depressive manifestations and is not comprehensive for the broad depressive-symptom construct.
Beck Depression Inventory-I (English)	Consensus reviewer assessment only	± (Very low)	+ (Very low)	+ (Very low)	±	The measure covers cognitive, affective, behavioural, and somatic symptoms. Its sleep, appetite, weight, fatigue, work, somatic-preoccupation, and libido content may be confounded by SCI; item-specific graded statements are nevertheless interpretable.
Ilfeld Psychiatric Symptom Index–Depression subscale (English)	Consensus reviewer assessment only; form not authoritatively verified	? (not graded)	? (not graded)	? (not graded)	?	A defensible item-level assessment requires an authoritative specification of the independently scored depression subset. The included report also identified substantial somatic contamination in the PSI.
Patient Health Questionnaire-9 (English)	Consensus reviewer assessment only	± (Very low)	+ (Very low)	+ (Very low)	±	All nine DSM depressive-symptom domains are represented and wording is clear. Sleep, energy, appetite, concentration, and psychomotor items may reflect SCI, pain, medication, or rehabilitation context, making relevance context-dependent.

PROM/version/form	Integrated evidence basis	Relevance	Comprehensiveness	Comprehensibility	Overall content-validity orientation*	Interpretive note
Hospital Anxiety and Depression Scale–Depression subscale (English)	Consensus reviewer assessment only	+ (Very low)	- (Very low)	+ (Very low)	±	The focus on anhedonia and low mood is relevant to SCI and limits somatic overlap. The seven-item subscale omits several cognitive, behavioural, vegetative, and suicidality-related manifestations of the broad construct.
CES-D-10 (English)	Consensus reviewer assessment only	± (Very low)	- (Very low)	+ (Very low)	±	The short form contains relevant mood, positive-affect, interpersonal, and somatic content, but effort/sleep/reduced-activation content may be confounded and the abbreviated form omits important depressive domains.
DASS-21 Depression subscale (English)	Consensus reviewer assessment only	+ (Very low)	- (Very low)	+ (Very low)	±	Affective and cognitive items are relevant and avoid most direct somatic confounding. The subscale is narrow and does not cover several clinically important depressive manifestations.
Older Adult Health and Mood Questionnaire (English)	Consensus reviewer assessment only; complete form unavailable in included evidence	? (not graded)	? (not graded)	? (not graded)	?	The instrument was designed for older adults and people with physical disability and intentionally limits vegetative content, but the 85% item-level COSMIN criteria cannot be applied without the complete authoritative form.
Hospital Anxiety and Depression Scale–Depression subscale (German)	Consensus reviewer assessment only	+ (Very low)	- (Very low)	? (not graded)	?	Conceptual content mirrors the English HADS-D and limits somatic overlap, but it remains incomplete for broad depressive symptoms. German-language wording cannot be rated without documented proficiency.
Hospital Anxiety and Depression Scale–Depression subscale (Turkish)	Consensus reviewer assessment only	+ (Very low)	- (Very low)	? (not graded)	?	Conceptual content mirrors the English HADS-D and limits somatic overlap, but it remains incomplete for broad depressive symptoms. Turkish-language wording cannot be rated without documented proficiency.
SCI-QOL Depression Item Bank (English)	SCI-specific development evidence plus consensus reviewer assessment	+ (Low)	+ (Low)	+ (Very low)	+	Items were generated and reviewed with people with SCI and clinicians, cover the intended cognitive-affective depression domain, and deliberately limit confounded somatic content. Under COSMIN, the indeterminate study rating for comprehensibility does not prevent a sufficient summarized rating when the reviewer criteria 9 and 10 are both sufficient.

PROM/version/form	Integrated evidence basis	Relevance	Comprehensiveness	Comprehensibility	Overall content-validity orientation*	Interpretive note
SCI-QOL Depression CAT (English)	Indirect item-bank evidence plus consensus reviewer assessment	+ (Very low)	? (not graded)	+ (Very low)	?	Every administered item comes from the relevant SCI-specific bank and uses clear response options. The content represented in a short adaptive administration varies by severity, so form-level comprehensiveness cannot be established confidently.
SCI-QOL Depression Short Form 10a (English)	Indirect item-bank evidence plus consensus reviewer assessment	+ (Very low)	? (not graded)	+ (Very low)	?	The selected items are relevant and clearly worded. They were selected mainly for measurement information rather than a documented form-specific content-coverage exercise, so comprehensiveness remains indeterminate.
PHQ-9, revised 3-category scoring (English)	Consensus reviewer assessment only	± (Very low)	+ (Very low)	+ (Very low)	±	Content coverage is identical to the PHQ-9 and therefore retains the same SCI-related symptom-attribution concern. The collapsed response categories are understandable but were not developed through a form-specific content-validity process.
Hopkins Symptom Checklist-20 Depression Scale, Version B (English)	Consensus reviewer assessment only	± (Very low)	+ (Very low)	+ (Very low)	±	The measure is broad and understandable, but several somatic and functional items may reflect SCI or treatment context rather than depression.
HSCL revised 12-item depression scale (English)	Consensus reviewer assessment not possible from available form information	? (not graded)	? (not graded)	? (not graded)	?	Without a verifiable final item set, relevance, comprehensiveness, and comprehensibility cannot be assessed reliably.
PROMIS Depression Short Form 8b, version 1.0 (English)	Consensus reviewer assessment only	+ (Very low)	- (Very low)	+ (Very low)	±	Affective/cognitive items are highly relevant and avoid direct somatic confounding. The eight-item form does not represent the full broad depressive-symptom spectrum, including suicidality and vegetative manifestations.
Patient Health Questionnaire-9 (Lebanese Arabic)	Consensus reviewer assessment for relevance and comprehensiveness; 15-person patient pilot plus consensus reviewer assessment for comprehensibility	+ (Very low)	+ (Very low)	+ (Low)	+	The version retains all nine PHQ-9 depressive-symptom domains. The 15-person pilot found no comprehension problems and directly supports comprehensibility only. Relevance and comprehensiveness are based on the two-reviewer consensus and therefore remain very-low-certainty conclusions.

PROM/version/form	Integrated evidence basis	Relevance	Comprehensiveness	Comprehensibility	Overall content-validity orientation*	Interpretive note
Patient Health Questionnaire-2 (English)	Consensus reviewer assessment only	+ (Very low)	- (Very low)	+ (Very low)	±	Depressed mood and anhedonia are relevant and clear for a first-stage prescreen, but two items are not comprehensive for the broad depressive-symptom construct or for severity assessment.
PHQ-9-derived 3-item short form (English)	Consensus reviewer assessment only	+ (Very low)	- (Very low)	+ (Very low)	±	The three psychological symptoms are relevant, clear, and less vulnerable to somatic confounding, but they cannot comprehensively represent the broad depressive-symptom construct.
SCL-90-R Cognitive/Affective Depression research subscale (English)	Consensus reviewer assessment only	+ (Very low)	- (Very low)	+ (Very low)	±	Cognitive and affective content is relevant and avoids most direct somatic overlap, but the research subscale does not comprehensively cover the full depressive-symptom spectrum.
SCL-90-R Somatic Depression research subscale (English)	Consensus reviewer assessment only	- (Very low)	- (Very low)	+ (Very low)	±	Somatic content is highly vulnerable to direct effects of SCI, pain, medication, and rehabilitation and is not sufficient to represent depression as a whole; wording and response options are understandable.
Inventory to Diagnose Depression total severity score (English)	Consensus reviewer assessment only; administration form incomplete	± (Very low)	+ (Very low)	? (not graded)	?	DSM-based content is broad, but multiple somatic and functional symptoms may be confounded by SCI. Comprehensibility cannot be rated confidently without the authoritative administration form.
Inventory to Diagnose Depression Dysphoria factor score (English)	Consensus reviewer assessment only; independent form incomplete	± (Very low)	- (Very low)	? (not graded)	?	The post hoc factor represents relevant cognitive/affective dysphoria but is not comprehensive for broad depressive symptoms. Unknown response and administration details prevent a comprehensibility judgement.
Brief Symptom Inventory SCI-specific Depression Research Factor, 7-item (English)	Consensus reviewer assessment only	+ (Very low)	- (Very low)	+ (Very low)	±	The SCI-derived affective/cognitive items are relevant and understandable, but the narrow seven-item factor does not comprehensively represent the full depressive-symptom construct.
